# Stereoselective Synthesis of Either *Exo-* or *Endo-*3-Azabicyclo[3.1.0]hexane-6-carboxylates by Dirhodium(II)-Catalyzed Cyclopropanation with Ethyl Diazoacetate under Low Catalyst Loadings

**DOI:** 10.1021/acs.orglett.3c03652

**Published:** 2024-01-03

**Authors:** Terrence-Thang H. Nguyen, Antonio Navarro, J. Craig Ruble, Huw M. L. Davies

**Affiliations:** † Department of Chemistry, Emory University, Atlanta, Georgia 30322, United States; ‡ Lilly Research Laboratories, Eli Lilly and Company, Indianapolis, Indiana 46285, United States

## Abstract

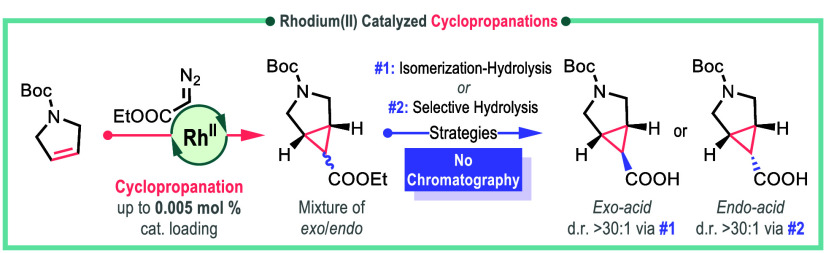

Although cyclopropanation with donor/acceptor carbenes can be conducted under low catalyst loadings (<0.001 mol %), such low loading has not been generally effective for other classes of carbenes such as acceptor carbenes. In this current study, we demonstrate that ethyl diazoacetate can be effectively used in the cyclopropanation of *N*-Boc-2,5-dihydropyrrole with dirhodium(II) catalyst loadings of 0.005 mol %. By appropriate choice of catalyst and hydrolysis conditions, either the *exo*- or *endo*-3-azabicyclo[3.1.0]hexanes can be formed cleanly with high levels of diastereoselectivity with no chromatographic purification.

Saturated nitrogen heterocycles are ubiquitous in pharmaceutical drugs and play an increasing role in drug discovery with the current emphasis on nonplanar structures as potential drug targets.^1^ In recent years there has been considerable interest in small bicyclic scaffolds because they orientate key pharmacophores in well-defined three-dimensional space.^2,3^ One such core structure is the 3-azabicyclo[3.1.0]hexane-6-carboxylate scaffold **1**. Several lead compounds and drug candidates have been developed incorporating this structural motif as illustrated with **2**–**7** (Scheme 1).^4−10^

**Scheme 1:**
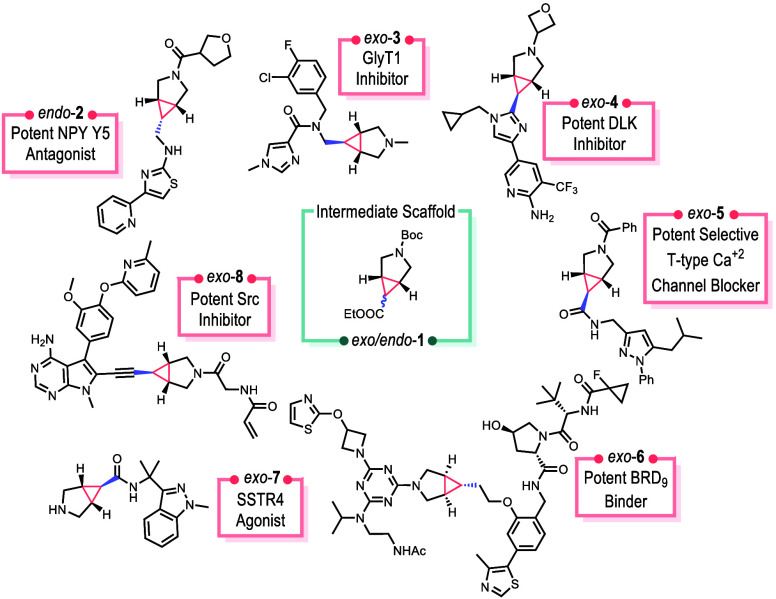
Pharmaceutical Relevance of 3-Azabicyclo[3.1.0]hexanes^4−10^

Considering the pharmaceutical significance of **1**, a plethora of methods^11^ have been developed for its synthesis but one obvious method, the cyclopropanation of 2,5-dihydropyrrole with ethyl diazoacetate (EDA), even though it has been explored by several groups,^4,6,8,10,12−16^ still needs considerable improvement (Scheme 2A). The published procedures require 1–7 mol % rhodium acetate as catalyst, and the resulting yields range from 8 to 66%. In recent years, great advances have been made in generating diazo compounds in flow,^17−24^ so the main remaining challenge is to improve the efficiency of the rhodium-catalyzed reaction. The Davies group has had a long-standing interest in the chemistry of donor/acceptor carbenes and has shown that cyclopropanation with these carbenes can be routinely conducted with very low catalyst loadings (<0.001 mol %).^25−28^ Therefore, we decided to embark on a collaborative study to determine whether the information gained about donor/acceptor carbenes could be applied to acceptor carbenes and, thus, achieve a practical entry to either diastereomer of the azabicyclic scaffold **1** (Scheme 2B). The results of this study are described herein.

**Scheme 2:**
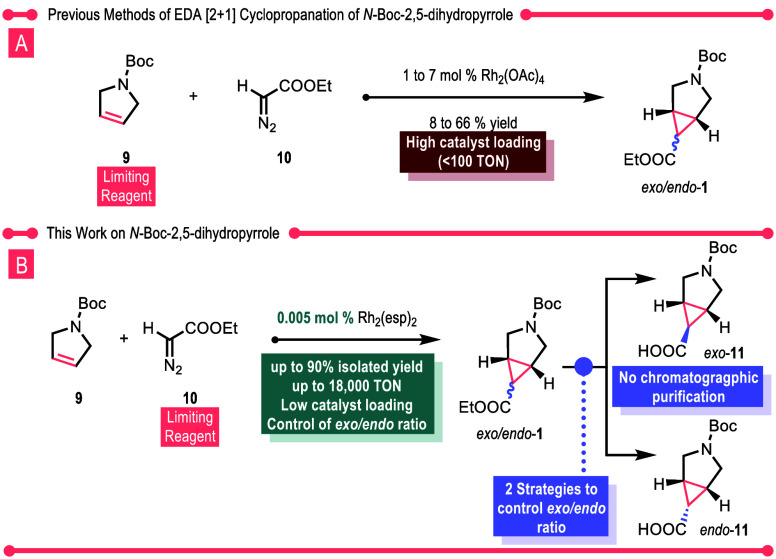
Previous Work [A] versus Current Work [B] on Dirhodium(II)-Catalyzed Cyclopropanation of *N*-Boc-2,5-Dihydropyrrole with EDA

The dirhodium(II)-catalyzed intermolecular cyclopropanation with donor/acceptor carbenes is generally far superior to the corresponding reaction with acceptor carbenes, such as the carbene derived from EDA. Highly diastereoselective and enantioselective reactions are routine (>20:1 d.r.),^26^ and we have reported that the turnover numbers (TONs) with donor/acceptor carbenes are vastly superior compared to acceptor carbenes.^27^ Fortunately, enantioselectivity is not an issue in the current study since *exo*/*endo*-**1** is meso. Furthermore, even if the diastereoselectivity is poor, equilibration could be possible under basic conditions to favor the thermodynamic *exo*-**1** diastereomer. Thus, the major question that needed to be addressed is whether the recent advances in catalyst design and the general understanding of rhodium–carbene chemistry could be applied to greatly increase the turnover efficiency of acceptor carbenes such that a commercially competitive process could be developed to access this pharmaceutically valuable scaffold. Our studies on high TON catalysis with donor/acceptor carbenes have revealed the following trends that are useful for the design of the current study: (1) An effective trapping of the carbene is required to achieve high TON, which means excess of trapping agent is beneficial.^27^ (2) Generally, trichloroethyl diazoacetate performs better than EDA.^26^ (3) Dimethyl carbonate (DMC) is more effective and environmentally friendly than dichloromethane.^26^ (4) Elevated temperatures enhance overall efficiency.^25^ (5) Bridged tetracarboxylate ligands confer greater stability to the catalysts.^28^

Using these trends as guiding principles, we conducted an optimization study for the cyclopropanation of *N*-Boc-2,5-dihydropyrrole **9**. The initial studies were conducted with EDA **10** using 1 mol % of the more commonly used achiral dirhodium(II) tetracarboxylate catalysts as well as the bridged dirhodium(II) catalyst Rh_2_(esp)_2_.^29^ In the previous studies, EDA **10** was used as the limiting agent^4,6,8,10,12−16^ because the dihydropyrrole **9** was relatively more expensive. As we wished to focus on enhancing the TON, we used an excess of the trap **9**, and furthermore, we used DMC as an environmentally benign replacement solvent to dichloromethane.^30^ Under these conditions, all the catalysts performed well giving *exo/endo*-**1** in 63–79% yield, although in most cases, the reaction gave a 1:1 mixture of the *exo* and *endo* diastereomers (Scheme 3). These results, in comparison to the previous literature studies,^4,6,8,10,12−16^ demonstrate that an excess of trapping agent would be advantageous when studying protocols for high TON transformations (see Table S1 for catalyst structures and complete optimization studies).

**Scheme 3:**
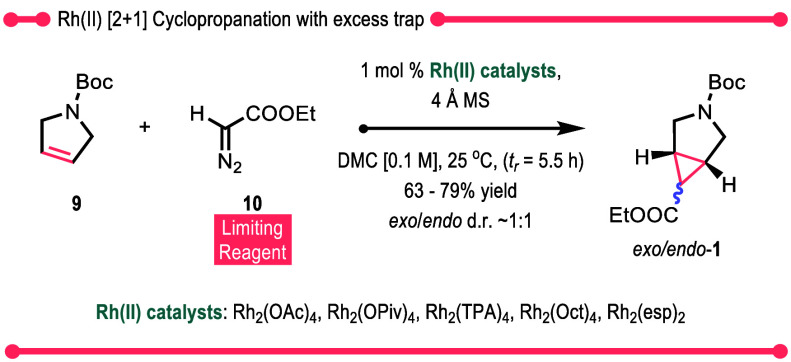
Cyclopropanation with Excess Trap

We then took on the more demanding challenge of conducting cyclopropanation with a much lower catalyst loading. We determined a catalyst loading of 0.005 mol % would be an appropriate target because, at such low catalyst loadings, the fluctuating cost of rhodium would not be especially impactful on the overall cost of the process (see Figure S13 for details). The results of the optimization study are summarized in Table 1. When the reaction was conducted at room temperature, *exo*/*endo*-**1** was produced in a low yield (entry 1). In the case of the donor/acceptor carbenes, we found that the optimum temperature for high TON was 60–70 °C.^25,26^ When the reactions with **10** were conducted at 70 °C, the yields of the cyclopropanation were still relatively low (9–32%) and the crude NMR showed evidence of unreacted EDA **10** (entries 2–5). The optimum catalyst in this study was the bridged tetracarboxylate catalyst Rh_2_(esp)_2_.

**Table 1:**
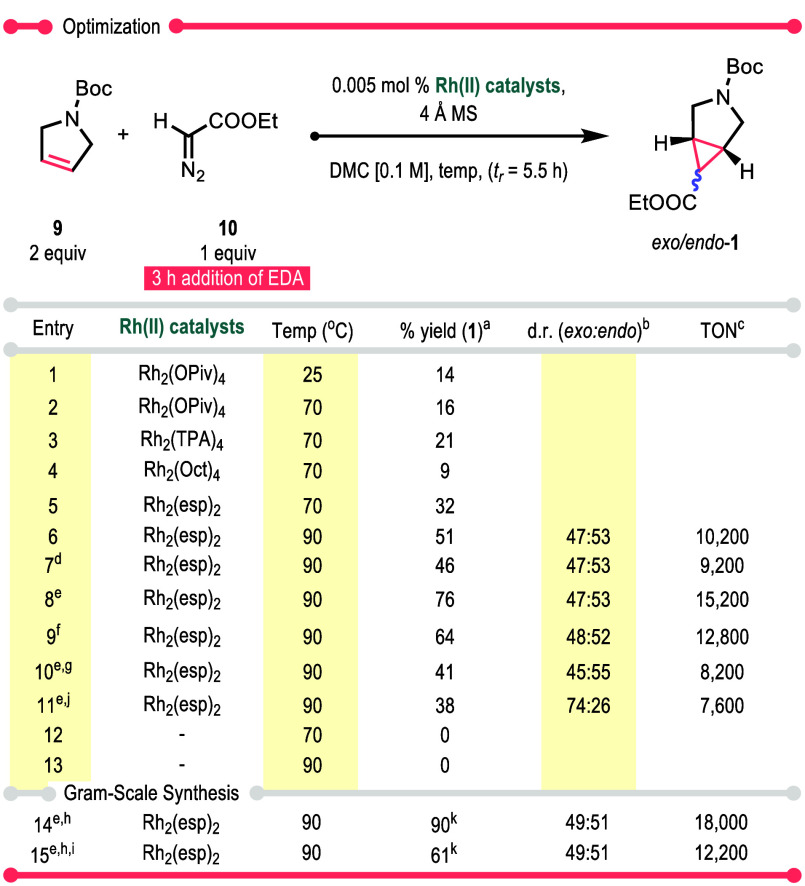
Optimization of Low Catalyst Loading and High TON with Achiral Dirhodium(II) Tetracarboxylates (Reactions Were Run at 0.500 mmol)

a qHNMR yield analysis with 1,3,5-trimethoxybenzene.

b d.r. was calculated from crude ^1^H NMR.

c For calculations, refer to the Supporting Information.

d 6 h slow addition (*t*_*r*_ = 8.5 h) instead.

e The total reaction concentration is 0.5 M.

f The total reaction concentration is 1 M.

g 4 Å molecular sieve is absent.

h 10 mmol reaction was performed.

i 15.7% EDA **10** in toluene was used instead of 74% EDA **10** in CH_2_Cl_2_.

j Trichloroethyl diazoacetate was used instead of EDA **10**.

k Isolated yield.

Further optimization of the Rh_2_(esp)_2_-catalyzed reaction was conducted at 90 °C, and under these conditions, the yield of *exo/endo*-**1** greatly improved to 76% (entry 8). In the case of aryldiazoacetates, the trihaloethyl esters often gave better performance,^26^ but with diazoacetates, this was not the case as the trichloroethyl derivative actually gave lower yield (entry 11). As these reaction temperatures are relatively high, control experiments were conducted in the absence of the catalysts (entries 12 and 13), which revealed that the products are not being formed under purely thermal conditions because EDA **10** remained unchanged. The reaction was scaled up to gram scale, and as is typical of this chemistry, the isolated yield was significantly improved in the larger scale reaction (90% isolated yield, entry 14). *Exo*/*endo*-**1** was cleanly isolated through Kugelrohr distillation, and no chromatographic purification was needed. The commercially available EDA **10**, dissolved in toluene, was also evaluated as the carbene source, but this was inferior (entry 15) because a reaction between the carbene and toluene competed with the desired cyclopropanation.

The next series of experiments were directed toward exploring whether the diastereoselectivity of the process could be directed toward the thermodynamically less favored *endo*-1 diastereomer (Table 2). As previously described, the achiral catalysts were relatively unselective, resulting in close to a 1:1 *exo*/*endo* mixture (Table 1). However, when the reaction was conducted using some of our recently developed chiral bowl-shaped catalysts, the reaction could be directed to favor the thermodynamically less favorable *endo* isomer (see Table S2 for details). Two of the most impressive catalysts in the study were Rh_2_(*S*-TPPTTL)_4_ and its brominated derivative, Rh_2_[*S*-*tetra*-(3,5-di-Br)TPPTTL]_4_. Both catalysts were effective at a catalyst loading of 0.005 mol %. Rh_2_(*S*-TPPTTL)_4_ gave 59% yield and a 24:75 *exo*/*endo* ratio, and Rh_2_[*S*-*tetra*-(3,5-di-Br)TPPTTL]_4_ gave a 70% yield and a 17:83 *exo*/*endo* ratio of **1**. Due to the promising results, the reaction catalyzed by Rh_2_[*S*-*tetra*-(3,5-di-Br)TPPTTL]_4_ was conducted on a gram scale resulting in the formation of *exo*/*endo*-**1** in 83% isolated yield with the same 17:83 *exo*/*endo* ratio.

**Table 2:**
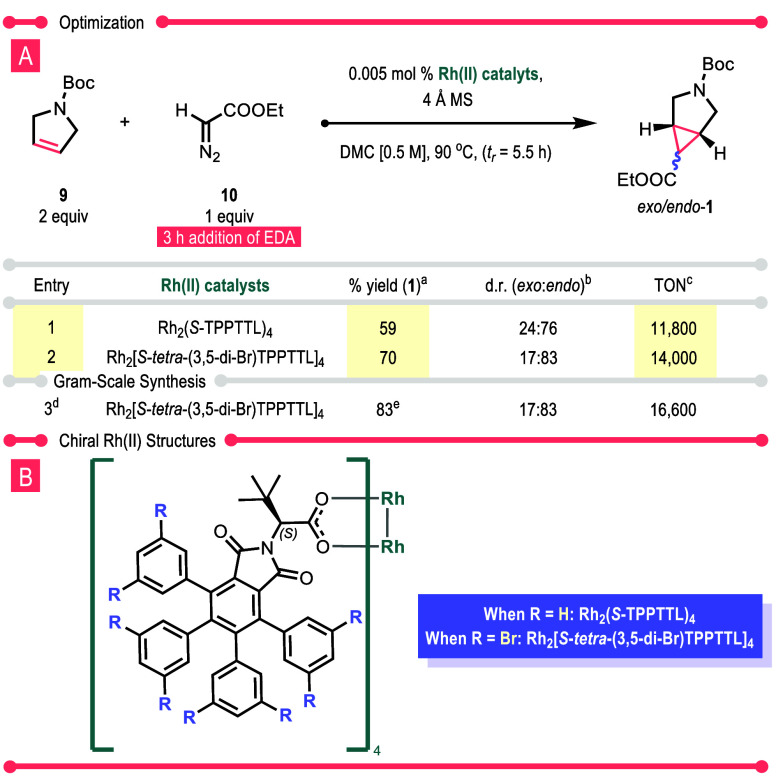
*Endo*-Selectivity with Chiral Dirhodium(II) Tetracarboxylates (Reactions Were Run at 0.500 mmol)

a qHNMR yield analysis with 1,3,5-trimethoxybenzene.

b d.r. was calculated from crude ^1^H NMR.

c For calculations, refer to the Supporting Information.

d Reaction ran at 10 mmol.

e Isolated yield.

The next series of experiments explored how to generate either the *exo* isomer or the *endo* isomer of **1** as a single diastereomer without resorting to any chromatographic purification (see Tables S3–S5 for detailed optimization studies). Treatment of a 1:1 mixture of *exo*/*endo*-**1** with sodium *tert*-butoxide caused epimerization at the α-carbonyl stereocenter of the ethyl ester to generate exclusively *exo*-**1**, which can be hydrolyzed with aqueous sodium hydroxide to form *exo*-**11** (Scheme 4A). *Exo*-**11** was isolated cleanly after an extraction protocol in 86% overall yield for the two steps in a one-pot procedure. Alternatively, *endo*-**11** could be obtained in pure form, starting from *exo*/*endo*-**1**, enriched in the *endo* isomer by a 17:83 *exo*/*endo* ratio by changing the reaction sequence. Treatment of *exo*/*endo*-**1** with aqueous sodium hydroxide resulted in the selective hydrolysis of *exo*-**1** to form carboxylate *exo*-**11**, and the resulting unreacted *endo*-**1** could be obtained cleanly as a single diastereomer after extractions. An extended exposure of *endo*-**1** to aqueous sodium hydroxide led to the clean formation of *endo*-**11** (Scheme 4B).

**Scheme 4:**
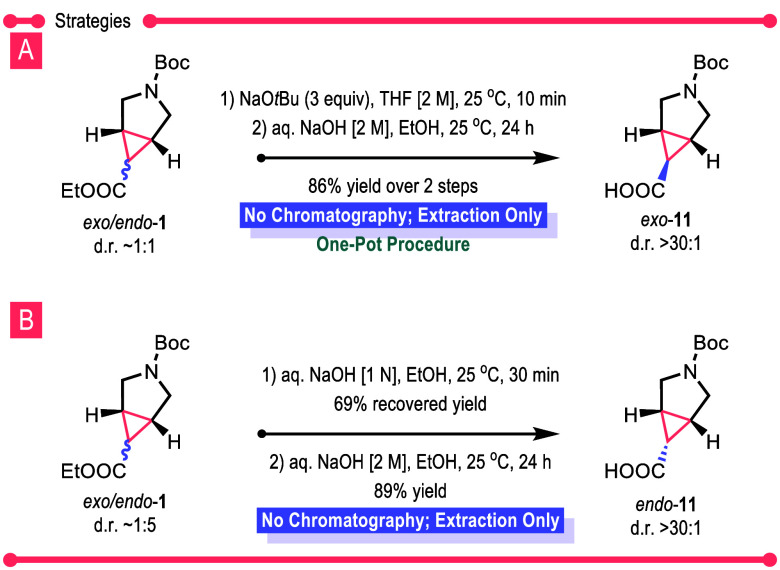
Strategy to Selectively Access *Exo*- and *Endo*-**11**

Having generated efficient procedures to generate either *exo*- or *endo*-**11**, we then explored the possibility of telescoping the reaction sequence so that all three steps can be combined (Scheme 5). The crude reaction from a 10 mmol scale rhodium-catalyzed cyclopropanation was filtered to remove the molecular sieves, and then the solvent was concentrated *in vacuo.* The crude material was then subjected to either the tandem isomerization-*exo*-hydrolysis conditions to afford the *exo*-**11** or the tandem selective hydrolysis followed by *endo*-hydrolysis to afford *endo*-**11**. Both telescoped sequences went very smoothly, generating either the *exo*-**11** in 76% combined yield or the *endo*-**11** in 54% combined yield, without the need of any chromatographic purification and Kugelrohr distillation. Due to the significance of either the *exo*- or *endo*-3-azabicyclo[3.1.0]hexanes, the major focus of this study has been achieved, which is the selective formation of either stereoisomer beginning with a cyclopropanation with low catalyst loadings. Other substrates that worked well in the telescoped route are *N*-tosyl-2,5-dihydropyrrole **12**, 2,5-dihydrofuran **13**, and cyclopentene **14** to afford the corresponding *exo*-acid in overall combined yields ranging from 58 to 72% (*exo*-**15**, **16**, and **17**). The *N*-tosyl and *N*-Boc-2,5-dihydropyrroles (**12** and **9**, respectively) can be recovered in these strategies, recycled, and reused.

**Scheme 5:**
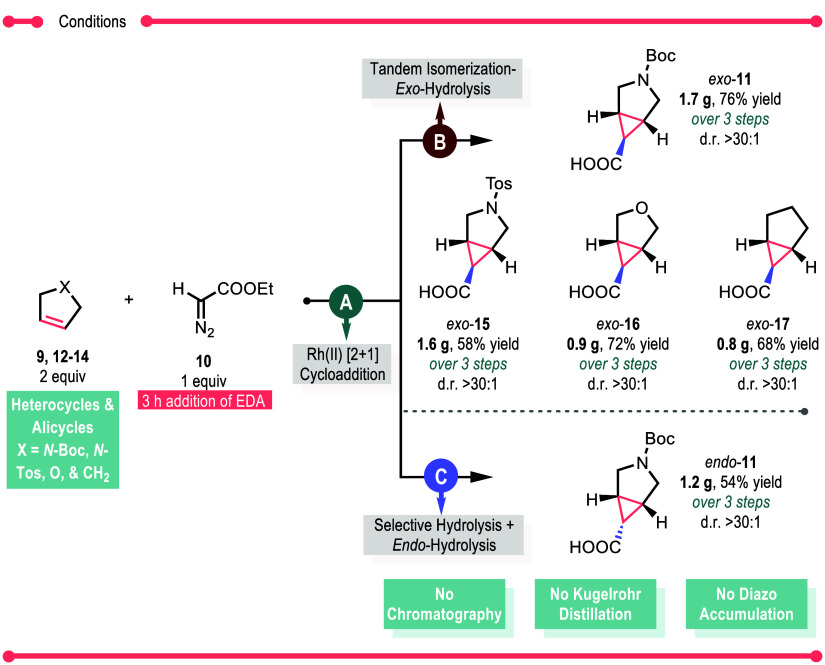
Telescoped Expansion of Trap Scope to Afford *Exo*- and *Endo*-Acids

All of these reactions were conducted on a 10 mmol scale to illustrate the scale-up potential of this chemistry. Furthermore, one of these reactions was followed by ReactIR and showed no accumulation of EDA **10** during the reaction (see Figures S6 and S7 for complete details).

In summary, these studies demonstrate that the cyclopropanation of 2,5-dihydropyrroles with EDA can be conducted with dirhodium(II) catalyst loadings as low as 0.005 mol %. These results illustrate that high turnover dirhodium(II) catalysis is not limited to donor/acceptor carbenes but can be extended to acceptor carbenes. Telescoped conditions were developed to enable the synthesis of either the *exo-* or *endo-*isomers of 3-azabicyclo[3.1.0]hexanes on a gram scale without requiring distillation or chromatographic purification, which demonstrates the practicality of the rhodium-catalyzed cyclopropanation for the synthesis of these valuable pharmaceutical intermediates.

## Data Availability

The data underlying this study are available in the published article and its Supporting Information.

## Supporting Information Available

Complete experimental procedures and compound characterization (PDF)
