# Supplementary material for: Stereoselective Synthesis of Either Exo- or Endo-3-Azabicyclo[3.1.0]hexane-6-carboxylates by Dirhodium(II)-Catalyzed Cyclopropanation with Ethyl Diazoacetate under Low Catalyst Loadings
Source: Org Lett. 2024 Jan 3;26(14):2832–6. doi: 10.1021/acs.orglett.3c03652 (PMC11020159; doi:10.1021/acs.orglett.3c03652)
Supplement: Supplementary file 1 — ol3c03652_si_001.pdf [file ol3c03652_si_001.pdf]

Stereoselective Synthesis of Either *Exo*- or *Endo*-3-Azabicyclo[3.1.0]hexane-6-carboxylates by Dirhodium(II)-Catalyzed Cyclopropanation with Ethyl Diazoacetate Under Low Catalyst Loadings

Terrence-Thang H. Nguyen[1], Antonio Navarro[2], J. Craig Ruble[2], Huw M. L. Davies[1]*

[1]Department of Chemistry, Emory University, Atlanta, Georgia 30322, United States

[2]Lilly Research Laboratories, Eli Lilly and Company, Indianapolis, Indiana 46285, United States

Supporting Information

Experimental procedures and characterization data for new compounds

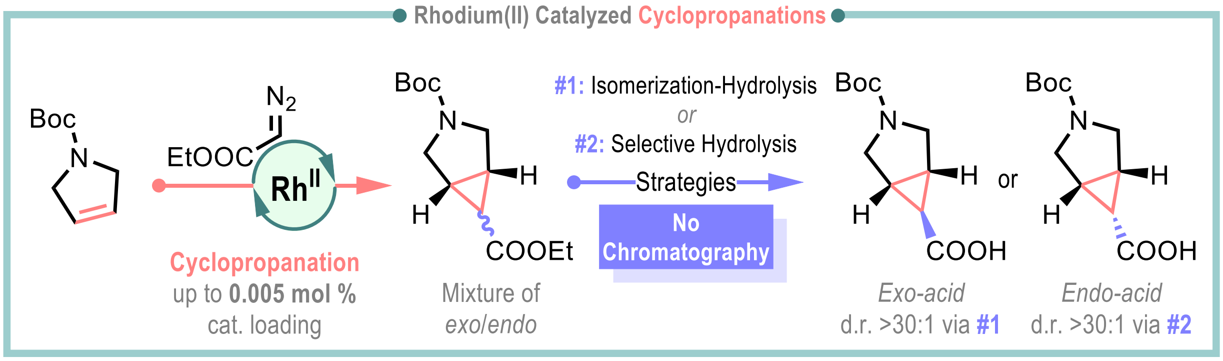

Table of Contents

1. General Considerations....2
2. Reagent and Substrate Acquisition....3
3. Catalyst Structures....6
4. Initial Achiral Catalyst Screen....14
5. Chiral Catalyst Screen for *Endo*-Selectivity....15
6. *Exo*/*Endo* Base Isomerization Optimization Study....15
7. Selective Hydrolysis Optimization....17
8. High TON, Low Catalyst Loading and Telescoped Procedures....20
9. ReactIR Experimentation – Diazo Accumulation Study....32
10. NMR Spectra – Synthesis of Rh_2[*S*-*tetra*-(3,5-di-Br)TPPTTL]$_4$....35
11. NMR Spectra – *Exo*- and *Endo*-Products....41
12. Crystal Structure Data and Experimental....48
13. Cost Analysis of Rhodium Catalyst Loading vs. *Exo*-**11**....65
14. References....65

1. General Considerations

Warning: This project involves the use of diazo compounds. Diazo compounds are known to have thermal stability issues and are explosive hazards; work with diazo compounds should be performed in a well-ventilated hood, require the use of PPE, and careful handling of the reagents.[1]

The absolute configuration is tentatively assigned by analogy based on X-ray crystallography. Reactions were carried out under nitrogen in flame-dried vessels unless otherwise specified. Heating was performed using a heating mantle, generally with an aluminum block, unless specified. 4 Å molecular sieves were activated by heating the sieves at 300 °C for over 3 hours under vacuum and then immediately storing the activated sieves in a 130 °C oven prior to use. Dimethylcarbonate (DMC) and dichloromethane were distilled from calcium hydride onto activated 4 Å molecular sieves, the distillate sparged with nitrogen for over 15 minutes, capped with a nitrogen balloon, and then stored under nitrogen for 24 h prior to use. Alkene traps that are liquids and used in [2 +1] cycloaddition were sparged with nitrogen for over 15 minutes before use. Syringe pumps utilized were acquired from KD Scientific. Flash column chromatography was performed using Biotage Isolera Purification Systems (default setting of KP Sil 100g) and on Silicycle SiliaFlash P60 silica gel (60 Å pore size, 40–63 µm particle size, 230–400 mesh) and ACS reagent grade solvents. The *exo*- and *endo*-products were confirmed with ^{1}H NMR to confirm quality of product and deemed that no chromatographic purification was needed. All ^{1}H NMR spectra were recorded at either 400 MHz, 500 MHz, or 600 MHz on Varian/Bruker-400, Varian-500, or Varian/Bruker-600 spectrometers. ^{13}C NMR spectra were recorded at either 101 MHz, 126 MHz, or 151 MHz on Varian/Bruker-400, Varian-500, or Bruker 600 spectrometers. NMR spectra were obtained from solutions of deuterated chloroform ($CDCl_3$) with or without 0.03% TMS with residual solvent serving as internal standard (7.26 ppm for ^{1}H or 0.00 ppm and 77.16 ppm for ^{13}C). Mestrelab Research Chemistry Software Solutions - Mnova was used to analyze NMR spectra. All

NMR spectra were processed via the processing function of the Mnova software by "Automatic phase correction" and then "Automatic baseline correction" of the full spectrum before analysis. NMR shifts were reported in parts per million (d ppm). Abbreviations for signal multiplicity are as follow: s = singlet, d = doublet, t = triplet, q = quartet, m = multiplet, brs = broad singlet, dd = doublet of doublet, etc. Coupling constants (J values) were calculated directly from the spectra. All reagents were purchased commercially and used without further purification unless otherwise noted. Optical rotations were measured on Rudolph Research Analytical Automatic Polarimeter APIV-1W. Mass spectra were taken on a Thermo Finnigan LTQ-FTMS spectrometer with APCI, ESI or NSI. ReactIR experiments were conducted on Mettler Toledo ReactIR 45m and using iC IR 4.3 software for data acquisition and processing. Temperature was recorded via heat-block and not internal temperature of reaction. Turnover number (TON) was calculated by [(1 equiv of limiting reagent / x equiv of catalyst)] x [(% yield / 100)]. Melting points were measured in open capillary tubes with a Mel-Temp Electrothermal melting point apparatus and are uncorrected.

2. Reagent and Substrate Acquisition

Reagent	CAS #	Company(s)	Notes
tert-Butyl 2,5-dihydro-1H-pyrrole-1-carboxylate (*N*-Boc-2,5-dihyropyrrole or *N*-Boc-3-pyrroline) (**9**)	73286-70-1	Ambeed, TCI Chemicals, Combi-Blocks	Impurities (mainly *N*-Boc pyrrole) present in all samples; **repurified before use**. Physical characteristic as a white crystalline solid.
N-(P-Toluenesulfonyl)-3-pyrroline (**12**)	16851-72-2	Combi-Blocks	
2,5-Dihydrofuran (**13**)	1708-29-8	Combi-Blocks	
Cyclopentene (**14**)	142-29-0	Oakwood Chemicals	
Dimethyl Carbonate (DMC)	616-38-6	Oakwood Chemicals	Distilled with CaH using a short-path distillation into activated 4 Å MS. Dried solution is sparged with nitrogen over 15 minutes and capped with nitrogen balloon.
CH_2Cl_2, suitable for HPLC, >99.8%, contains amylene as stabilizer	75-09-2	Sigma-Aldrich	Distilled with CaH using a short-path distillation into activated 4 Å MS. Dried solution is sparged with nitrogen over 15 minutes and capped with nitrogen balloon.
Sodium *tert*-butoxide solution, 2 M in THF, ZerO$_2$ (Degassed and low oxygen)	865-48-5	Sigma-Aldrich	Used fresh for each isomerization.
Potassium *tert*-butoxide solution in 1 M THF	865-47-4	Sigma-Aldrich	Used fresh for each isomerization.
Ethyl diazoacetate solution 15% in toluene	623-73-4	Sigma-Aldrich	15.7% EDA in toluene based on COO/COA.
Ethyl diazoacetate contains $\geq$ 13 wt. % CH_2Cl_2	623-73-4	Sigma-Aldrich	^{1}H NMR analysis was performed before use to determine actual wt. % of EDA in CH_2Cl_2;

			determined to be 74% wt. of EDA in CH_2Cl_2.
$Rh_2(esp)_2$	1708-29-8	Sigma-Aldrich	
1,3,5-Trimethoxybenzene	621-23-8	Sigma-Aldrich	*ReagentPlus*, >99%

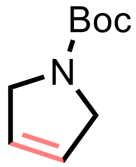

***tert*-butyl 2,5-dihydro-1H-pyrrole-1-carboxylate (9)**

The material (generally a brown oil or liquid containing *N*-Boc-2,5-dihyropyrrole (**9**), *N*-Boc-pyrrole, and other byproducts) was purchased and repurified before use via Kugelrohr distillation at 130 °C under vacuum at 4 x 10^{-1} torr. This separated out the product, which collected in the collecting bulbs, from the black oil as the pot residue. The product (**9**) was distilled with *N*-Boc-pyrrole. Subsequently, a flash chromatography run was performed to separate product (**9**) from *N*-Boc-pyrrole. Approximately between 50 – 100 g of *N*-Boc-2,5-dihyropyrrole (**9**) was dry-loaded onto SiO_2 (make sure the dry-load is a free-flowing powder; best separation is observed with very dry dry-load) and subjected to SiO_2 flash chromatography. The parameters set for the purification is as follows:

Column Parameter: KP Sil 100 g setting

Solvent System: Hexanes/diethyl ether

Column: 300 g Biotage DLV Sfär column

Flowrate: 150 mL/min

Detector: 210/254 nm

Gradient: 0% diethyl ether 15 CVs to 10% diethyl ether 15 CVs

TLC: 10% ethyl acetate/90% *n*-heptane solvent system; *N*-Boc pyrrole (Rf 0.8) and *N*-Boc-2,5-dihyropyrrole (**9**) (Rf 0.6); *N*-Boc pyrrole is $KMnO_4$ inactive but is UV-active at 254 nm; *N*-Boc-2,5-dihyropyrrole (**9**) is UV-inactive at 254 nm but is $KMnO_4$ active appearing as a yellow spot (without heat).

The product (**9**) eluted as non-colored fractions and detection of only 210 nm (no detection at 254 nm). Byproducts had detection at both 210 nm and 254 nm. The byproduct eluted first and then product elutes next, with clean separation. The collected fractions were concentrated *in vacuo* into a vessel, the vessel was backfilled with nitrogen three times and sealed, and then stored in a -20 °C freezer. The resulting physical characteristic of *N*-Boc-2,5-dihyropyrrole (**9**) is a white crystalline solid, handled as a solid at room temperature, and the ^1H NMR spectrum is in good agreement with literature precedent.[2]

^1H NMR (400 MHz, $CDCl_3$) δ 5.85 – 5.48 (m, 2H), 4.03 (ddt, J = 17.7, 5.4, 2.6 Hz, 4H), 1.40 (s, 9H).

Melting Point (°C): 35 – 37 °C.

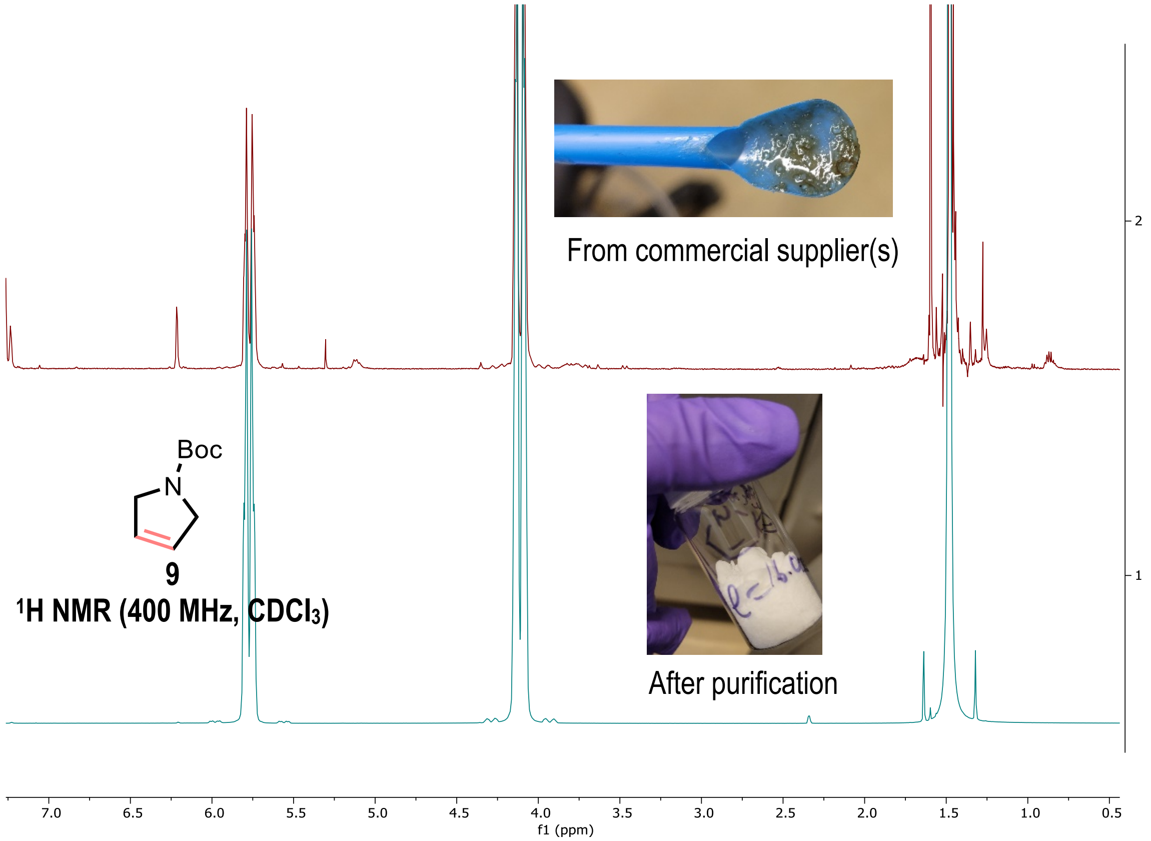
From commercial supplier(s)
After purification
Boc
N
9
1H NMR (400 MHz, CDCl3)
f1 (ppm)

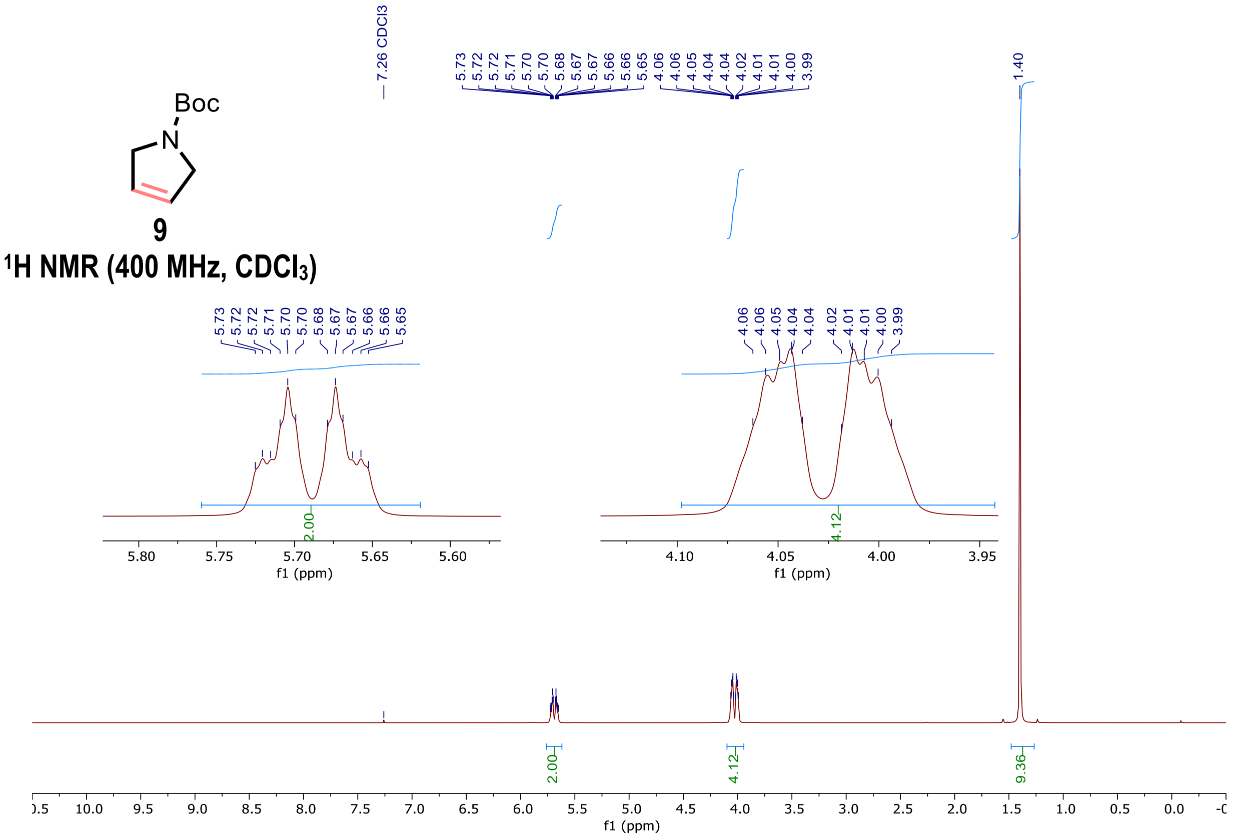
7.26 CDCl3
5.73 5.72 5.72 5.71 5.70 5.70 5.68 5.67 5.67 5.66 5.66 5.65
4.06 4.06 4.05 4.04 4.04 4.02 4.01 4.01 4.00 3.99
1.40
Boc
N
9
1H NMR (400 MHz, CDCl3)
2.00
4.12
9.36
f1 (ppm)

3. Catalyst Structures

The following chiral catalysts were used in this study and have been prepared previously.

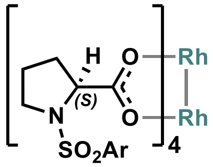<br>Ar = *p*-($C_{12}H_{25}$)C_6H_4<br>**Rh_2(*S*-DOSP)$_4$**	**Generation:** Prolinato<br>**Name:** Rh_2(*S*-DOSP)$_4$[3]
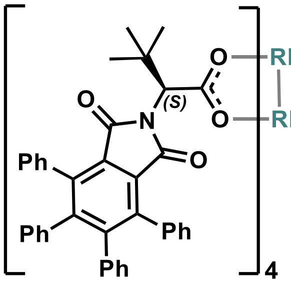<br>**Rh_2(*S*-TPPTTL)$_4$**	**Generation:** Phthalimido<br>**Name:** Rh_2(*S*-TPPTTL)$_4$[4]
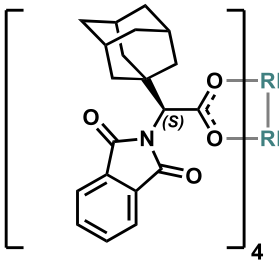<br>**Rh_2(*S*-PTAD)$_4$**	**Generation:** Phthalimido<br>**Name:** Rh_2(*S*-PTAD)$_4$[5]
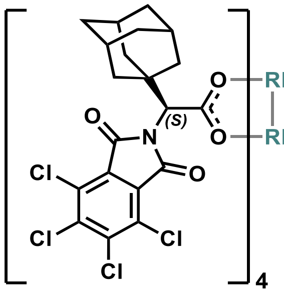<br>**Rh_2(*S*-TCPTAD)$_4$**	**Generation:** Phthalimido<br>**Name:** Rh_2(*S*-TCPTAD)$_4$[5]
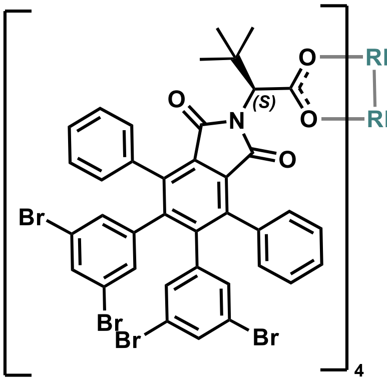<br>**Rh_2[*S*-*di*-(3,5-diBr)TPPTTL]$_4$**	**Generation:** Phthalimido<br>**Name:** Rh_2[*S*-*di*-(3,5-diBr)TPPTTL]$_4$[6]

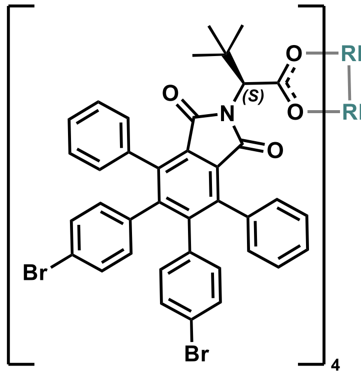<br>Rh_2(*S-di*-(4-Br)TPPTTL)$_4$	**Generation:** Phthalimido<br>**Name:** Rh_2(*S-di*-(4-Br)TPPTTL)$_4$[6]
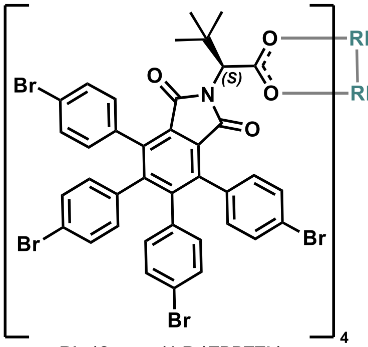<br>Rh_2(*S-tetra*-(4-Br)TPPTTL)$_4$	**Generation:** Phthalimido<br>**Name:** Rh_2(*S-tetra*-(4-Br)TPPTTL)$_4$[6]
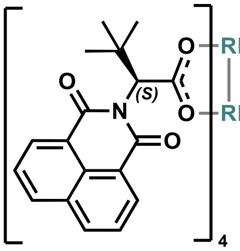<br>Rh_2(*S*-NTTL)$_4$	**Generation:** Napthalimido<br>**Name:** Rh_2(*S*-NTTL)$_4$[7]
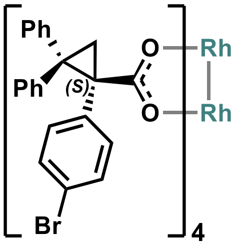<br>Rh_2(*S-p*-BrTPCP)$_4$	**Generation:** TPCP<br>**Name:** Rh_2(*S-p*-BrTPCP)$_4$[8]
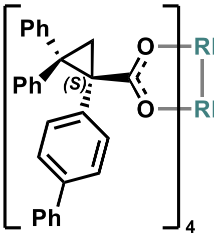<br>Rh_2(*S-p*-PhTPCP)$_4$	**Generation:** TPCP<br>**Name:** Rh_2(*S-p*-PhTPCP)$_4$[8]

Figure S1. Chiral dirhodium(II) tetracarboxylate catalysts used in catalyst screen that have previously been reported.

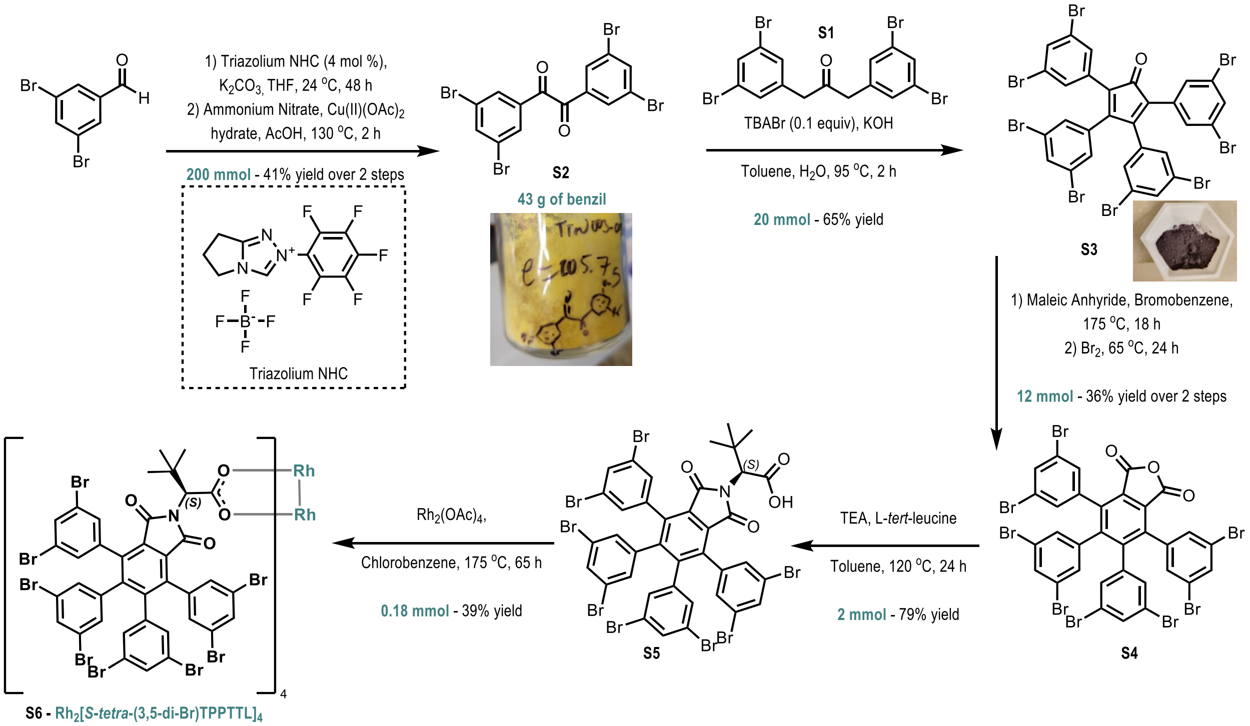

Figure S2. Synthetic route to access novel Rh_2[*S*-*tetra*-(3,5-di-Br)TPPTTL]$_4$. Triazolium NHC was synthesized via OrgSyn procedure (Org. Synth. 2010, 87, 350).

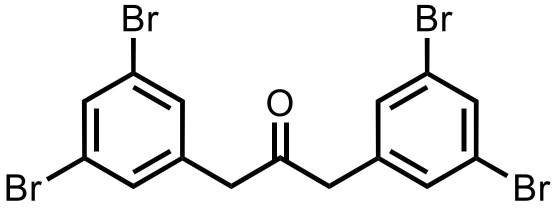

1,3-bis(3,5-dibromophenyl)propan-2-one (S1)

From a modified procedure from Li (2017) *et al.*[9]: To a 2 L erlenmeyer flask charged with a stir-bar was added 1,3-dibromo-5-(bromomethyl)benzene (23 g, 2 equiv, 70 mmol), tetrabutylammonium bromide (4.5 g, 0.4 equiv, 14 mmol), tosylmethyl isocyanide (6.8 g, 1 equiv, 35 mmol), and CH_2Cl_2 (3.0 g, 0.70 L, 0.050 molar, 1 equiv, 35 mmol). The reaction mixture was allowed to stir and subsequently, sodium hydroxide in deionized water (64 g, 0.32 L, 5 molar, 46 equiv, 1.6 mol) was added to the mixture. The reaction mixture was allowed to stir for 24 h at 25 °C open to air. After the elapsed time, deionized water was poured into the reaction solution and allowed to stir for 5 minutes. The aqueous layer was decanted into a waste container. This wash technique was repeated three times. Afterwards, the organic layer was concentrated via a stream of air.

Once the crude material had dried, 233 mL of CH_2Cl_2, 46 mL of THF, and 23 mL of concentrated 12 M HCl was added to the crude material. The mixture was allowed to stir at room temperature for 20 h. The reaction mixture was then neutralized with saturated bicarbonate solution while the solution stirred. Subsequently, a stream of air was placed in the vessel to evaporate the organic solvents, leaving just the aqueous suspension. The suspension was filtered with a Büchner funnel charged with a paper filter, the

filter cake washed with deionized water, and then triturated with excess ethanol to afford a white filter cake. The white filter cake was vacuum dried to afford 1,3-bis(3,5-dibromophenyl)propan-2-one **(S1)** (15.95 g, 30.33 mmol, 87% yield) as a white powder. NMR data is in good agreement with literature precedent.[9]

^{1}H NMR (400 MHz, CDCl$_3$) δ 7.60 – 7.59 (t, J = 1.8 Hz, 2H), 7.24 – 7.23 (d, J = 1.7 Hz, 4H), 3.69 (s, 4H).

^{13}C NMR (101 MHz, CDCl$_3$) δ 202.4, 136.9, 133.1, 131.3, 123.2, 48.3.

HRMS (+p APCI): calc. mass for $C_{15}H_{11}O^{79}Br_4$ [M + H]$^+$ - 522.7538; obs. mass for $C_{15}H_{11}O^{79}Br_4$ [M + H]$^+$ - 522.7529.

Melting Point (°C): 185 – 187 °C.

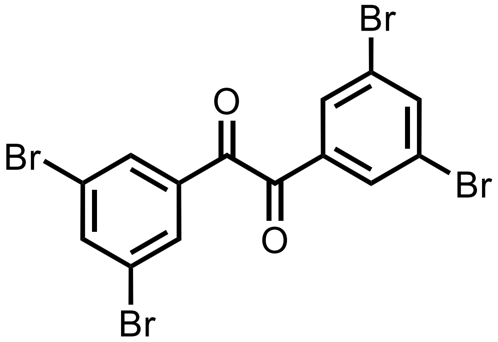

1,2-bis(3,5-dibromophenyl)ethane-1,2-dione (S2)

From a modified procedure from Dhayalan (2019) *et al.*[10]: To a flame-dried 250 mL round-bottom flask charged with a stir-bar was added 2-(perfluorophenyl)-6,7-dihydro-5H-pyrrolo[2,1-c][1,2,4]triazol-2-ium tetrafluoroborate (2.90 g, 0.04 equiv, 8.00 mmol) and potassium carbonate (1.11 g, 0.04 equiv, 8.00 mmol) in THF (14.4 g, 200 mL, 1 molar, 1 equiv, 200 mmol). The mixture was stirred for at least 5 minutes at room temperature; the resulting solution is a red-orange suspension. To the reaction mixture was added 3,5-dibromobenzaldehyde (52.8 g, 1 equiv, 200 mmol). The solution was stirred overnight for 24 h at room temperature. After the elapsed time, the reaction was concentrated *in vacuo* at 40 °C. The crude was subjected to the next step as a crude orange residue.

From a modified procedure from Garlets (2022) *et al.*[6]: To a 1 L round-bottom flask charged with a stir-bar was added the crude benzoin, ammonium nitrate (20.0 g, 1.25 equiv, 250 mmol), and copper(II) acetate monohydrate (5.03 g, 0.126 equiv, 25.2 mmol). Subsequently, acetic acid (800 mL) was added to the vessel. The reaction vessel was then equipped with a findenser and was stirred at 130 °C for 24 h open to air. After the elapsed time, ice was added to the reaction vessel to aid in the cooling of the reaction solution and precipitation of product. Enough ice was added to the mixture until the reaction solution is cooled and ice can be seen floating atop the reaction solution. This resulting in a green-yellow suspension. The precipitate was vacuum filtered through a fritted funnel, washed with excess deionized water and then excess ice/ethanol mixture until the filtrate ran clear and colorless. Room temperature ethanol was used to further wash the filter cake until eluent ran clear and colorless. The bright yellow filter cake was vacuum dried to afford 1,2-bis(3,5-dibromophenyl)ethane-1,2-dione **(S2)** (43 g, 82 mmol, 41% yield *over 2 steps*) as a bright yellow powder. NMR data is in good agreement with literature precedent.[6]

^{1}H NMR (400 MHz, CDCl$_3$) δ 8.03 – 8.02 (d, J = 1.8 Hz, 4H), 7.98 – 7.97 (t, J = 1.8 Hz, 2H).

^{13}C NMR (101 MHz, CDCl$_3$) δ 189.4, 140.4, 135.1, 131.6, 124.0.

HRMS (+p APCI): calc. mass for $C_{14}H_7O_2{}^{79}Br_4$ [M + H]+ - 522.7174; obs. mass for $C_{14}H_7O_2{}^{79}Br_4$ [M + H]+ - 522.7176.

Melting Point (°C): 194 – 196 °C.

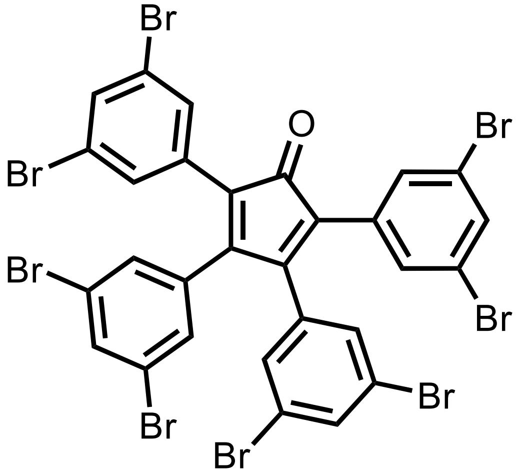

2,3,4,5-tetrakis(3,5-dibromophenyl)cyclopenta-2,4-dien-1-one (S3)

From a modified procedure of Li (2017) *et al.*[9]: To a 250 mL round-bottom flask charged with a stir-bar was added 1,2-bis(3,5-dibromophenyl)ethane-1,2-dione **(S2)** (10.62 g, 1 equiv, 20.20 mmol), tetrabutylammonium bromide (716.3 mg, 0.11 equiv, 2.222 mmol), and 1,3-bis(3,5-dibromophenyl)propan-2-one **(S1)** (10.62 g, 1 equiv, 20.20 mmol) in toluene (166 mL) and deionized water (11.9 mL). The reaction mixture was heated to 95 °C while stirring open to air. Once the temperature was reached, pellets of potassium hydroxide (1.667 g, 85% wt, 1.25 equiv, 25.25 mmol) were slowly added to the mixture. The resulting solution turned from a dark yellow suspension to a dark-red homogenous solution. The reaction was allowed to stir at 95 °C for 2 h. After the elapsed time, the resulting mixture was concentrated *in vacuo* at 80 °C to afford 2,3,4,5-tetrakis(3,5-dibromophenyl)cyclopenta-2,4-dien-1-one **(S3)** as a purple amorphous solid. The dried purple amorphous solid was resuspended in ethanol, sonicated, and then filtered through a Büchner funnel charged with a filter paper and washed with excess ethanol to afford 2,3,4,5-tetrakis(3,5-dibromophenyl)cyclopenta-2,4-dien-1-one **(S3)** (13.28 g, 13.08 mmol, 64.73% yield) as a purple powder. NMR data is in good agreement with literature precedent.[9]

^{1}H NMR (400 MHz, $CDCl_3$) δ 7.71 – 7.70 (t, J = 1.8 Hz, 2H), 7.63 – 7.62 (t, J = 1.8 Hz, 2H), 7.30 (d, J = 1.7 Hz, 4H), 7.00 (d, J = 1.8 Hz, 4H).

^{13}C NMR (101 MHz, $CDCl_3$) δ 196.6, 152.1, 135.4, 134.4, 134.2, 132.2, 131.4, 130.5, 124.2, 123.3, 122.9.

HRMS (+p APCI): calc. mass for $C_{29}H_{13}O^{79}Br_8$ [M + H]+ - 1008.4428; obs. mass for $C_{29}H_{13}O^{79}Br_8$ [M + H]+ - 1008.4439.

Melting Point (°C): >210 °C.

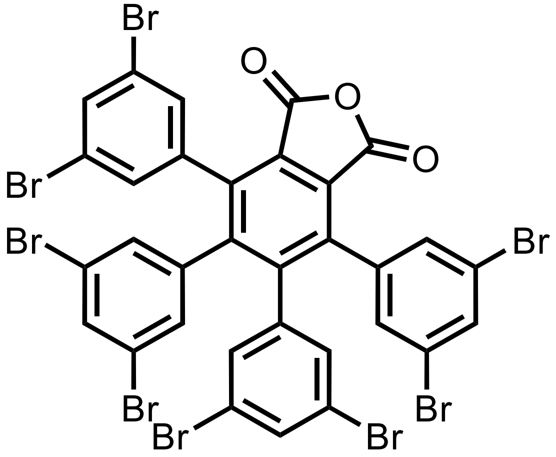

4,5,6,7-tetrakis(3,5-dibromophenyl)isobenzofuran-1,3-dione (S4)

From a modified procedure from Garlets (2022) *et al.*[6]: To a 2-neck flame-dried 100 mL round-bottom flask charged with a stir-bar was added 2,3,4,5-tetrakis(3,5-dibromophenyl)cyclopenta-2,4-dien-1-one **(S3)** (12.19 g, 1 equiv, 12.00 mmol), maleic anhydride (2.353 g, 2 equiv, 24.00 mmol), and bromobenzene (48.00 mL). The reaction vessel was equipped with a findenser and the purple suspension was heated to 175 °C while stirring for 18 h. After the elapsed time, the reaction solution is brown-purple suspension. A stream of air was blown into the reaction vessel to cool the solution to room temperature, which resulted in a brown-purple suspension. Then bromine (4.794 g, 1.546 mL, 2.5 equiv, 30.00 mmol) was added drop-wise into the reaction vessel. The vessel was heated to 65 °C for 24 h with an outlet-tube connected to the top of the findenser into a beaker of 1 M sodium thiosulfate solution. After the elapsed time, the reaction solution was allowed to cool to room temperature by blowing a stream of air into the unused flask neck, which resulted in a light brown suspension. The reaction solution was then quenched with 1 M sodium thiosulfate solution. To this suspension was diluted with deionized water. To the aqueous solution was diluted with ethyl acetate and extracted with ethyl acetate three times. The organic layer was dried with $MgSO_4$, filtered, and the filtrate concentrated *in vacuo* at 70 °C to afford a purple-red solid. The purple-red solid was resuspended in diethyl ether, sonicated, and filtered through a fritted funnel under vacuum. The filter cake was collected and vacuum dried to afford 4,5,6,7-tetrakis(3,5-dibromophenyl)isobenzofuran-1,3-dione **(S4)** (4.7 g, 4.3 mmol, 36% yield) as a cream solid.

^{1}H NMR (400 MHz, $CDCl_3$) δ 7.66 – 7.65 (t, J = 1.7 Hz, 2H), 7.45 – 7.44 (t, J = 1.7 Hz, 2H), 7.21 – 7.20 (d, J = 1.7 Hz, 4H), 6.93 (d, J = 1.7 Hz, 4H).

^{13}C NMR (101 MHz, $CDCl_3$) δ 160.1, 146.9, 138.6, 138.5, 135.9, 134.6, 133.9, 131.6, 131.0, 128.6, 122.8, 122.7.

HRMS (+p APCI): calc. mass for $C_{32}H_{13}O_3{}^{79}Br_8$ $[M + H]^+$ - 1076.4326; obs. mass for $C_{32}H_{13}O_3{}^{79}Br_8$ $[M + H]^+$ - 1076.4338.

Melting Point (°C): >210 °C.

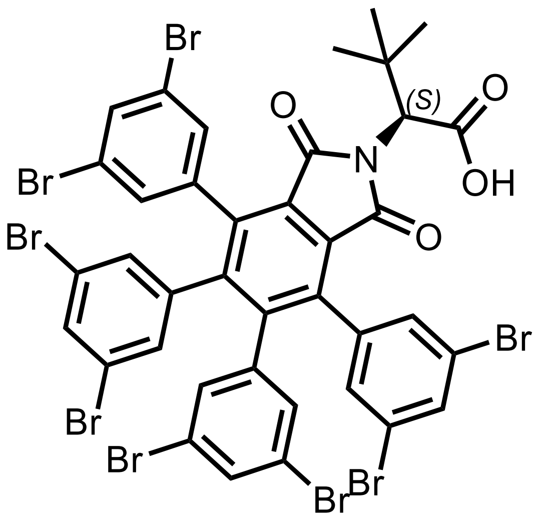

(*S*)-3,3-dimethyl-2-(4,5,6,7-tetrakis(3,5-dibromophenyl)-1,3-dioxoisoindolin-2-yl)butanoic acid (S5)

From a modified procedure of Garlets (2022) *et al.*[6]: To a flame-dried 50 mL round-bottom flask charged with a stir-bar was added 4,5,6,7-tetrakis(3,5-dibromophenyl)isobenzofuran-1,3-dione **(S4)** (2.17 g, 1 equiv, 2.00 mmol), 3-methyl-L-valin (*L-tert*-Leucine) (315 mg, 1.2 equiv, 2.40 mmol), and toluene (184 mg, 20.0 mL, 0.1 molar, 1 equiv, 2.00 mmol). Subsequently, triethylamine (263 mg, 362 µL, 1.3 equiv, 2.60 mmol) was added to the solution. The resulting solution was a brown suspension. The reaction vessel was equipped with a findenser open to air and the reaction suspension was heated to 120 °C for 24 h. The resulting solution is a clear brown solution. The reaction solution was concentrated *in vacuo* at 50 °C, the dried material diluted with 1 M HCl solution, sonicated vigorously, and then diluted with ethyl acetate. The organic layer was then extracted with ethyl acetate three times, the organic layers combined and dried with $MgSO_4$, filtered, and the filtrate concentrated *in vacuo* at 40 °C. The crude material was dry-loaded on SiO_2 and subjected to SiO_2 flash chromatography (hexanes/ether solvent system; 50 g Biotage DLV Sfär column; 0% ether for 3 CV → 0 to 25% ether for 3 CV → 25% ether for 3 CV → 25 to 75% ether for 3 CV → 75% ether for 3 CV → 75% to 100% ether for 3 CV → 100% ether for 5 CV; 100 mL/min flowrate; 210/254 nm detector; product elutes at 25% ether). The collected fractions were concentrated *in vacuo* to afford (*S*)-3,3-dimethyl-2-(4,5,6,7-tetrakis(3,5-dibromophenyl)-1,3-dioxoisoindolin-2-yl)butanoic acid **(S5)** (1.88 g, 1.57 mmol, 78.5% yield) as a cream powder.

^{1}H NMR (400 MHz, $CDCl_3$) δ 7.61 – 7.60 (t, J = 1.7 Hz, 2H), 7.41 – 7.40 (t, J = 1.7 Hz, 2H), 7.25 (s, 2H), 7.15 (s, 2H), 6.92 – 6.91 (d, J = 1.7 Hz, 4H), 4.67 (s, 1H), 1.15 (s, 9H).

^{13}C NMR (101 MHz, $CDCl_3$) δ 165.5, 145.1, 139.2, 137.1, 136.9, 134.0, 133.5, 131.8, 131.8, 131.3, 131.2, 128.6, 122.4, 122.4, 60.1, 35.7, 28.0.

HRMS (+p APCI): calc. mass for $C_{38}H_{24}O_4N^{79}Br_8$ $[M + H]^+$ - 1189.5167; obs. mass for $C_{38}H_{24}O_4N^{79}Br_8$ $[M + H]^+$ - 1189.5189.

$[\alpha]^{20}_D$: -34.0° (c 0.488 g/100 mL, EtOAc).

Melting Point (°C): >210 °C.

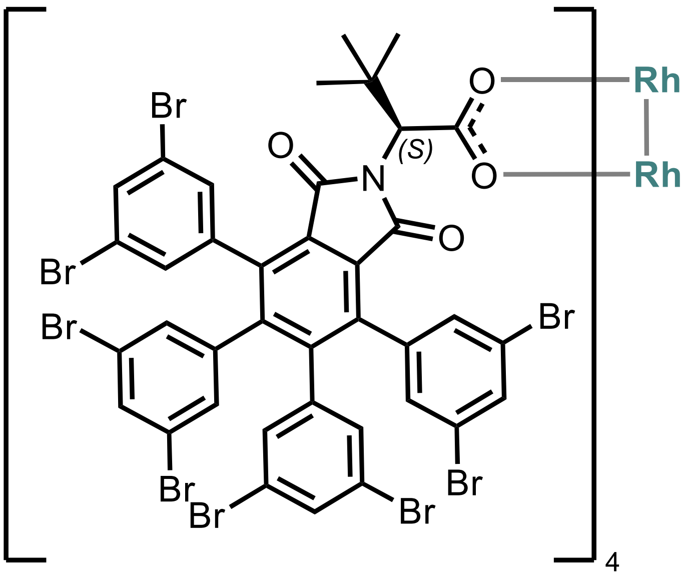

Rh_2[*S-tetra*-(3,5-di-Br)TPPTTL]$_4$ (S6)

From a modified procedure from Garlets (2022) *et al.*[6]: To a flame-dried 50 mL round-bottom flask charged with a stir-bar was added $Rh_2(OAc)_4$ (80 mg, 1 equiv, 0.18 mmol), (*S*)-3,3-dimethyl-2-(4,5,6,7-tetrakis(3,5-dibromophenyl)-1,3-dioxoisoindolin-2-yl)butanoic acid **(S5)** (1.7 g, 8 equiv, 1.4 mmol), and chlorobenzene (20 mg, 18 mL, 0.01 molar, 1 equiv, 0.18 mmol). The 50 mL round-bottom flask was equipped with a soxhlet apparatus charged with potassium carbonate and topped with a findenser and nitrogen balloon. The mixture was vigorously stirred and heated to 175 °C for 65 hour. The chlorobenzene was distilled off and then the crude solid was dry-loaded onto SiO_2 as a fine-green powder and subjected to SiO_2 flash chromatography (hexanes/ether solvent system; 25 g Biotage DLV Sfär column; 0% ether for 5 CV → 0 to 5% ether for 5 CV → 5% ether for 5 CV → 5% to 10% ether 5 CV → 10% ether for 5 CV; 100 mL/min flowrate; 210/254 nm detector; product elutes at 5% ether). The collected fractions were concentrated *in vacuo* at 60 °C to afford Rh_2[*S-tetra*-(3,5-di-Br)TPPTTL]$_4$ **(S6)** (350 mg, 70.2 µmol, 39% yield) as a green powder.

Crystallization - the solids were dissolved in $CDCl_3$, the solution placed into a culture tube, the solution was layered with benzene on top. The culture tube was placed into a scintillation tube and allowed to crystallize out through slow evaporation. Crystals became apparent within a couple of hours.

^{1}H NMR (400 MHz, $CDCl_3$) δ 7.66 – 7.65 (t, J = 1.8 Hz, 1H), 7.41 – 7.37 (m, 4H), 7.24 – 7.23 (t, J = 1.7 Hz, 1H), 7.04 – 7.03 (d, J = 1.6 Hz, 1H), 6.96 – 6.93 (dt, J = 9.2, 1.6 Hz, 2H), 6.80 (s, 1H), 6.75 – 6.74 (t, J = 1.6 Hz, 1H), 6.61 – 6.60 (t, J = 1.6 Hz, 1H), 3.83 (s, 1H), 0.77 (s, 9H).

^{13}C NMR (101 MHz, $CDCl_3$) δ 186.6, 164.9, 164.5, 144.8, 144.4, 139.6, 139.6, 137.7, 136.9, 136.5, 136.3, 133.8, 133.7, 133.3, 132.4, 132.0, 131.7, 131.3, 131.2, 129.0, 128.9, 123.0, 122.4, 122.4, 122.4, 122.3, 122.2, 122.0, 121.6, 35.8, 29.7, 27.7.

HRMS (+p ESI): calc. mass for $C_{152}H_{89}O_{16}N_4{}^{79}Br_{24}{}^{81}Br_8{}^{103}Rh_2$ [M + H]$^+$ - 4972.8083; obs. mass for $C_{152}H_{89}O_{16}N_4{}^{79}Br_{24}{}^{81}Br_8{}^{103}Rh_2$ [M + H]$^+$ - 4972.8246 [instrument does not have the resolution to completely resolve all of the Br isotopes, especially with all the bromines involved].

$[\alpha]^{20}_D$: +146.1 (c 0.260 g/100 mL, EtOAc).

Melting Point (°C): >210 °C.

4. Initial Achiral Catalyst Screen

Warning: This project involves the use of diazo compounds. Diazo compounds are known to have thermal stability issues and are explosive hazards; work with diazo compounds should be performed in a well-ventilated hood, require the use of PPE, and careful handling of the reagents.[1]

Optimization

I

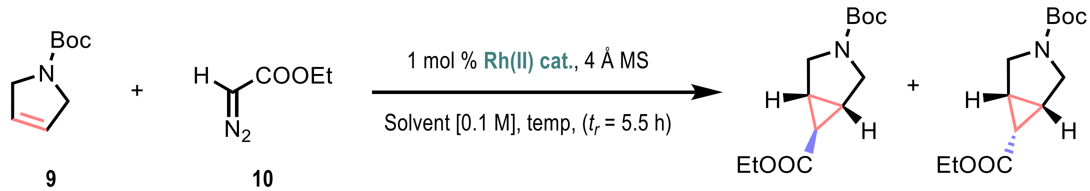

9 2 equiv | **10** 1 equiv, 3 h addition of diazo | *exo*-**1** | *endo*-**1**

Entry	Rh(II) cat.	Solvent	Temp (°C)	% yield (**1**)[c]	d.r. (*exo*:*endo*)[b]
1[a]	$Rh_2(OAc)_4$	CH_2Cl_2	25	52	66:34
2	$Rh_2(OAc)_4$	CH_2Cl_2	25	72	60:40
4	$Rh_2(OAc)_4$	DMC	25	63	60:40
8	$Rh_2(OPiv)_4$	DMC	25	79	52:48
9	$Rh_2(TPA)_4$	DMC	25	76	51:49
10	$Rh_2(Oct)_4$	DMC	25	75	57:43
11	$Rh_2(esp)_2$	DMC	25	75	44:66

II

Monodentate Achiral Rh(II) Tetracarboxylate Catalysts

Bridged (Bidentate) Achiral Rh(II) Tetracarboxylate Catalysts

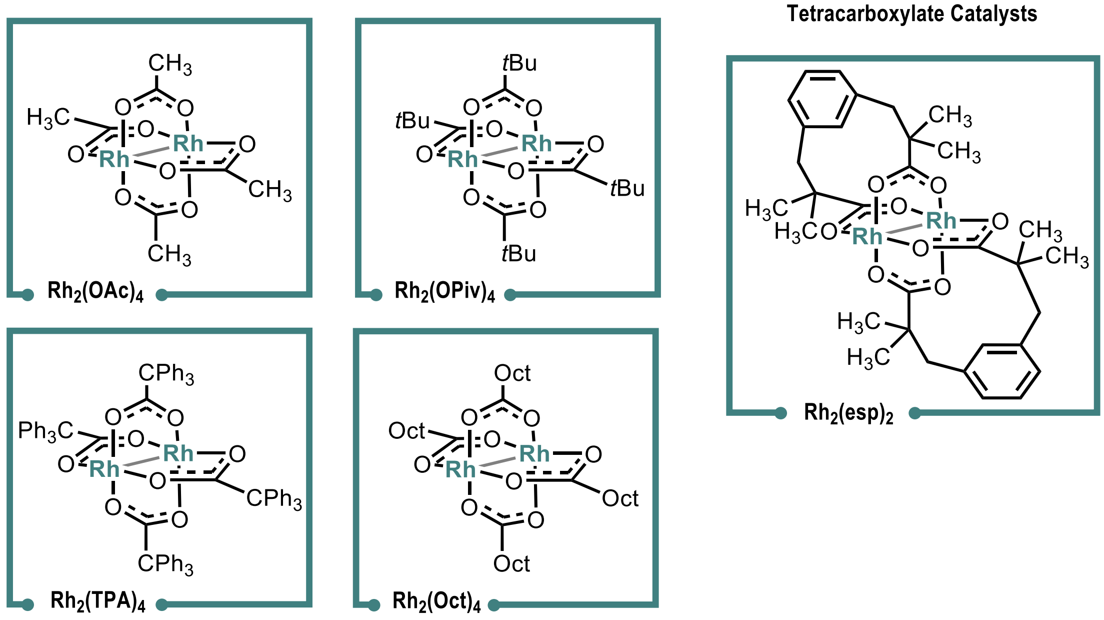

Table S1. Initial catalyst screen at 0.500 mmol scale; **General Procedure C, strategy i** was used to perform the initial catalyst screens. I) Dimethyl carbonate is DMC. [a]The equiv is inverted; equiv of diazo is 2 equiv and alkene trap is 1 equiv. [b]d.r. was calculated from ^1H NMR by integrating broad multiplet at 2.10 – 2.02 ppm (*exo* ethyl ester 2H) & multiplet 1.89 – 1.82 ppm (*endo* ethyl ester 2H). [c]^1H NMR yield was calculated by quantitative ^1H NMR analysis with 1,3,5-trimethoxybenzene as an internal standard by

integrating 6.01 ppm (s, aromatic 3H) of 1,3,5-trimethoxybenzene and at 3.38 – 3.32 (m, 2H) of *exo*/*endo*-**1**. II) Achiral dirhodium(II) tetracarboxylate catalysts used in screen.

5. Chiral Catalyst Screen for *Endo*-Selectivity

Warning: This project involves the use of diazo compounds. Diazo compounds are known to have thermal stability issues and are explosive hazards; work with diazo compounds should be performed in a well-ventilated hood, require the use of PPE, and careful handling of the reagents.[1]

Optimization

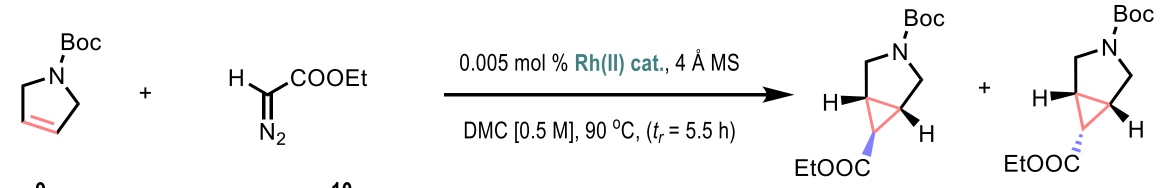

9
2 equiv

10
1 equiv
3 h addition of EDA

exo-**1** *endo*-**1**

Entry	Rh(II) cat.	% yield (**1**)[a]	d.r. (exo:endo)[b]	TON[c]
1	$Rh_2(S\text{-DOSP})_4$	8	45:55	1,600
2[d]	$Rh_2(S\text{-TPPTTL})_4$	59	24:76	11,800
3	$Rh_2(S\text{-PTAD})_4$	17	40:60	3,400
4	$Rh_2(S\text{-TCPTAD})_4$	32	32:68	6,400
5	$Rh_2(S\text{-}di\text{-(3,5-diBr)TPPTTL})_4$	26	20:80	5,200
6	$Rh_2[S\text{-}tetra\text{-(3,5-di-Br)TPPTTL}]_4$	70	17:83	14,000
7	$Rh_2(S\text{-}di\text{-(4-Br)TPPTTL})_4$	30	23:77	6,000
8	$Rh_2(S\text{-}tetra\text{-(4-Br)TPPTTL})_4$	23	21:79	4,600
9	$Rh_2(S\text{-}p\text{-Br-TPCP})_4$	17	37:63	3,400
10	$Rh_2(S\text{-}p\text{-Ph-TPCP})_4$	12	26:74	2,400
11	$Rh_2(S\text{-NTTL})_4$	53	27:73	10,600
Gram-Scale Synthesis				
12[e]	$Rh_2[S\text{-}tetra\text{-(3,5-di-Br)TPPTTL}]_4$	83[g]	17:83	16,600
13[e,f]	$Rh_2[S\text{-}tetra\text{-(3,5-di-Br)TPPTTL}]_4$	60[g]	19:81	12,000

Table S2. Chiral dirhodium(II) tetracarboxylate catalyst screen for *endo*-selectivity at 0.500 mmol scale; **General Procedure C, strategy i** was used to perform the catalyst screens. [a]^{1}H NMR yield was calculated by quantitative ^{1}H NMR analysis with 1,3,5-trimethoxybenzene as an internal standard by integrating 6.01 ppm (s, aromatic 3H) of 1,3,5-trimethoxybenzene and at 3.38 – 3.32 (m, 2H) of *exo/endo*-**1**. [b]d.r. was calculated from ^{1}H NMR by integrating broad multiplet at 2.10 – 2.02 ppm (*exo* ethyl ester 2H) & multiplet 1.89 – 1.82 ppm (*endo* ethyl ester 2H). [c]TON is calculated by [(1 equiv of limiting reagent / x equiv of catalyst)] x [(% yield / 100)]. [d]When $Rh_2(R\text{-TPPTTL})_4$ was used, the same selectivity was observed (d.r. 23:77). [e]10 mmol reaction was performed. [f]15.7% EDA in toluene was used instead of 74% EDA in CH_2Cl_2. [g]Isolated yield.

6. *Exo*/*Endo* Base Isomerization Optimization Study

General Procedure A: Base Isomerization of Mixture of *Exo*/*Endo* Esters

To a flame-dried 2 mL vial charged with a stir-bar was added the 3-(*tert*-butyl) 6-ethyl 3-azabicyclo[3.1.0]hexane-3,6-dicarboxylate (*exo*/*endo*-**1**) (128 mg, 1 equiv, 0.500 mmol). The vessel was sealed and backfilled with nitrogen three times before equipping a nitrogen balloon to the septum. To the reaction vessel was added the solvent (if the desired base is not a commercial stock solution) and the desired base (3 equiv, 1.5 mmol; or 10 equiv, 5 mmol). The reaction mixture was allowed to stir at the desired temperature (°C) for the desired time (min). Afterwards, the reaction mixture was diluted with saturated bicarbonate solution, extracted with diethyl ether three times, the organic layer dried with $MgSO_4$, filtered, and the filtrate concentrated *in vacuo* at 45 °C. The dried material was then subjected to ^{1}H NMR analysis for d.r. determination of *exo*/*endo* esters.

Optimization

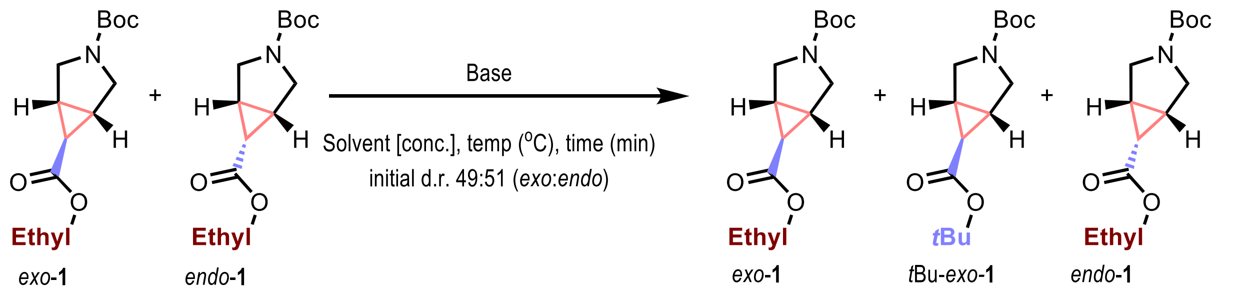

Entry	Base (equiv)	Solvent	Temp (°C)	Ethyl:*t*Bu Ester Ratio[a]	Time (min)	Combined Yield (%)[b]	d.r. (*exo*:*endo*)[c]
1	NaO*t*Bu (3)	THF [2 M]	25	59:41	10	96	>30:1
2	NaO*t*Bu (10)	THF [2 M]	25	34:66	30	64	>30:1
3	NaO*t*Bu (3)	THF [2 M]	-78	100:0	30	97	49:51
4	NaO*t*Bu (3)	DMC [0.5 M]	25	98:2	30	86	50:50
5	KO*t*Bu (3)	THF [1 M]	25	59:41	30	92	>30:1
6[e]	KO*t*Bu (3)	THF [2 M]	25	32:68	10	20	7:93
7[e]	KO*t*Bu (10)	THF [2 M]	25	-	10	0	-
8[e]	KO*t*Bu (3)	THF [2 M]	-78	83:17	30	92	50:50
9[e]	KO*t*Bu (3)	DMC [0.5 M]	25	100:0	30	96	50:50
10	NaOEt (3)	21% in EtOH	25	-	30	74	33:67
11	DBU (3)	ACN [0.5 M]	90	-	1,080	quant	48:52
12	DBU (3)	THF [0.5 M]	25	-	1,080	quant	46:54
13	DBU (3)	THF [0.5 M]	60	-	1,080	quant	48:52
14	TBD (3)	ACN [0.5 M]	90	-	1,080	47	65:35
15	BTMG (3)	DMC [0.1 M]	25	-	30	*n.d.*	44:56[d]
16	BTMG (3)	DMC [0.1 M]	70	-	30	*n.d.*	45:55[d]

Table S3. *Exo*/*Endo* Isomerization Optimizations. Reactions are ran at 0.500 mmol. [a]Ratio between ethyl vs *tert*-butyl ester was calculated from ^{1}H NMR by integrating broad multiplet 2.10 – 2.02 ppm (*exo* ethyl ester 2H) and broad multiplet 2.02 – 1.94 ppm (*exo tert*-butyl ester 2H), respectively. [b]Combined yield consist of both ethyl and *tert*-butyl ester products (if *tert*-butoxide base is used). [c]d.r. was calculated from ^{1}H NMR by integrating broad multiplet at 2.10 – 2.02 ppm (*exo* ethyl ester 2H) & multiplet 1.89 – 1.82 ppm (*endo* ethyl ester 2H). [d]Initial d.r. is 46:54 (*exo*:*endo*). [e]Solid KO*t*Bu that had been chronically exposed to moisture was used and dissolved in its respective solvent.

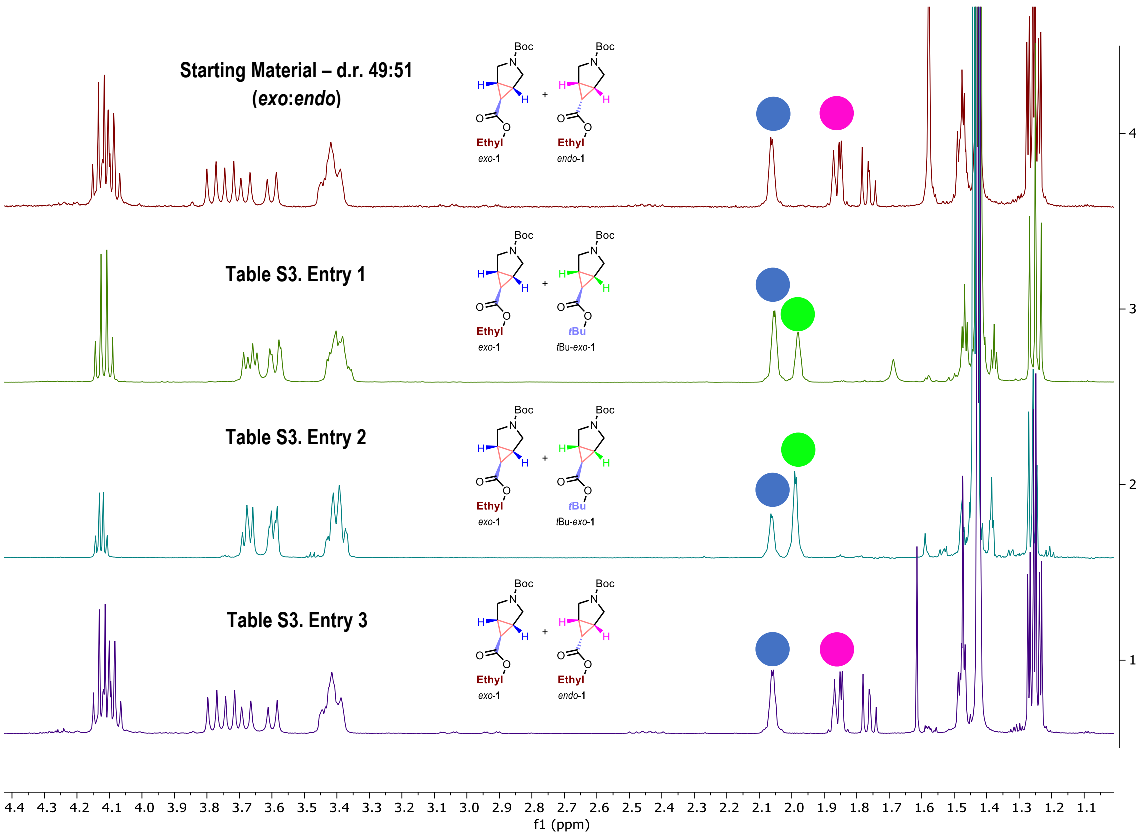

Figure S3. Representative ^{1}H NMR spectra of base isomerization from Table S3, entry 1- 3.

7. Selective Hydrolysis Optimization

General Procedure B: Optimization of Selective Hydrolysis of *Exo*/*Exo* Ester

To a 4 mL vial charged with a stir-bar was added 3-(*tert*-butyl) 6-ethyl 3-azabicyclo[3.1.0]hexane-3,6-dicarboxylate (*exo*/*endo*-**1**) (128 mg, 1 equiv, 0.500 mmol), the desired alcohol (500 µL, 1 molar, 1 equiv, 0.500 mmol), and 1 M sodium hydroxide in deionized water (20.0 mg, 500 µL, 1 molar, 1 equiv, 500 µmol). The reaction was stirred at room temperature for the desired time (min). After the elapsed time, the reaction mixture was extracted with hexanes three times, the organic layer dried with $MgSO_4$, filtered, and the filtrate concentrated *in vacuo* at 45 ^{o}C. The dried material was then subjected to ^{1}H NMR for d.r. determination of *exo*/*endo* esters.

Optimization

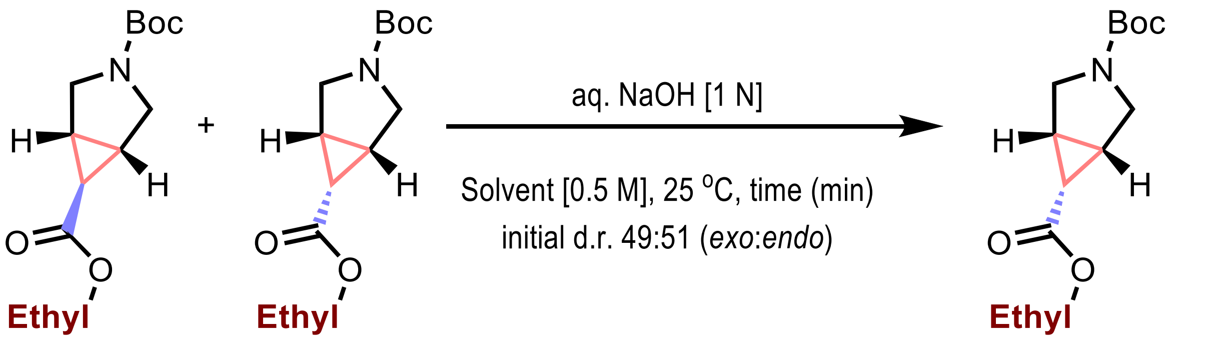

exo-**1** *endo*-**1** *endo*-**1**

Entry	Solvent	Time (min)	Yield % (Recovered %[a])	d.r. (*exo*:*endo*)[b]
1	THF	120	71	36:64
2	THF	1440	12 (23)	<1:30
3	MeOH	120	25[c] (47)	<1:30
4	MeOH	1440	7[d] (14)	<1:30
5	EtOH	10	52	1:3
6	EtOH	30	39	1:16
7	EtOH	60	35 (68)	<1:30
8[e]	EtOH	120	37 (72)	<1:30

Table S4. Selective hydrolysis of a mixture of 49:51 *exo*/*endo* ethyl esters. [a]Recovered % yield was calculated by ([yield % / 51 *endo*] x [$\frac{product\ endo\ d.r.}{sum(product\ exo/endo\ d.r.)}$]) x 100%. [b]d.r. was calculated from ^{1}H NMR by integrating broad multiplet at 2.10 – 2.02 ppm (*exo* ester 2H) and multiplet at 1.89 – 1.82 ppm (*endo* ester 2H). [c]A 1.5:1 mixture of *endo* methyl ester vs. *endo* ethyl ester was observed by integrating singlet 1.43 ppm (*endo* methyl ester's *t*Bu 9H) and singlet 1.42 (*endo* ethyl ester's *t*Bu 9H) in ^{1}H NMR. [d]A 5.5:1 mixture of *endo* methyl ester vs. *endo* ethyl ester was observed by integrating singlet 1.43 ppm (*endo* methyl ester's *t*Bu 9H) and singlet 1.42 (*endo* ethyl ester's *t*Bu 9H) in ^{1}H NMR. [e]A 3:1 mixture of *exo*- and *endo*-**11** was observed by integrating broad multiplet at 2.17 – 2.10 ppm (*exo* acid 2H) and multiplet at 2.01 – 1.97 ppm (*endo* acid 2H) from ^{1}H NMR after acidifying the aqueous solution (pH ~1) and extracting with ethyl acetate.

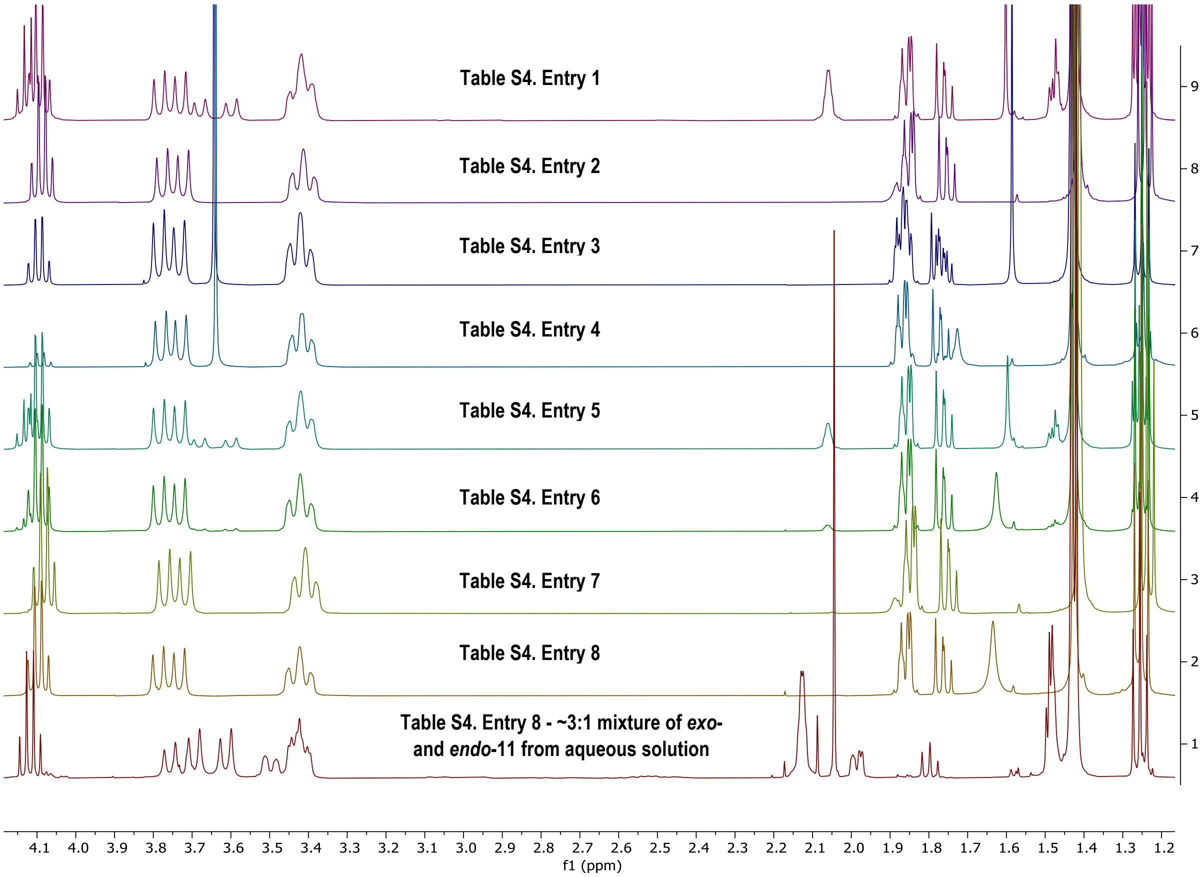

Figure S4. Representative ^{1}H NMR spectra of selective hydrolysis from Table S4, entry 1- 8.

Optimization

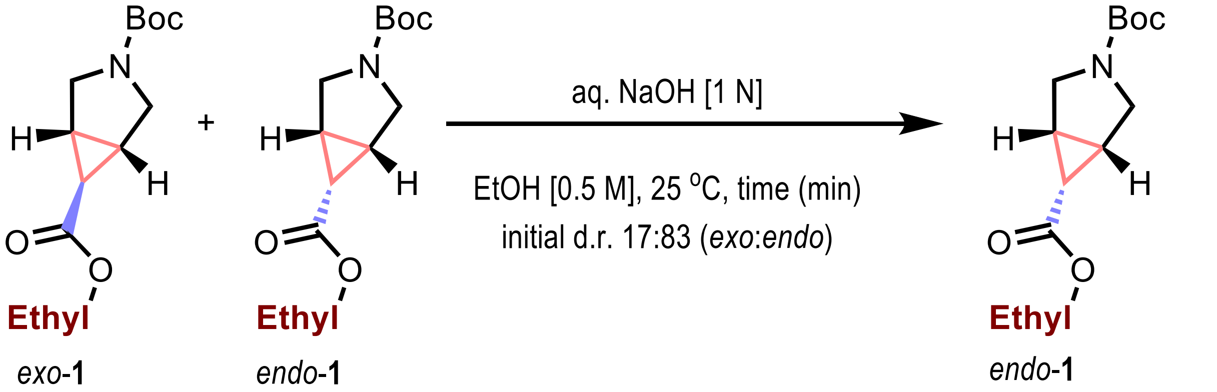

Entry	Time (min)	Yield % (Recovered %[a])	d.r. (exo:endo)[b]
1	10	66 (76)	1:20
2	30	58 (69)	<1:30
3	60	49 (59)	<1:30
4	120	42 (51)	<1:30

Table S5. Selective hydrolysis of a mixture of 17:83 *exo*/*endo* ethyl esters. [a]Recovered % yield was calculated by ([yield % / 83 *endo*] x [$\frac{product\ endo\ d.r.}{sum(product\ exo{:}endo\ \ d.r.)}$]) x 100%. [b]d.r. was calculated from ^{1}H NMR by integrating broad multiplet at 2.10 – 2.02 ppm (*exo* ester 2H) and multiplet 1.89 – 1.82 ppm (*endo* ester 2H).

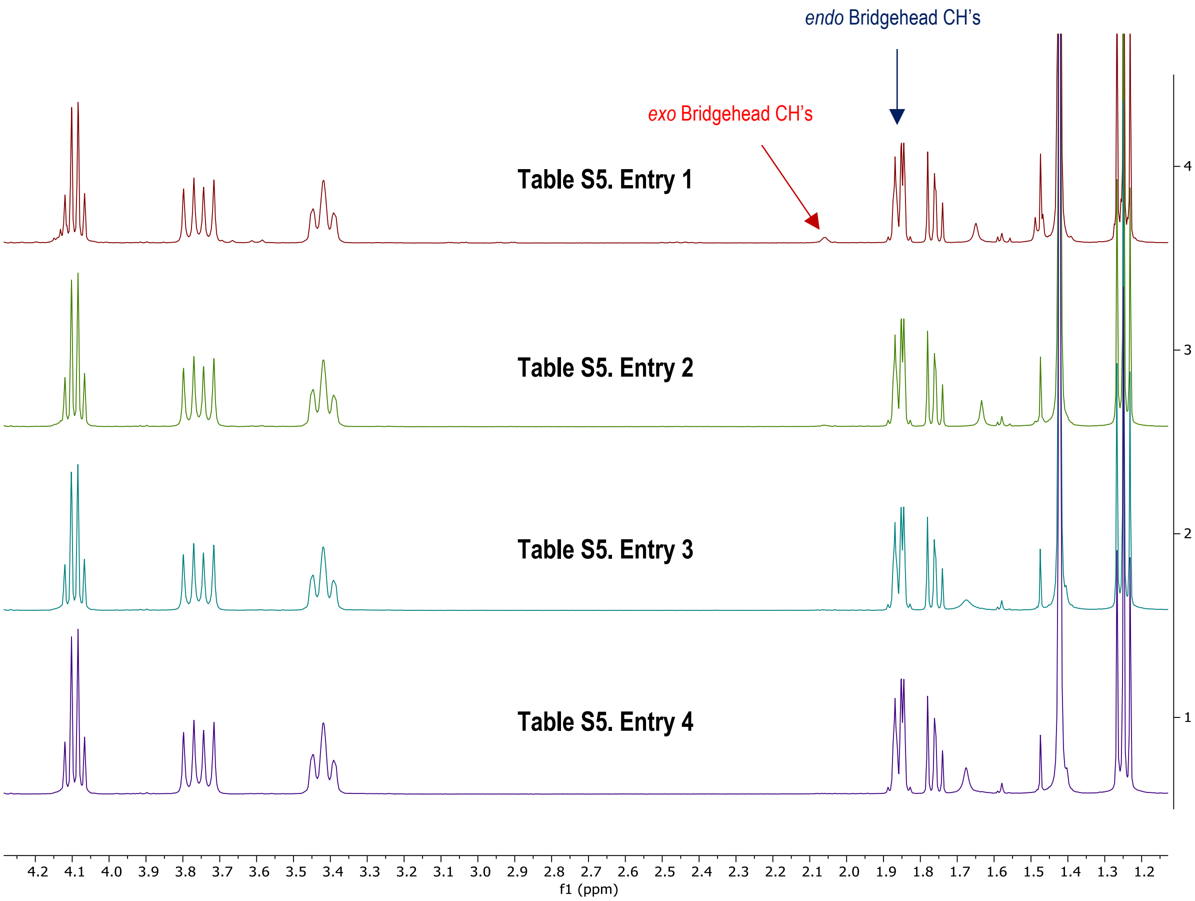

Figure S5. Representative ^{1}H NMR spectra of selective hydrolysis from Table S5, entry 1- 4.

8. High TON, Low Catalyst Loading and Telescoped Procedures

Warning: This project involves the use of diazo compounds. Diazo compounds are known to have thermal stability issues and are explosive hazards; work with diazo compounds should be performed in a well-ventilated hood, require the use of PPE, and careful handling of the reagents.[1]

General Procedure C: General High TON, Low Catalyst Loading Rh(II) [2+1] Cycloaddition Procedure

Sample calculations for making catalyst stock solution:

Stock solution of $Rh_2(esp)_2$ in CH_2Cl_2 – $\frac{0.0190\ mg\ of\ catalyst}{50\ \mu L\ of\ CH2Cl2} = \frac{3.46\ mg\ of\ catalyst\ weighed}{x\ \mu L\ of\ CH2Cl2}$;

x = 9,105 µL of CH_2Cl_2 is used to dissolve 3.46 mg of $Rh_2(esp)_2$ to create a 0.00050 M stock solution

To a flame-dried vessel charged with a stir-bar and activated 4 Å MS (1g of activated 4 Å MS/ 1 mmol of diazo) was added the alkene trap (2 equiv) to the vessel. The vessel was backfilled with nitrogen three times and then capped with a nitrogen balloon. The desired solvent was added to the reaction vessel. Subsequently, a stock solution of dirhodium(II) tetracarboxylate catalyst (0.00050 molar, .00005 equiv) in CH_2Cl_2 was added to the vessel. The reaction vessel containing trap and catalyst was stirred at 300 rpm at the desired temperature for at least 10 minutes. After the elapsed time, to the reaction solution was added ethyl 2-diazoacetate (74% Wt, 1 equiv) in CH_2Cl_2 to the reaction vessel via a dual-syringe pump over 3 h. The needle was submerged into the reaction solution. A **strategy (i, ii, iii, iv)** was then selected below:

i. **<u>qNMR method:</u>** After complete addition and elapsed time of 3 h, the resulting solution was reacted for an additional 2.5 h and then subsequently, cooled to room temperature, filtered over a plug of celite, and the filtrate concentrated *in vacuo* over 50 ^oC in a tared vial. The crude mass recovery was recorded. Subsequently, a known amount of 1,3,5-trimethoxybenzene was added to the crude material serving as an internal standard. The mixture was dissolved in $CDCl_3$ and a ^1H NMR was performed on the material to determine the ^1H NMR yield via qNMR; the relaxation times were generally conducted at 2 - 5 s. Integrations at 6.01 ppm (s, aromatic 3H) of 1,3,5-trimethoxybenzene and at 3.38 – 3.32 (m, 2H) of 3-(*tert*-butyl) 6-ethyl (1R,5S)-3-azabicyclo[3.1.0]hexane-3,6-dicarboxylate (*exo*/*endo*-**1**) were measured.

<u>Sample calculations for qNMR analysis:</u>

$$P_{sample} = (I_{Analyte} / I_{CRM}) \times (N_{CRM} / N_{Analyte}) \times (M_{Analyte} / M_{CRM}) \times (m_{CRM} / m_{Sample}) \times (P_{CRM})$$

P_{sample} = (4.22 / 1.00) x (3H / 2H) x (255.31 g/mol / 168.07 g/mol) x (10.2 mg of trimethoxybenzene / 198.7 mg of crude) x (99% purity of trimethoxybenzene) = **48.8% purity** for 3-(*tert*-butyl) 6-ethyl (1R,5S)-3-azabicyclo[3.1.0]hexane-3,6-dicarboxylate (*exo*/*endo*-**1**)

qNMR yield = [(198.7 mg of crude) x (48.8% purity for 3-(*tert*-butyl) 6-ethyl 3-azabicyclo[3.1.0]hexane-3,6-dicarboxylate / 100%)] x (1 / 128 mg theoretical mass) x (100%) = **75.8% qNMR yield** of 3-(*tert*-butyl) 6-ethyl (1R,5S)-3-azabicyclo[3.1.0]hexane-3,6-dicarboxylate (*exo*/*endo*-**1**)

Refer to Sigma-Aldrich "Quantitative NMR Technical Details and *Trace***CERT** Certified Reference Materials" manual for more detailed information. https://www.sigmaaldrich.com/deepweb/assets/sigmaaldrich/marketing/global/documents/101/854/qnmr-brochure-rjo.pdf

ii. **<u>Rh(II) [2+1] Followed By Kugelrohr Distillation:</u>** After complete addition and elapsed time of 3 h, the resulting solution was reacted for an additional 2.5 h and then subsequently, cooled to room temperature, filtered over a plug of celite, and the filtrate concentrated *in vacuo* over 50 ^oC. The straw-colored crude material was subjected to a Kugelrohr distillation. The initial distillation occurred at 110 ^oC at 5 to 6 $x10^{-1}$ torr for at least 1 h, which the trap distilled over and removed from the collecting bulbs. Then the distillation resumed and was heated up to 170 ^oC at 5 - 6 x 10^{-1} torr until all product distilled into the collecting bulbs. The distillate was collected to afford clean 3-(*tert*-butyl) 6-ethyl 3-azabicyclo[3.1.0]hexane-3,6-dicarboxylate (*exo*/*endo*-**1**) as a straw-colored oil.

iii. **<u>Telescoped Reaction to Afford *Exo*-Acids:</u>** After complete addition and elapsed time of 3 h, the resulting solution was reacted for an additional 2.5 h and then subsequently, cooled to room temperature, filtered over a plug of celite, and the filtrate concentrated *in vacuo* over 50 °C in a round-bottom flask. The crude material was then subjected to the isomerization conditions.

To a round-bottom flask charged with a stir-bar and the crude was added 2 M sodium *tert*-butoxide in THF (2 molar, 3 equiv). The reaction mixture was allowed to stir for at the desired amount of time (min). To the reaction mixture was then added ethanol (85.5 equiv) and then 2 M sodium hydroxide in deionized water (2 molar, 10 equiv). The reaction mixture was allowed to stir at 25 °C for at least 24 h. After the elapsed time, the mixture was concentrated *in vacuo* at 55 °C to get rid of any organic volatiles. To the concentrated aqueous solution was washed with diethyl ether five times. Afterwards, the aqueous solution was placed in a saltwater ice bath, cooled, and was slowly acidified with 1 M HCl to a pH of 1 - 2. Subsequently, the aqueous solution was diluted with ethyl acetate, extracted with ethyl acetate three times, the organic layer dried with $MgSO_4$, filtered, and the filtrate concentrated *in vacuo* at 55 °C to afford the desired *exo*-acid.

iv. **<u>Telescoped Reaction to Afford *Endo*-Acids:</u>** After complete addition and elapsed time of 3 h, the resulting solution was reacted for an additional 2.5 h and then subsequently, cooled to room temperature, filtered over a plug of celite, and the filtrate concentrated *in vacuo* over 50 °C. The crude straw-colored material was then subjected to the selective hydrolysis conditions.

To a round-bottom flask charged with a stir-bar and the crude straw-colored material was added ethanol (1 molar), and 1 M sodium hydroxide in deionized water (1 molar, 1 equiv). The reaction mixture was stirred at 25 °C for a certain amount of time (min). The reaction mixture was then extracted with hexanes five times, the organic layer dried with $MgSO_4$, filtered, and the filtrate concentrated *in vacuo* at 50 °C. To the crude in a round-bottom flask was charged with a stir-bar and was then added ethanol (85.5 equiv) and then 2 M sodium hydroxide in deionized water (2 molar, 10 equiv). The reaction mixture was allowed to stir at 25 °C for at least 24 h. After the elapsed time, the mixture was concentrated *in vacuo* at 55 °C to get rid of any organic volatiles. To the concentrated aqueous solution was washed with diethyl ether five times. Afterwards, the aqueous solution was placed in a saltwater ice bath, cooled, and was slowly acidified with 1 M HCl to a pH of 1 - 2. Subsequently, the aqueous solution was diluted with ethyl acetate, extracted with ethyl acetate three times, the organic layer dried with $MgSO_4$, filtered, and the filtrate concentrated *in vacuo* at 55 °C to afford the desired *endo*-acid.

General Procedure D: One-Pot Tandem Isomerization Followed by *Exo*-Hydrolysis to Afford *Exo*-Acid

Starting with clean *exo/endo* ester (1 equiv): a round-bottom flask charged with a stir-bar and the clean *exo/endo* ester (1 equiv) was added 2 M sodium *tert*-butoxide in THF (2 molar, 3 equiv). The reaction mixture was allowed to stir at room temperature for a certain amount of time (min). To the reaction mixture was then added ethanol (85.5 equiv) and then 2 M sodium hydroxide in deionized water (2 molar, 10 equiv). The reaction mixture was allowed to stir at 25 °C for 24 h. After the elapsed time, the mixture was concentrated *in vacuo* at 45 °C to get rid of any organic volatiles. Afterwards, the aqueous solution was placed in a saltwater ice bath, cooled, and was slowly acidified with 1 M HCl to a pH of 1 - 2. Subsequently, the aqueous solution was diluted with ethyl acetate, extracted with ethyl acetate three times, the organic

layer dried with $MgSO_4$, filtered, and the filtrate concentrated *in vacuo* at 50 °C to afford the desired *exo*-acid.

General Procedure E: Selective Hydrolysis Followed by *Endo*-Hydrolysis to Afford *Endo*-Acid

Part I: To a vial charged with a stir-bar was added the *exo/endo*-ester (1 equiv), ethanol (1 molar), and 1 M sodium hydroxide in deionized water (1 molar, 1 equiv). The reaction was capped and stirred at 25 °C for 30 min. After the elapsed time, the reaction mixture was extracted with hexanes three times, the organic layer dried with $MgSO_4$, filtered, and the filtrate concentrated *in vacuo* at 45 °C to afford the enriched *endo*-ester.

Part II: To a vial charged with a stir-bar and the enriched *endo*-ester (1 equiv) was added ethanol (85.5 equiv) and 2 M sodium hydroxide in deionized water (2 molar, 10 equiv) and was capped. The reaction solution was stirred vigorously at room temperature for at least 24 h. After the elapsed time, the mixture was washed with hexanes three times and then the aqueous solution was concentrated *in vacuo* at 45 °C to get rid of any organic volatiles. The concentrated aqueous solution was placed in a saltwater ice bath, cooled, and was slowly acidified with 1 M HCl to a pH of 1 - 2. Subsequently, the aqueous solution was diluted with ethyl acetate, extracted with ethyl acetate three times, the organic layer dried with $MgSO_4$, filtered, and filtrate concentrated *in vacuo* at 50 °C to afford the desired *endo*-acid.

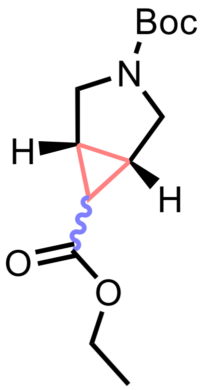

3-(*tert*-butyl) 6-ethyl (1R,5S)-3-azabicyclo[3.1.0]hexane-3,6-dicarboxylate (*Exo/endo*-1)

From **General Procedure C**, **strategy ii** with **Rh(II)$_2$(esp)$_2$**. To a flame-dried 50 mL round-bottom flask charged with an octagonal stir-bar (1 in x 5/16 in) stir-bar and 10 g of activated 4 Å MS was added *tert*-butyl 2,5-dihydro-1H-pyrrole-1-carboxylate (**9**) (3.384 g, 2 equiv, 20.00 mmol) to the vessel. The vessel was backfilled with nitrogen three times and then capped with a nitrogen balloon. The DMC (17.57 mL) was added to the reaction vessel. Subsequently, a stock solution of Rh(II)$_2$(esp)$_2$ in CH_2Cl_2 (379.2 µg, 1.000 mL, 0.00050 molar, .00005 equiv, 0.5000 µmol) was added to the vessel. The reaction vessel containing trap and catalyst was stirred at 300 rpm at 90 °C for at least 10 minutes. After the elapsed time, to the reaction solution was added ethyl 2-diazoacetate (**10**) in CH_2Cl_2 (1.542 g, 1.421 mL, 74% Wt, 1 equiv, 10.00 mmol) to the reaction vessel via a dual-syringe pump [dual-syringe pump settings -- 3 mL syringe, 0.475 mL/hr, diameter 9.83 mm, vol 1.425 mL; Air-Tite/SilverPoint 22Gx4" long hypodermic needle was used] over 3 h. The needle was submerged into the reaction solution.

After complete addition and elapsed time of 3 h, the resulting solution was reacted for an additional 2.5 h and then subsequently, cooled to room temperature, filtered over a plug of celite, and the filtrate concentrated *in vacuo* over 50 °C. The straw-colored crude material was subjected to a Kugelrohr

distillation. The initial distillation occurred at 110 °C at 5 to 6 x10^{-1} torr for at least 1 h, which *tert*-butyl 2,5-dihydro-1H-pyrrole-1-carboxylate (**9**) distilled over and removed from the collecting bulbs. Then the distillation resumed and was heated up to 170 °C at 5 - 6 x 10^{-1} torr until all product distilled into the collecting bulbs. The distillate was collected to afford clean 3-(*tert*-butyl) 6-ethyl (1R, 5S)-3-azabicyclo[3.1.0]hexane-3,6-dicarboxylate (*exo*/*endo*-**1**) (2.30 g, 9.01 mmol, 90.1% yield, *exo*:*endo* d.r. 49:51) as a straw-colored oil. NMR data is in good agreement with literature precedent.[11]

From **General Procedure C**, **strategy ii** with **Rh(II)$_2$[*S*-*tetra*-(3,5-di-Br)TPPTTL]$_4$**. To a flame-dried 50 mL round-bottom flask charged with an octagonal stir-bar (1 in x 5/16 in) stir-bar and 10 g of activated 4 Å MS was added *tert*-butyl 2,5-dihydro-1H-pyrrole-1-carboxylate (**9**) (3.384 g, 2 equiv, 20.00 mmol) to the vessel. The vessel was backfilled with nitrogen three times and then capped with a nitrogen balloon. The DMC (17.57 mL) was added to the reaction vessel. Subsequently, a stock solution of Rh(II)$_2$[*S*-*tetra*-(3,5-di-Br)TPPTTL]$_4$ in CH_2Cl_2 (2.495 mg, 1.000 mL, 0.00050 molar, .00005 equiv, 0.5000 µmol) was added to the vessel. The reaction vessel containing trap and catalyst was stirred at 300 rpm at 90 °C for at least 10 minutes. After the elapsed time, to the reaction solution was added ethyl 2-diazoacetate (**10**) in CH_2Cl_2 (1.542 g, 1.421 mL, 74% Wt, 1 equiv, 10.00 mmol) to the reaction vessel via a dual-syringe pump [dual-syringe pump settings -- 3 mL syringe, 0.475 mL/hr, diameter 9.83 mm, vol 1.425 mL; Air-Tite/SilverPoint 22Gx4" long hypodermic needle was used] over 3 h. The needle was submerged into the reaction solution.

After complete addition and elapsed time of 3 h, the resulting solution was reacted for an additional 2.5 h and then subsequently, cooled to room temperature, filtered over a plug of celite, and the filtrate concentrated *in vacuo* over 50 °C. The straw-colored crude material was subjected to a Kugelrohr distillation. The initial distillation occurred at 110 °C at 5 to 6 x10^{-1} torr for at least 1 h, which *tert*-butyl 2,5-dihydro-1H-pyrrole-1-carboxylate (**9**) distilled over and removed from the collecting bulbs. Then the distillation resumed and was heated up to 170 °C at 5 - 6 x 10^{-1} torr until all product distilled into the collecting bulbs. The distillate was collected to afford clean 3-(*tert*-butyl) 6-ethyl (1R, 5S)-3-azabicyclo[3.1.0]hexane-3,6-dicarboxylate (*exo*/*endo*-**1**) (2.12 g, 8.30 mmol, 83.0% yield, *exo*:*endo* d.r. 17:83) as a straw-colored oil. NMR data is in good agreement with literature precedent.[11]

^{1}H NMR (400 MHz, $CDCl_3$) δ [49:51 mixture of *exo*:*endo*] 4.15 – 4.07 (*exo*/*endo* m, 4.7H), 3.80 – 3.72 (*endo* dd, J = 21.9, 11.1 Hz, 2.1H), 3.69 – 3.58 (*exo* dd, J = 32.4, 11.2 Hz, 2H), 3.45 – 3.38 (*exo*/*endo* dtd, J = 11.1, 7.9, 7.5, 4.1 Hz, 4.1H), 2.07 – 2.05 (*exo* m, 2H), 1.89 – 1.83 (*endo* ddd, J = 7.7, 3.3, 1.4 Hz, 2.1H), 1.78 – 1.74 (*endo* dd, J = 8.8, 7.3 Hz, 1.0H), 1.49 (*exo* d, J = 2.7 Hz, 0.9H), 1.43 (*exo* s, 8.7H), 1.42 (*endo* s, 9.3H), 1.25 (*exo*/*endo* td, J = 7.2, 2.9 Hz, 7.2H).

HRMS (+p ESI): calc. mass for $C_{13}H_{22}O_4N$ $[M + H]^+$ - 256.1543; obs. mass for $C_{13}H_{22}O_4N$ $[M + H]^+$ - 256.1541.

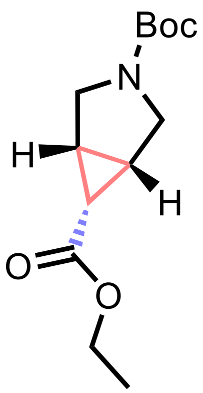

3-(*tert*-butyl) 6-ethyl (1R,5S,6s)-3-azabicyclo[3.1.0]hexane-3,6-dicarboxylate (*endo*-1)

From **General Procedure E, Part I** with **1:1 mixture** of *exo*/*endo*-ester. To a 4 mL vial charged with a stir-bar was added 3-(*tert*-butyl) 6-ethyl (1R,5S)-3-azabicyclo[3.1.0]hexane-3,6-dicarboxylate (*exo*/*endo*-**1**) (128 mg, 1 equiv, 0.500 mmol, *exo*:*endo* d.r. 49:51), ethanol (23.0 mg, 500 µL, 1 molar, 1 equiv, 500 µmol), and 1 M sodium hydroxide in deionized water (20.0 mg, 500 µL, 1 molar, 1 equiv, 500 µmol). The reaction was capped and stirred at 25 °C for 120 min. After the elapsed time, the reaction mixture was extracted with hexanes three times, the organic layer dried with $MgSO_4$, filtered, and the filtrate concentrated *in vacuo* at 45 °C to afford 3-(*tert*-butyl) 6-ethyl (1R,5S,6s)-3-azabicyclo[3.1.0]hexane-3,6-dicarboxylate (*endo*-**1**) (47.4 mg, 186 µmol, 37.1% yield, 72% recovered yield, *exo*:*endo* d.r. <1:30) as a clear non-colored oil.

From **General Procedure E, Part I** with **enriched *endo* ester** (*exo*:*endo* d.r. 17:83). To a 4 mL vial charged with a stir-bar was added 3-(*tert*-butyl) 6-ethyl (1R,5S)-3-azabicyclo[3.1.0]hexane-3,6-dicarboxylate (*exo*/*endo*-**1**) (128 mg, 1 equiv, 0.500 mmol, *exo*:*endo* d.r. 17:83), ethanol (23.0 mg, 500 µL, 1 molar, 1 equiv, 500 µmol), and 1 M sodium hydroxide in deionized water (20.0 mg, 500 µL, 1 molar, 1 equiv, 500 µmol). The reaction was capped and stirred at 25 °C for 30 min. After the elapsed time, the reaction mixture was extracted with hexanes three times, the organic layer dried with $MgSO_4$, filtered, and the filtrate concentrated *in vacuo* at 45 °C to afford 3-(*tert*-butyl) 6-ethyl (1R,5S,6s)-3-azabicyclo[3.1.0]hexane-3,6-dicarboxylate (*endo*-**1**) (74.3 mg, 291 µmol, 58.2% yield, 69% recovered yield, *exo*:*endo* d.r. <1:30) as a clear non-colored oil.

^{1}H NMR (400 MHz, $CDCl_3$) δ 4.11 – 4.05 (m, 2H), 3.78 – 3.70 (dd, J = 21.4, 11.1 Hz, 2H), 3.44 – 3.37 (ddd, J = 13.2, 7.8, 2.7 Hz, 2H), 1.87 – 1.81 (m, 2H), 1.78 – 1.73 (m, 1H), 1.41 (s, 9H), 1.25 – 1.22 (t, J = 7.1 Hz, 3H).

^{13}C NMR (101 MHz, $CDCl_3$) δ 168.9, 154.0, 79.3, 60.5, 45.6, 45.4, 28.4, 22.3, 21.8, 21.2, 14.2.

HRMS (+p ESI): calc. mass for $C_{13}H_{22}O_4N$ $[M + H]^+$ - 256.1543; obs. mass for $C_{13}H_{22}O_4N$ $[M + H]^+$ - 256.1538.

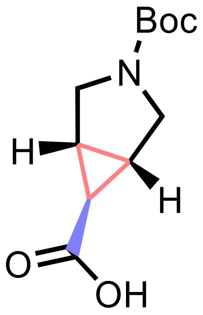

(1R,5S,6r)-3-(*tert*-butoxycarbonyl)-3-azabicyclo[3.1.0]hexane-6-carboxylic acid (*exo*-11)

From **General Procedure C**, **strategy iii**. To a flame-dried 50 mL round-bottom flask charged with an octagonal stir-bar (1 in x 5/16 in) and 10 g of activated 4 Å MS was added *tert*-butyl 2,5-dihydro-1H-pyrrole-1-carboxylate (**9**) (3.384 g, 2 equiv, 20.00 mmol) to the vessel. The vessel was backfilled with nitrogen three times and then capped with a nitrogen balloon. The DMC (17.57 mL) was added to the reaction vessel. Subsequently, a stock solution of Rh(II)$_2$(esp)$_2$ in CH_2Cl_2 (379.2 µg, 1.000 mL, 0.00050 molar, .00005 equiv, 0.5000 µmol) was added to the vessel. The reaction vessel containing trap and catalyst was stirred at 300 rpm at 90 ^oC for at least 10 minutes. After the elapsed time, to the reaction solution was ethyl 2-diazoacetate (**10**) in CH_2Cl_2 (1.542 g, 1.421 mL, 74% Wt, 1 equiv, 10.00 mmol) to the reaction vessel via a dual-syringe pump [dual-syringe pump settings -- 3 mL syringe, 0.475 mL/hr, diameter 9.8 mm, vol 1.425 mL; Air-Tite/SilverPoint 22Gx4" long hypodermic needle was used] over 3 h. The needle was submerged into the reaction solution. After complete addition and elapsed time of 3 h, the resulting solution was reacted for an additional 2.5 h and then subsequently, cooled to room temperature, filtered over a plug of celite, and the filtrate concentrated *in vacuo* over 50 ^oC in a 250 mL round-bottom flask. The crude material was then subjected to the isomerization conditions.

To a 250 mL round-bottom flask charged with a stir-bar and the crude material was added 2 M sodium *tert*-butoxide in THF (2.883 g, 15.00 mL, 2 molar, 3 equiv, 30.00 mmol). An immediate color change to brown-orange was observed with addition of base. The reaction mixture was allowed to stir for at least 10 min at room temperature to ensure complete isomerization of starting material. To the reaction mixture was then added ethanol (39.39 g, 49.9 mL, 85.5 equiv, 855.0 mmol) and then 2 M sodium hydroxide in deionized water (4.000 g, 50.00 mL, 2 molar, 10 equiv, 100.0 mmol). The reaction mixture was allowed to stir at 25 °C for 24 h. After the elapsed time, the mixture was concentrated *in vacuo* at 55 ^oC to get rid of any organic volatiles. To the concentrated aqueous solution was washed with diethyl ether five times. Afterwards, the aqueous solution was placed in a saltwater ice bath, cooled, and was slowly acidified with 1 M HCl to a pH of 1 - 2. Subsequently, the aqueous solution was diluted with ethyl acetate, extracted with ethyl acetate three times, the organic layer dried with $MgSO_4$, filtered, and the filtrate concentrated *in vacuo* at 55 ^oC to afford a dark-orange oil. The dark-orange oil was resuspended in 1:9 ether:*n*-heptane and filtered through a fine-porosity, slow flowrate filter paper under vacuum. The yellow filtrate was concentrated *in vacuo* at 45 ^oC as a light-orange oil. On the filter paper was collected a brown oily residue. The light-orange oil was dried under vacuum overnight to afford (1R,5S,6r)-3-(*tert*-butoxycarbonyl)-3-azabicyclo[3.1.0]hexane-6-carboxylic acid (*exo*-**11**) (1.72 g, 7.57 mmol, 75.7% yield *over 3 steps*, *exo*:*endo* d.r. >30:1) as a pale orange powder.

From **General Procedure D.** Starting with clean 3-(*tert*-butyl) 6-ethyl (1R,5S)-3-azabicyclo[3.1.0]hexane-3,6-dicarboxylate (*exo*/*endo*-**1**) (*exo*:*endo* d.r. ~1:1), a round-bottom flask charged with a stir-bar and the 3-(*tert*-butyl) 6-ethyl (1R,5S)-3-azabicyclo[3.1.0]hexane-3,6-dicarboxylate (*exo*/*endo*-**1**) (128 mg, 1 equiv, 0.500 mmol, *exo*:*endo* d.r. ~1:1) was added 2 M sodium *tert*-butoxide in

THF (144 mg, 750 µL, 2 molar, 3 equiv, 1.50 mmol). The reaction mixture was allowed to stir at room temperature for 10 min. An immediate color change to brown-orange was observed with addition of base. To the reaction mixture was then added ethanol (1.97 mg, 2.50 mL, 85.5 equiv, 42.8 mmol) and then 2 M sodium hydroxide in deionized water (200 mg, 2.50 mL, 2 molar, 10 equiv, 5.00 mmol). The reaction mixture was allowed to stir at 25 °C for 24 h. After the elapsed time, the mixture was concentrated *in vacuo* at 45 °C to get rid of any organic volatiles. To the concentrated aqueous solution was washed with diethyl ether five times. Afterwards, the aqueous solution was placed in a saltwater ice bath, cooled, and was slowly acidified with 1 M HCl to a pH of 1 - 2. Subsequently, the aqueous solution was diluted with ethyl acetate, extracted with ethyl acetate three times, the organic layer dried with $MgSO_4$, filtered, and the filtrate concentrated *in vacuo* at 50 °C to afford a brown oil. The brown oil was resuspended, triturated while sonicating in *n*-heptane, filtered, and the filter cake dried under vacuum to afford (1R,5S,6r)-3-(*tert*-butoxycarbonyl)-3-azabicyclo[3.1.0]hexane-6-carboxylic acid (*exo*-**11**) (98 mg, 0.43 mmol, 86% yield *over 2 steps*, *exo*:*endo* d.r. >30:1) as a pale orange powder.

^{1}H NMR (400 MHz, $CDCl_3$) δ 3.71 – 3.68 (d, J = 11.3 Hz, 1H), 3.63 – 3.60 (d, J = 11.3 Hz, 1H), 3.45 – 3.40 (td, J = 8.3, 4.2 Hz, 2H), 2.13 – 2.12 (d, J = 2.6 Hz, 2H), 1.49 – 1.48 (t, J = 3.0 Hz, 1H), 1.43 (s, 9H).

^{13}C NMR (101 MHz, $CDCl_3$) δ 178.3, 154.7, 80.0, 47.9, 47.6, 28.4, 27.3, 26.6, 24.2.

HRMS (-p APCI): calc. mass for $C_{11}H_{16}O_4N$ [M – H]$^-$ - 226.1085; obs. mass for $C_{11}H_{16}O_4N$ [M – H]$^-$ - 226.1085.

Melting Point (°C): 89 – 91 °C.

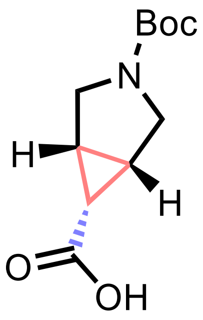

(1R,5S,6s)-3-(*tert*-butoxycarbonyl)-3-azabicyclo[3.1.0]hexane-6-carboxylic acid (*endo*-11)

From **General Procedure C**, **strategy iv**. To a flame-dried 50 mL round-bottom flask charged with an octagonal stir-bar (1 in x 5/16 in) and 10 g of activated 4 Å MS was added *tert*-butyl 2,5-dihydro-1H-pyrrole-1-carboxylate (**9**) (3.384 g, 2 equiv, 20.00 mmol) to the vessel. The vessel was backfilled with nitrogen three times and then capped with a nitrogen balloon. The DMC (17.57 mL) was added to the reaction vessel. Subsequently, a stock solution of $Rh(II)_2(esp)_2$ in CH_2Cl_2 (379.2 µg, 1.000 mL, 0.00050 molar, .00005 equiv, 0.5000 µmol) was added to the vessel. The reaction vessel containing trap and catalyst was stirred at 300 rpm at 90 °C for at least 10 minutes. After the elapsed time, to the reaction solution was ethyl 2-diazoacetate (**10**) in CH_2Cl_2 (1.542 g, 1.421 mL, 74% Wt, 1 equiv, 10.00 mmol) to the reaction vessel via a dual-syringe pump [dual-syringe pump settings -- 3 mL syringe, 0.475 mL/hr, diameter 9.8 mm, vol 1.425 mL; Air-Tite/SilverPoint 22Gx4" long hypodermic needle was used] over 3 h. The needle was submerged into the reaction solution. After complete addition and elapsed time of 3 h, the resulting solution was reacted for an additional 2.5 h and then subsequently, cooled to room temperature, filtered

over a plug of celite, and the filtrate concentrated *in vacuo* over 50 °C in a 250 mL round-bottom flask. The crude material was then subjected to the isomerization conditions.

To a 250 mL round-bottom flask charged with a stir-bar and the crude straw-colored material was added ethanol (460.7 mg, 10.00 mL, 1 molar, 1 equiv, 10.00 mmol), and 1 M sodium hydroxide in deionized water (400.0 mg, 10.00 mL, 1 molar, 1 equiv, 10.00 mmol). The reaction mixture was stirred at 25 °C for 30 min. The reaction mixture was then extracted with hexanes five times, the organic layer dried with $MgSO_4$, filtered, and the filtrate concentrated *in vacuo* at 50 °C. To the crude in a 250 mL round-bottom flask was charged with a stir-bar and was then added ethanol (39.39 g, 49.9 mL, 85.5 equiv, 855.0 mmol) and 2 M sodium hydroxide in deionized water (4.000 g, 50.00 mL, 2 molar, 10 equiv, 100.0 mmol). The reaction mixture was allowed to stir at 25 °C for 24 h. After the elapsed time, the mixture was concentrated *in vacuo* at 55 °C to get rid of any organic volatiles. To the concentrated aqueous solution was washed with hexanes five times. Afterwards, the aqueous solution was placed into a saltwater ice bath, cooled, and was slowly acidified with 1 M HCl to a pH of 1 - 2. Subsequently, the aqueous solution was diluted with ethyl acetate, extracted with ethyl acetate three times, the organic layer dried with $MgSO_4$, filtered, and the filtrate concentrated *in vacuo* at 55 °C to afford (1R,5S,6s)-3-(*tert*-butoxycarbonyl)-3-azabicyclo[3.1.0]hexane-6-carboxylic acid (*endo*-**11**) (1.22 g, 5.37 mmol, 53.7 % yield *over 3 steps*, *exo*:*endo* d.r. <1:30) as a white powder.

From **General Procedure E**, **part II**. To a 20 mL vial charged with a stir-bar and 3-(*tert*-butyl) 6-ethyl (1R,5S,6s)-3-azabicyclo[3.1.0]hexane-3,6-dicarboxylate (*endo*-**1**) (128 mg, 1 equiv, 0.500 mmol, exo:endo d.r. <1:30) was added ethanol (1.97 g, 2.50 mL, 85.5 equiv, 42.8 mmol) and 2 M sodium hydroxide in deionized water (200 mg, 2.50 mL, 2 molar, 10 equiv, 5.00 mmol) and was capped. The reaction solution was stirred vigorously at room temperature for 24 h. After the elapsed time, the mixture was washed with hexanes three times and then the aqueous solution was concentrated *in vacuo* at 45 °C to get rid of any organic volatiles. The concentrated aqueous solution was placed in a saltwater ice bath, cooled, and was slowly acidified with 1 M HCl to a pH of 1 - 2. Subsequently, the aqueous solution was diluted with ethyl acetate, extracted with ethyl acetate three times, the organic layer dried with $MgSO_4$, filtered, and filtrate concentrated *in vacuo* at 50 °C to afford (1R,5S,6s)-3-(*tert*-butoxycarbonyl)-3-azabicyclo[3.1.0]hexane-6-carboxylic acid (*endo*-**11**) (101 mg, 444 µmol, 88.9% yield, *exo*:*endo* d.r. <1:30) as a white solid with a hint of pink-hue.

^{1}H NMR (400 MHz, $CDCl_3$) δ 3.76 – 3.73 (d, J = 11.4 Hz, 2H), 3.52 – 3.48 (dt, J = 11.4, 2.2 Hz, 2H), 2.0 – 1.94 (ddd, J = 8.1, 3.0, 1.4 Hz, 2H), 1.80 – 1.76 (t, J = 8.1 Hz, 1H), 1.43 (s, 9H).

^{13}C NMR (101 MHz, $CDCl_3$) δ 174.5, 154.3, 79.5, 45.6, 28.5, 23.2, 22.5.

HRMS (-p APCI): calc. mass for $C_{11}H_{16}O_4N$ [M – H]$^-$ - 226.1085; obs. mass for $C_{11}H_{16}O_4N$ [M – H]$^-$ - 226.1085.

Melting Point (°C): 135 – 137 °C.

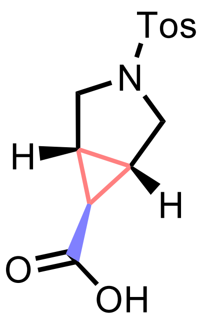

(1R,5S,6r)-3-tosyl-3-azabicyclo[3.1.0]hexane-6-carboxylic acid (*exo*-15)

From **General Procedure C**, **strategy iii**. To a flame-dried 50 mL round-bottom flask charged with an octagonal stir-bar (1 in x 5/16 in) and 10 g activated 4 Å MS was added 1-tosyl-2,5-dihydro-1H-pyrrole (**12**) (4.466 g, 2 equiv, 20.00 mmol) to the vessel. The vessel was backfilled with nitrogen three times and then capped with a nitrogen balloon. DMC (17.57 mL) was added to the reaction vessel. Subsequently, a stock solution of $Rh(II)_2(esp)_2$ in CH_2Cl_2 (379.2 µg, 1.000 mL, 0.00050 molar, .00005 equiv, 0.5000 µmol) was added to the vessel. The reaction vessel containing trap and catalyst was stirred at 300 rpm at 90 ^{o}C for at least 10 minutes. After the elapsed time, to the reaction solution was added ethyl 2-diazoacetate (**10**) in CH_2Cl_2 (1.542 g, 1.421 mL, 74% Wt, 1 equiv, 10.00 mmol) to the reaction vessel via a dual-syringe pump [dual-syringe pump settings -- 3 mL syringe, 0.475 mL/hr, diameter 9.8 mm, vol 1.425 mL; Air-Tite/SilverPoint 22Gx4" long hypodermic needle was used] over 3 h. The needle was submerged into the reaction solution. After complete addition and elapsed time of 3 h, the resulting solution was reacted for an additional 2.5 h and then subsequently, cooled to room temperature, filtered over a plug of celite, and the filtrate concentrated *in vacuo* over 50 ^{o}C in a 250 mL round-bottom flask. The crude material was then subjected to the isomerization conditions.

To a 250 mL round-bottom flask charged with a stir-bar and the crude was added 2 M sodium *tert*-butoxide in THF (2.883 g, 15.00 mL, 2 molar, 3 equiv, 30.00 mmol). An immediate color change to brown-orange was observed with addition of base. The reaction mixture was allowed to stir for at least 10 minutes at room temperature to ensure complete isomerization of the starting material. To the reaction mixture was then added ethanol (39.39 g, 49.9 mL, 85.5 equiv, 855.0 mmol) and then 2 M sodium hydroxide in deionized water (4.000 g, 50.00 mL, 2 molar, 10 equiv, 100.0 mmol). The reaction mixture was allowed to stir at 25 °C for 46 h. After the elapsed time, the suspension was vacuum filtered through a fritted funnel, rinsing the filter cake with deionized water, which resulted in an orange filtrate and a dried filter-cake (pure **12** based on ^{1}H NMR analysis). The orange filtrate was concentrated *in vacuo* at 55 ^{o}C to get rid of any organic volatiles. The basic aqueous suspension (pH 14) was diluted with deionized water and heated to 75 ^{o}C via a water bath to warm the suspension for at least 10 min (some *exo*-**15** was observed to precipitate out at room temperature; heating to 75 ^{o}C ensures all *exo*-**15** solubilizes in the aqueous solution while **12** is insoluble in the heated solution). The hot suspension was then vacuum filtered through a fritted funnel rinsing with warm deionized water resulting in a yellow filtrate and a dried filter-cake (pure **12** based on ^{1}H NMR analysis). The combined filter-cake resulted in an approximately recovered 2.88 g of clean **12** as a white powder, which equates to approximately 0.71 equiv of the 2 equiv of **12** had been consumed during the cyclopropanation step. The basic yellow filtrate was placed into a saltwater ice bath, cooled, and slowly acidified with 1 M HCl to a pH of 1 resulting in a white precipitate in the yellow aqueous solution. Subsequently, the aqueous suspension was extracted with ethyl acetate five times, the organic layer dried with $MgSO_4$, filtered through celite, and the filtrate concentrated *in vacuo* at 55 ^{o}C resulting in an off-white solid. The off-white solid was resuspended in *n*-heptane, sonicated, the suspension filtered through vacuum filtration using a slow flowrate, fine porosity filter paper and funnel. The filter cake was washed with *n*-heptane, triturated, and dried under vacuum to afford (1R,5S,6r)-3-tosyl-3-azabicyclo[3.1.0]hexane-6-

carboxylic acid (*exo*-**15**) (1.634 g, 5.808 mmol, 58.08 % yield *over 3 steps*, *exo*:*endo* d.r. >30:1) as a light tan fluffy powder.

^{1}H NMR (400 MHz, $CDCl_3$) δ 7.68 – 7.66 (d, J = 8.3 Hz, 2H), 7.35 – 7.33 (d, J = 7.7 Hz, 2H), 3.64 – 3.61 (d, J = 9.8 Hz, 2H), 3.15 – 3.09 (d, J = 9.6 Hz, 2H), 2.44 (s, 3H), 2.06 – 2.02 (m, 2H), 1.71 – 1.69 (t, J = 3.0 Hz, 1H).

^{13}C NMR (101 MHz, $CDCl_3$) δ 177.8, 143.9, 133.0, 129.8, 127.6, 49.3, 26.4, 22.4, 21.6.

HRMS (-p APCI): calc. mass for $C_{13}H_{14}O_4N^{32}S$ [M – H]$^-$ - 280.0649; obs. mass for $C_{13}H_{14}O_4N^{32}S$ [M – H]- - 280.0645.

Melting Point (°C): 203 – 205 °C.

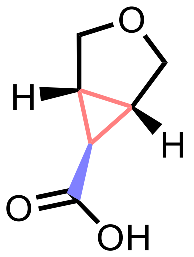

(1R,5S,6r)-3-oxabicyclo[3.1.0]hexane-6-carboxylic acid (*exo*-16)

From **General Procedure C**, **strategy iii**. To a flame-dried 50 mL round-bottom flask was charged with an octagonal stir-bar (1 in x 5/16 in) and 10 g of activated 4 Å MS. The vessel was backfilled with nitrogen three times and then capped with a nitrogen balloon. DMC (17.57 mL) and then nitrogen sparged 2,5-dihydrofuran (**13**) (1.402 g, 1.482 mL, 2 equiv, 20.00 mmol) was added to the vessel. Subsequently, a stock solution of $Rh(II)_2(esp)_2$ in CH_2Cl_2 (379.2 µg, 1.000 mL, 0.00050 molar, .00005 equiv, 0.5000 µmol) was added to the vessel. The reaction vessel containing trap and catalyst was stirred at 300 rpm at 70 °C for at least 10 minutes. After the elapsed time, to the reaction solution was added ethyl 2-diazoacetate (**10**) in CH_2Cl_2 (1.542 g, 1.421 mL, 74% Wt, 1 equiv, 10.00 mmol) to the reaction vessel via a dual-syringe pump [dual-syringe pump settings -- 3 mL syringe, 0.475 mL/hr, diameter 9.8 mm, vol 1.425 mL; Air-Tite/SilverPoint 22Gx4" long hypodermic needle was used] over 3 h. The needle was submerged into the reaction solution. After complete addition and elapsed time of 3 h, the resulting solution was reacted for an additional 2.5 h and then subsequently, cooled to room temperature, filtered over a plug of celite, and the filtrate concentrated *in vacuo* over 50 °C in a 250 mL round-bottom flask. The crude material was then subjected to the isomerization conditions.

To the 250 mL round-bottom flask charged with a stir-bar and the crude was added 2 M sodium *tert*-butoxide in THF (2.883 g, 15.00 mL, 2 molar, 3 equiv, 30.00 mmol). An immediate color change to brown-orange was observed with addition of base. The reaction mixture was allowed to stir for at least 10 minutes at room temperature to ensure complete isomerization of starting material. To the reaction mixture was then added ethanol (39.39 g, 49.9 mL, 85.5 equiv, 855.0 mmol) and then 2 M sodium hydroxide in deionized water (4.000 g, 50.00 mL, 2 molar, 10 equiv, 100.0 mmol). The reaction mixture was allowed to stir at 25 °C for 24 h. After the elapsed time, the mixture was concentrated *in vacuo* at 55 °C to get rid of any organic volatiles. To the concentrated aqueous solution was washed with diethyl ether five times. Afterwards, the aqueous solution was placed in a saltwater ice bath, cooled, and slowly acidified with 1 M HCl to a pH of 1 – 2 (color change from dark brown to red-orange when pH 1 – 2). Subsequently, the aqueous solution was diluted with ethyl acetate, extracted with ethyl acetate three times, the organic layer dried with $MgSO_4$, filtered, and the filtrate concentrated *in vacuo* at 55 °C to afford

a colored solid. The material was resuspended in hexanes with a minimal amount of diethyl ether. The suspension was vacuum filtered and the filter cake dried to afford (1R,5S,6r)-3-oxabicyclo[3.1.0]hexane-6-carboxylic acid (*exo*-**16**) (917 mg, 7.16 mmol, 71.6% yield *over 3 steps*, *exo*:*endo* d.r. >30:1) as a burgundy crystalline solid.

^{1}H NMR (400 MHz, $CDCl_3$) δ 3.96 – 3.94 (d, J = 8.8 Hz, 2H), 3.79 – 3.73 (m, 2H), 2.25 – 2.20 (m, 2H), 1.62 – 1.60 (t, J = 3.2 Hz, 1H).

^{13}C NMR (101 MHz, $CDCl_3$) δ 179.0, 69.1, 27.8, 21.9.

HRMS (-p APCI): calc. mass for $C_6H_7O_3$ $[M – H]^-$ - 127.0401; obs. mass for $C_6H_7O_3$ $[M – H]^-$ - 127.0405.

Melting Point (°C): 112 – 114 °C.

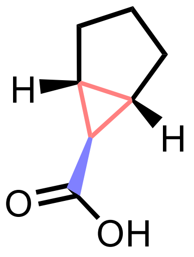

(1R,5S,6r)-bicyclo[3.1.0]hexane-6-carboxylic acid (*exo*-17)

From **General Procedure C**, **strategy iii**. To a flame-dried 50 mL round-bottom flask was charged with an octagonal stir-bar (1 in x 5/16 in) and 10 g of activated 4 Å MS. The vessel was backfilled with nitrogen three times and then capped with a nitrogen balloon. DMC (17.57 mL) and then nitrogen sparged cyclopentene (**14**) (1.362 g, 1.77 mL, 2 equiv, 20.00 mmol) was added to the vessel. Subsequently, a stock solution of $Rh(II)_2(esp)_2$ in CH_2Cl_2 (379.2 µg, 1.000 mL, 0.00050 molar, .00005 equiv, 0.5000 µmol) was added to the vessel. The reaction vessel containing trap and catalyst was stirred at 300 rpm at 50 °C for at least 10 minutes. After the elapsed time, to the reaction solution was added ethyl 2-diazoacetate (**10**) in CH_2Cl_2 (1.542 g, 1.421 mL, 74% Wt, 1 equiv, 10.00 mmol) to the reaction vessel via a dual-syringe pump [dual-syringe pump settings -- 3 mL syringe, 0.475 mL/hr, diameter 9.8 mm, vol 1.425 mL; Air-Tite/SilverPoint 22Gx4" long hypodermic needle was used] over 3 h. The needle was submerged into the reaction solution. After complete addition and elapsed time of 3 h, the resulting solution was reacted for an additional 2.5 h and then subsequently, cooled to room temperature, filtered over a plug of celite, and the filtrate concentrated *in vacuo* over 50 °C in a 250 mL round-bottom flask. The crude material was then subjected to the isomerization conditions.

To the 250 mL round-bottom flask charged with a stir-bar and the crude was added 2 M sodium *tert*-butoxide in THF (2.883 g, 15.00 mL, 2 molar, 3 equiv, 30.00 mmol). An immediate color change to brown-orange was observed with addition of base. The reaction mixture was allowed to stir for at least 10 minutes at room temperature to ensure complete isomerization of starting material. To the reaction mixture was then added ethanol (39.39 g, 49.9 mL, 85.5 equiv, 855.0 mmol) and then 2 M sodium hydroxide in deionized water (4.000 g, 50.00 mL, 2 molar, 10 equiv, 100.0 mmol). The reaction mixture was allowed to stir at 25 °C for 24 h. After the elapsed time, the mixture was concentrated *in vacuo* at 55 °C to get rid of any organic volatiles. To the concentrated aqueous solution was washed with diethyl ether five times. Afterwards, the aqueous solution was placed in a saltwater ice bath, cooled, and acidified with 1 M HCl to a pH of 1 – 2. Subsequently, the aqueous solution was diluted with ethyl acetate, extracted with ethyl acetate three times, the organic layer dried with $MgSO_4$, filtered, and the filtrate concentrated *in vacuo* at 55 °C to afford (1R,5S,6r)-bicyclo[3.1.0]hexane-6-carboxylic acid (*exo*-**17**) (857 mg, 6.79 mmol,

67.9% yield *over 3 steps*, *exo*:*endo* d.r. >30:1) as a red orange viscous oil that solidifies over time into a brown crystalline solid.

^{1}H NMR (400 MHz, $CDCl_3$) δ 11.20 (brs, 1H), 1.95 – 1.91 (d, J = 3.0 Hz, 2H), 1.88 – 1.72 (m, 4H), 1.64 – 1.57 (dt, J = 13.4, 8.2 Hz, 1H), 1.39 – 1.38 (t, J = 2.9 Hz, 1H), 1.15 – 1.01 (dtt, J = 13.4, 11.1, 8.2 Hz, 1H).

^{13}C NMR (101 MHz, $CDCl_3$) δ 180.9, 29.8, 27.2, 21.2, 20.0.

HRMS (-p APCI): calc. mass for $C_7H_9O_2$ $[M – H]^-$ - 125.0608; obs. mass for $C_7H_9O_2$ $[M – H]^-$ - 125.0612.

Melting Point (^{o}C): 43 – 45 ^{o}C.

9. ReactIR Experimentation – Diazo Accumulation Study

Warning: This project involves the use of diazo compounds. Diazo compounds are known to have thermal stability issues and are explosive hazards; work with diazo compounds should be performed in a well-ventilated hood, require the use of PPE, and careful handling of the reagents.[1]

Reaction setup and analysis was adapted from literature precedent.[12] The ReactIR instrument was filled with liquid nitrogen and allowed to equilibrate while the reaction flask was being set-up. An oven-dried 50 mL three-neck round-bottom flask with 10.0 g of activated 4 Å MS was fitted with a rubber septum (left neck, 14/20), ReactIR probe (center neck, 24/40 to 19/22 adapter, 19/22 neck), and nitrogen inlet (right neck, 14/20). The flask was cooled to room temperature *in vacuo*, then backfilled with nitrogen and placed in a oil bath, with the temperature of the stir plate set to 90 ^{o}C and stir rate on 150 rpm. Once the reaction flask was at the desired temperature, the background and water vapor spectrum were taken via iC IR 4.3 software. The syringe and needle used for the solvent was primed with argon before adding 17.57 mL dry, distilled, N_2-sparged DMC through the rubber septum of the vessel. The stirring was started here and the ReactIR probe was made sure to not touch the activated 4 Å MS bead. The data collection was started on iC IR 4.3, and the solvent was allowed to stir for at least 5 min. After a reference spectrum of the solvent was taken, the septum from the left neck from removed and *tert*-butyl 2,5-dihydro-1H-pyrrole-1-carboxylate (**9**) (3.384 g, 2 equiv, 20.00 mmol) was quickly added to the reaction vessel and then recapped with the septum. The reaction mixture was allowed to stir for at least 5 min. A reference spectrum of *tert*-butyl 2,5-dihydro-1H-pyrrole-1-carboxylate (**9**) was taken after subtracting out the solvent spectrum, and then a stock solution of $Rh(II)_2(esp)_2$ in CH_2Cl_2 (379.2 µg, 1.000 mL, 0.00050 molar, .00005 equiv, 0.5000 µmol) was added. A reference spectrum of the catalyst stock solution was taken after subtracting out the reference spectrum of *tert*-butyl 2,5-dihydro-1H-pyrrole-1-carboxylate (**9**), and the reaction mixture was allowed to stir for at least 5 min. Ethyl 2-diazoacetate (**10**) (1.542 g, 1.421 mL, 74% Wt, 1 equiv, 10.00 mmol) was added to the reaction mixture via a dual-syringe pump [dual-syringe pump settings -- 3 mL syringe, 0.475 mL/hr, diameter 9.8 mm, vol 1.425 mL; Air-Tite/SilverPoint 22Gx4" long hypodermic needle was used] over 3 h; $C=N_2$ stretch frequencies were measured over the course of the reaction (peak heights from 2112 and 2108 cm^{-1}; peak baseline range 2300 to 2000 cm^{-1} to take into account of dilute diazo sample). After complete addition and elapsed time of 3 h, the resulting solution was reacted for an additional 2.5 h and then subsequently, cooled and filtered over celite and the filtrate concentrated *in vacuo* over 50 ^{o}C into a tared vial and the crude mass was measured. A known amount of 1,3,5-trimethoxybenzene was added to the vial, dissolved in $CDCl_3$, and then a ^{1}H NMR was acquired.

$$P_{sample} = (I_{Analyte} / I_{CRM}) \times (N_{CRM} / N_{Analyte}) \times (M_{Analyte} / M_{CRM}) \times (m_{CRM} / m_{Sample}) \times (P_{CRM})$$

P_{sample} = (6.92 / 1.00) x (3H / 2H) x (255.31 g/mol / 168.07 g/mol) x (136.1 mg of trimethoxybenzene / 4172.9 mg of crude) x (99% purity of trimethoxybenzene) = **50.9% purity** for 3-(*tert*-butyl) 6-ethyl 3-azabicyclo[3.1.0]hexane-3,6-dicarboxylate (*exo*/*endo*-**1**)

qNMR yield = [(4.1729 g of crude) x (50.9% purity for 3-(*tert*-butyl) 6-ethyl 3-azabicyclo[3.1.0]hexane-3,6-dicarboxylate / 100%)] x (1 / 2.553 g theoretical mass) x (100%) = **83.2% qNMR yield** of 3-(*tert*-butyl) 6-ethyl 3-azabicyclo[3.1.0]hexane-3,6-dicarboxylate (*exo*/*endo*-**1**)

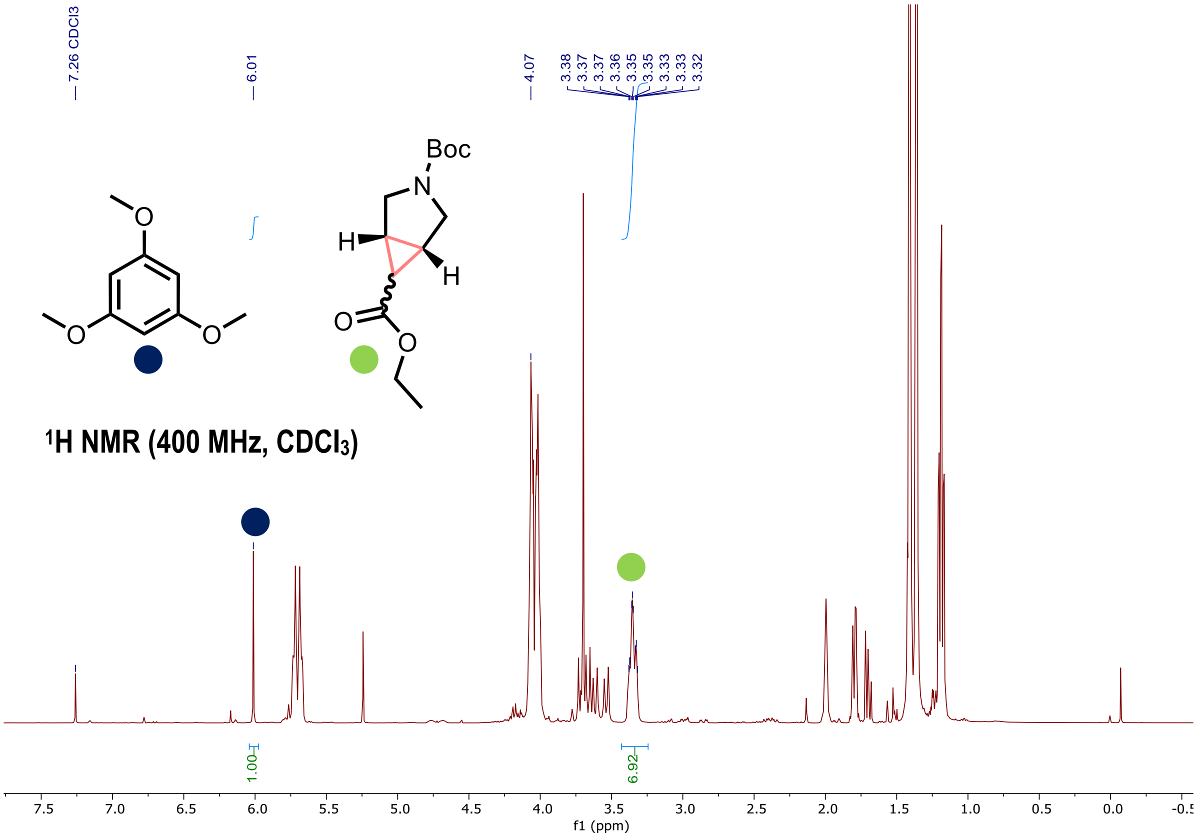

Figure S6. ^{1}H NMR spectrum of crude material with internal standard at 10.0 mmol scale ReactIR study. Number of scans was set to 64 and relaxation delay was set to 5 s. Integrations at 6.01 ppm (s, aromatic 3H) of 1,3,5-trimethoxybenzene and at 3.38 – 3.32 (m, 2H) of 3-(*tert*-butyl) 6-ethyl (1R,5S)-3-azabicyclo[3.1.0]hexane-3,6-dicarboxylate (*exo*/*endo*-**1**) were measured and used for qNMR analysis.

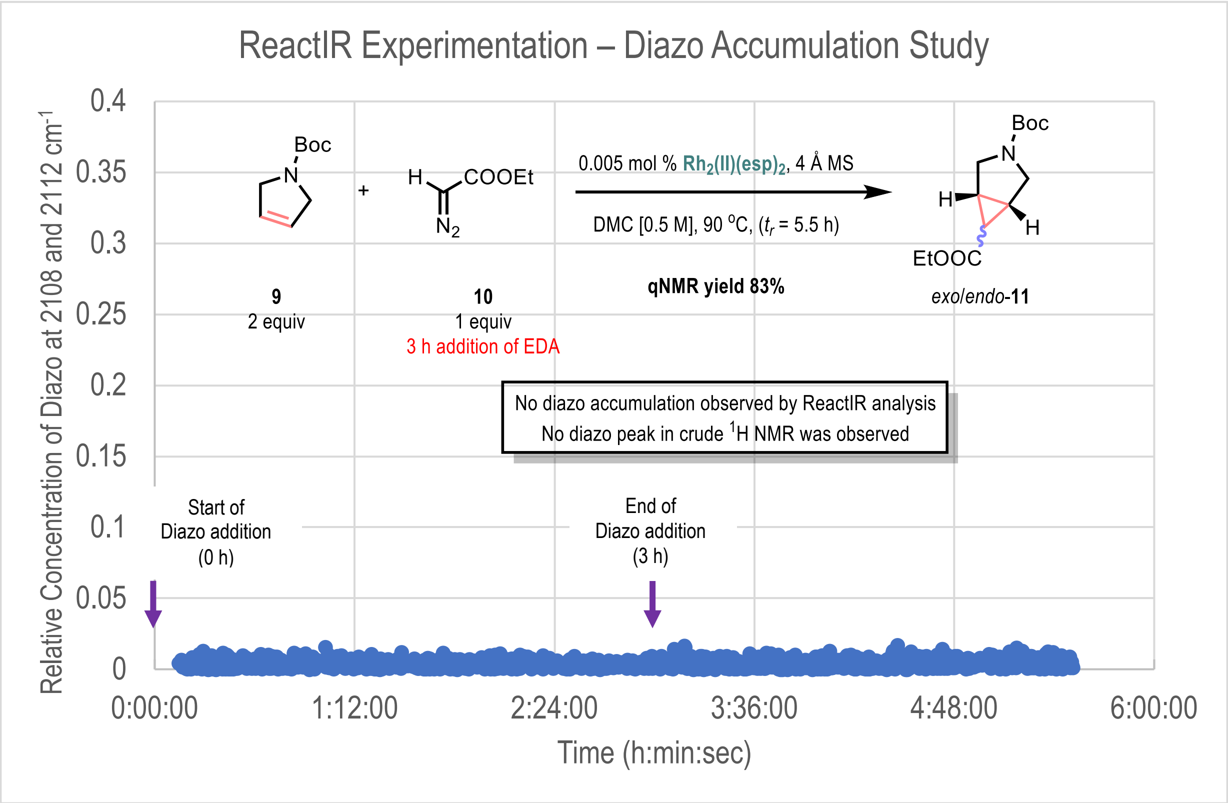

Figure S7. React IR study at 10 mmol scale on optimized conditions. Measurements were started at 0:08:47 and stopped at 5:30:57 (total 5.5 h). Diazo addition was started at 0 h and ended at 3 h. Absorbance measurements were recorded by modifying the peak parameters to Group: Height, Type: Height to Two Point Baseline, Peak 2112 to 2108 cm^{-1}, Baseline from 2300 to 2000 cm^{-1} in the iC IR 4.3 software. No significant diazo accumulation was observed due to diazo being consumed appropriately; diazo peak at 1.14 – 1.08 ppm (t, 3H) was also not found in the crude ^{1}H NMR spectrum after reaction.

10. NMR Spectra – Synthesis of Rh_2[*S*-*tetra*-(3,5-di-Br)TPPTTL]$_4$

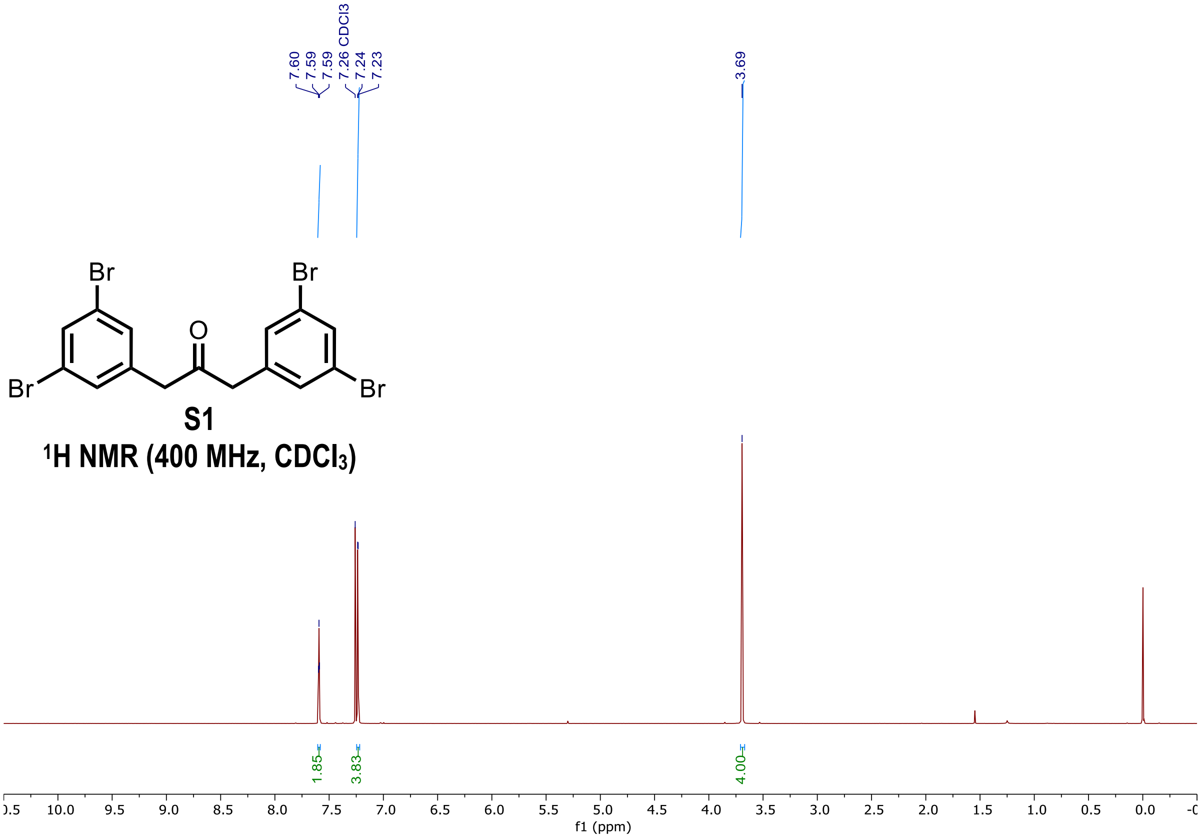

S1

^{1}H NMR (400 MHz, $CDCl_3$)

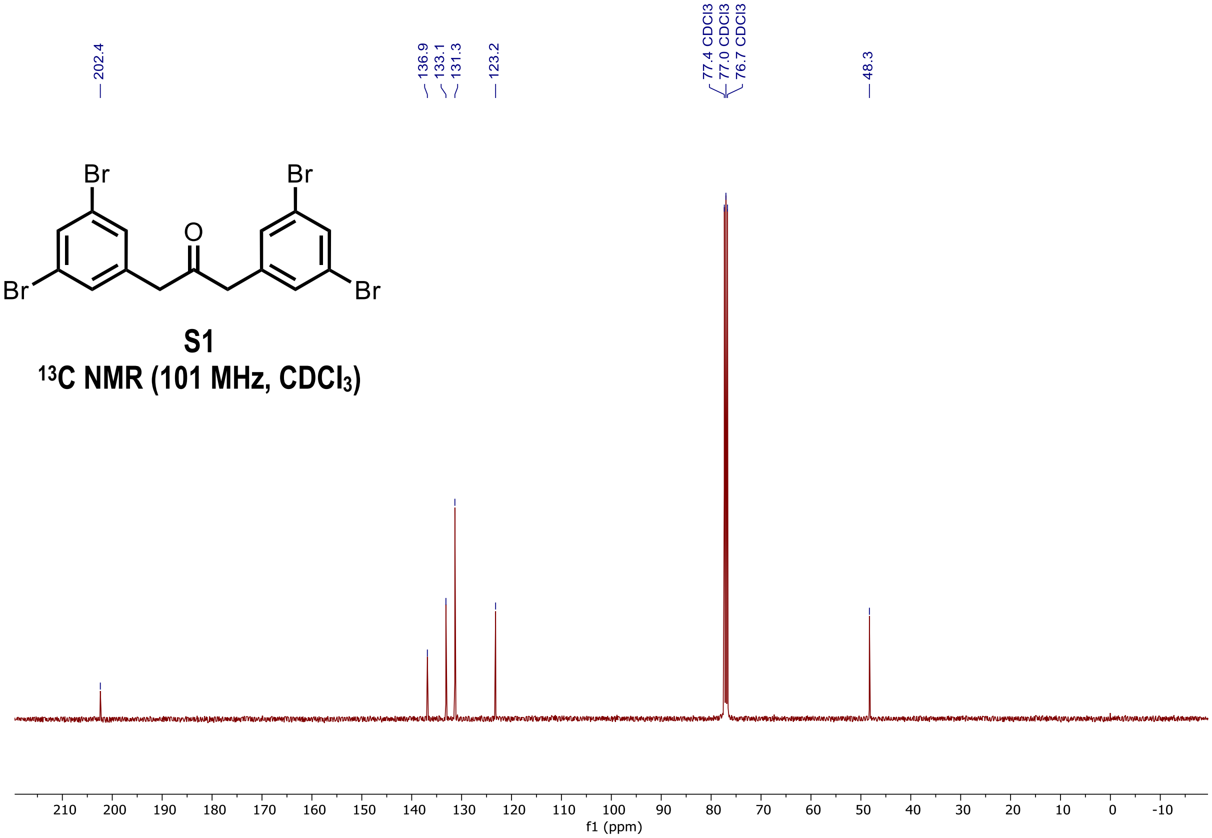

S1

^{13}C NMR (101 MHz, $CDCl_3$)

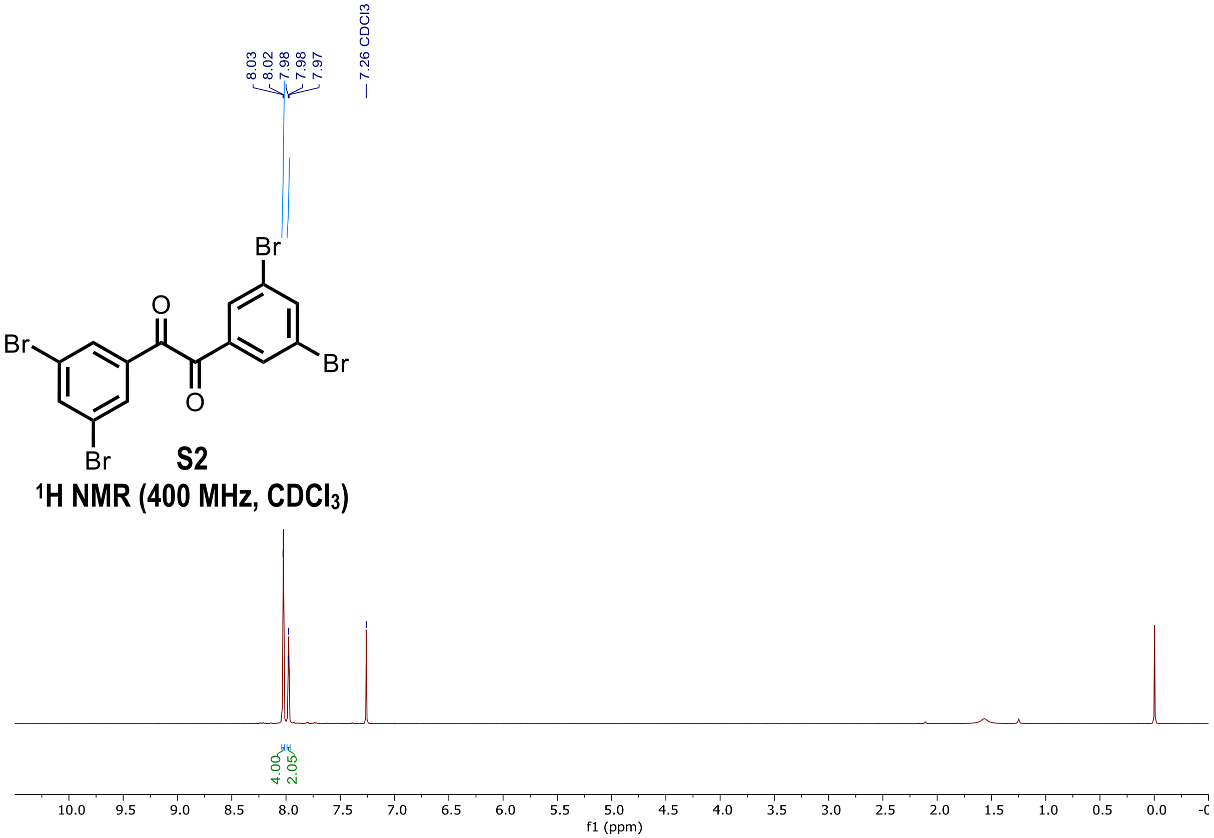
8.03
8.02
7.98
7.98
7.97
7.26 CDCl3
Br
O
Br
Br
O
Br
S2
^{1}H NMR (400 MHz, $CDCl_3$)
4.00
2.05
f1 (ppm)

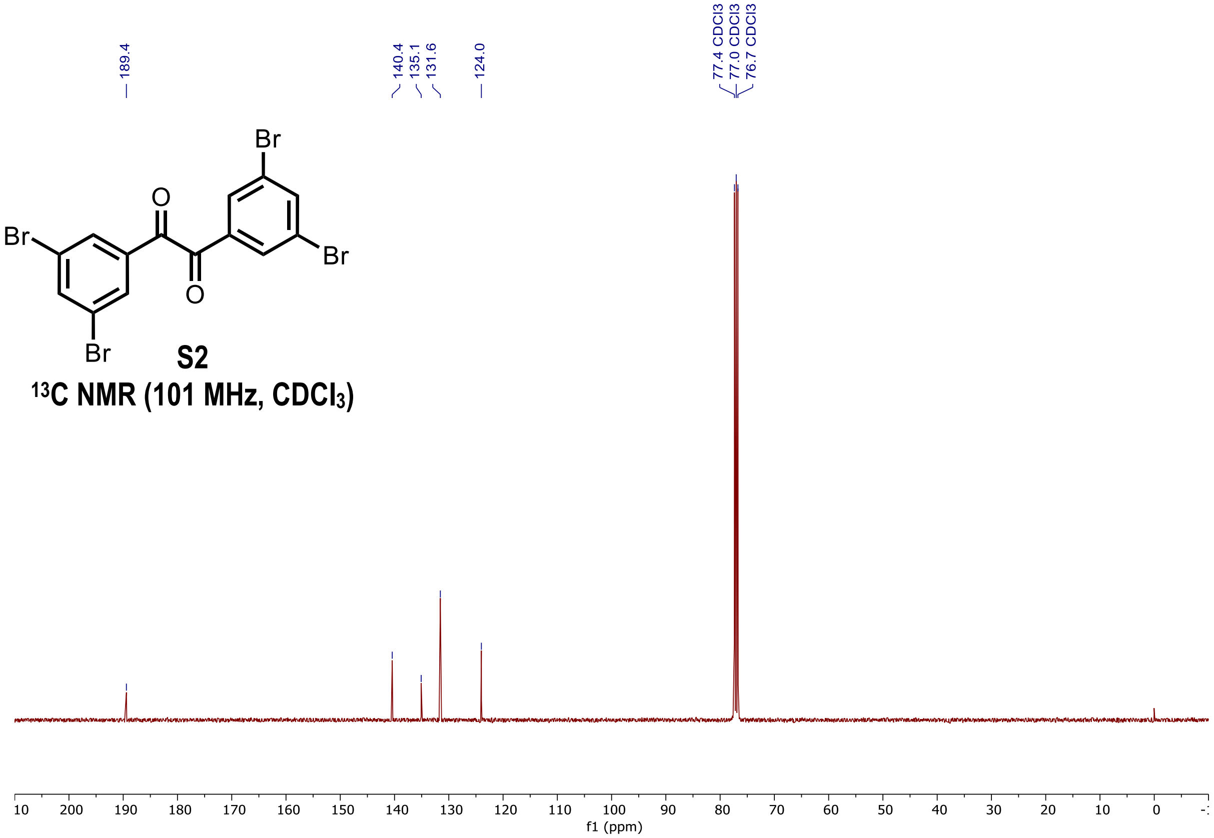
189.4
140.4
135.1
131.6
124.0
77.4 CDCl3
77.0 CDCl3
76.7 CDCl3
Br
O
Br
Br
O
Br
S2
^{13}C NMR (101 MHz, $CDCl_3$)
f1 (ppm)

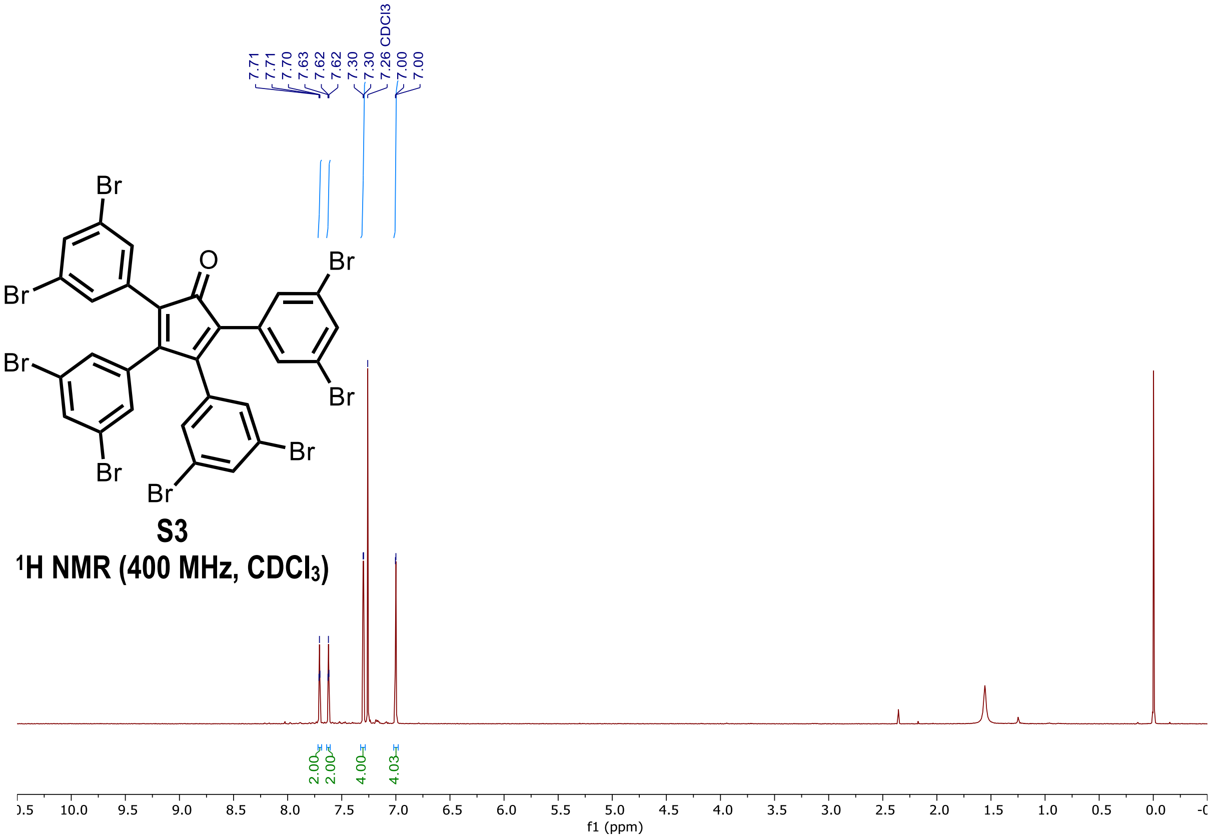

S3

^{1}H NMR (400 MHz, $CDCl_3$)

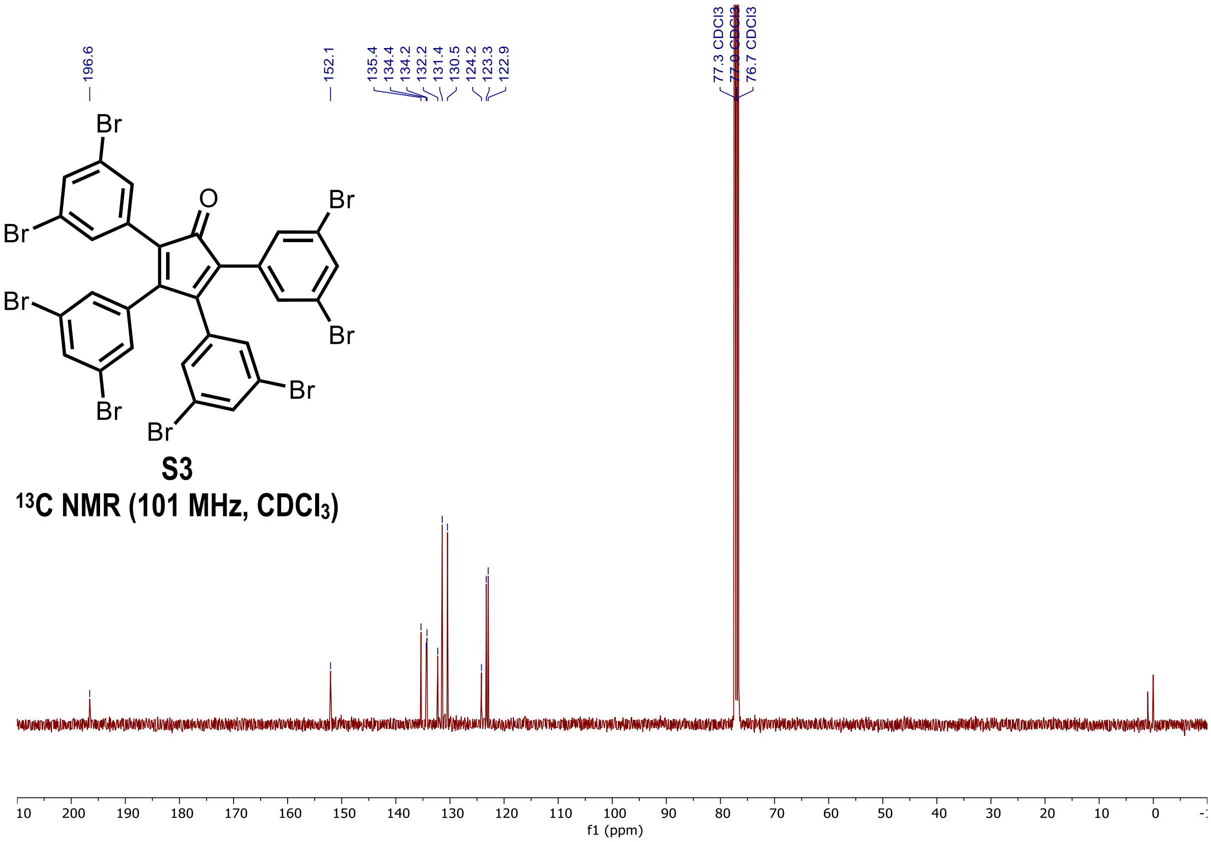

S3

^{13}C NMR (101 MHz, $CDCl_3$)

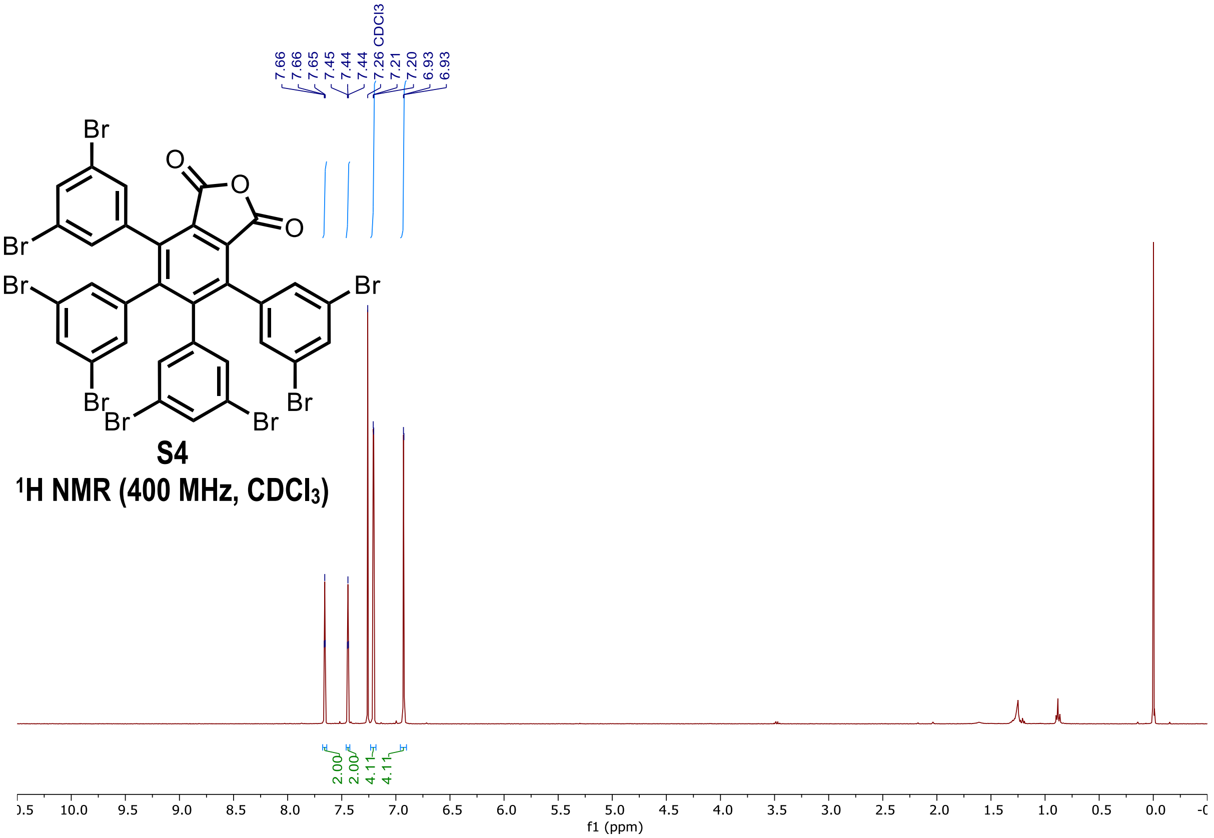
7.66
7.66
7.65
7.45
7.44
7.44
7.26 CDCl3
7.21
7.20
6.93
6.93
Br
O
O
O
S4
¹H NMR (400 MHz, CDCl₃)
2.00
2.00
4.11
4.11
f1 (ppm)

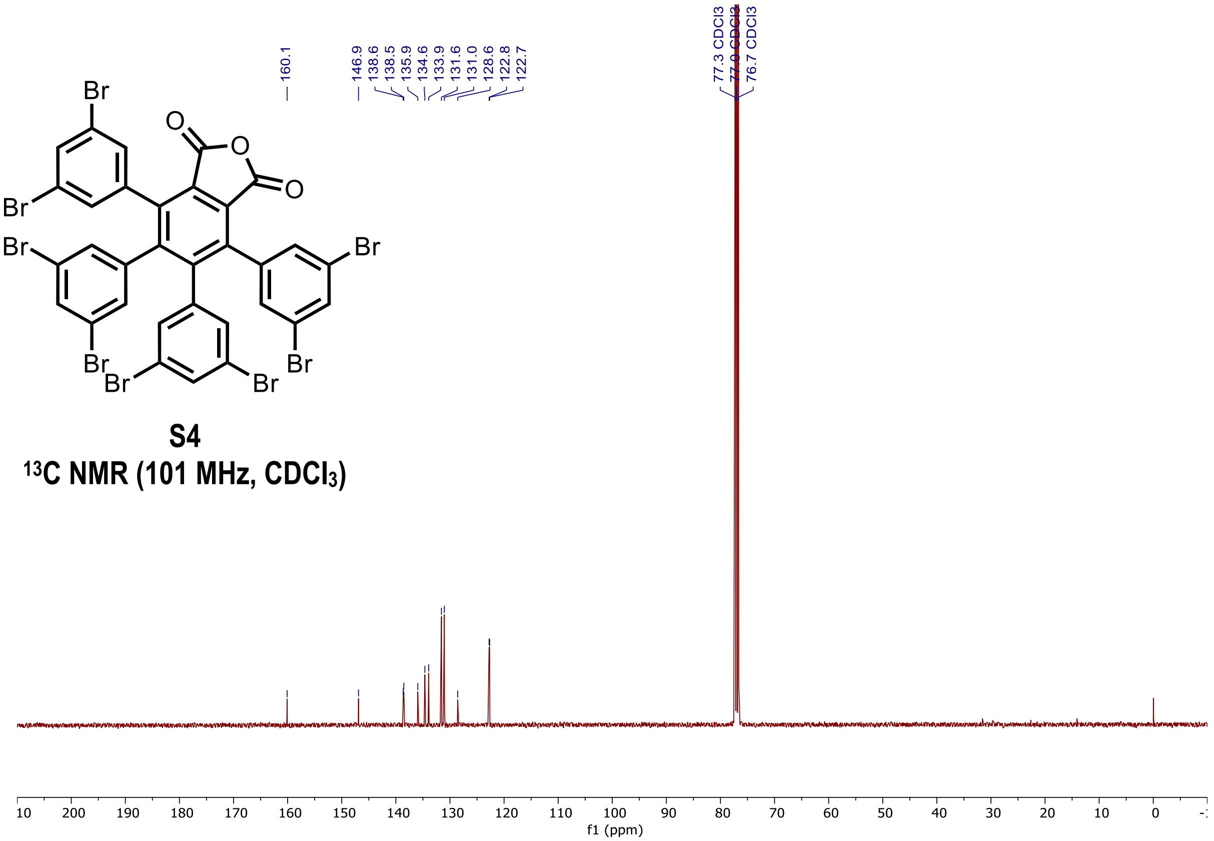
160.1
146.9
138.6
138.5
135.9
134.6
133.9
131.6
131.0
128.6
122.8
122.7
77.3 CDCl3
77.0 CDCl3
76.7 CDCl3
Br
O
O
O
S4
¹³C NMR (101 MHz, CDCl₃)
f1 (ppm)

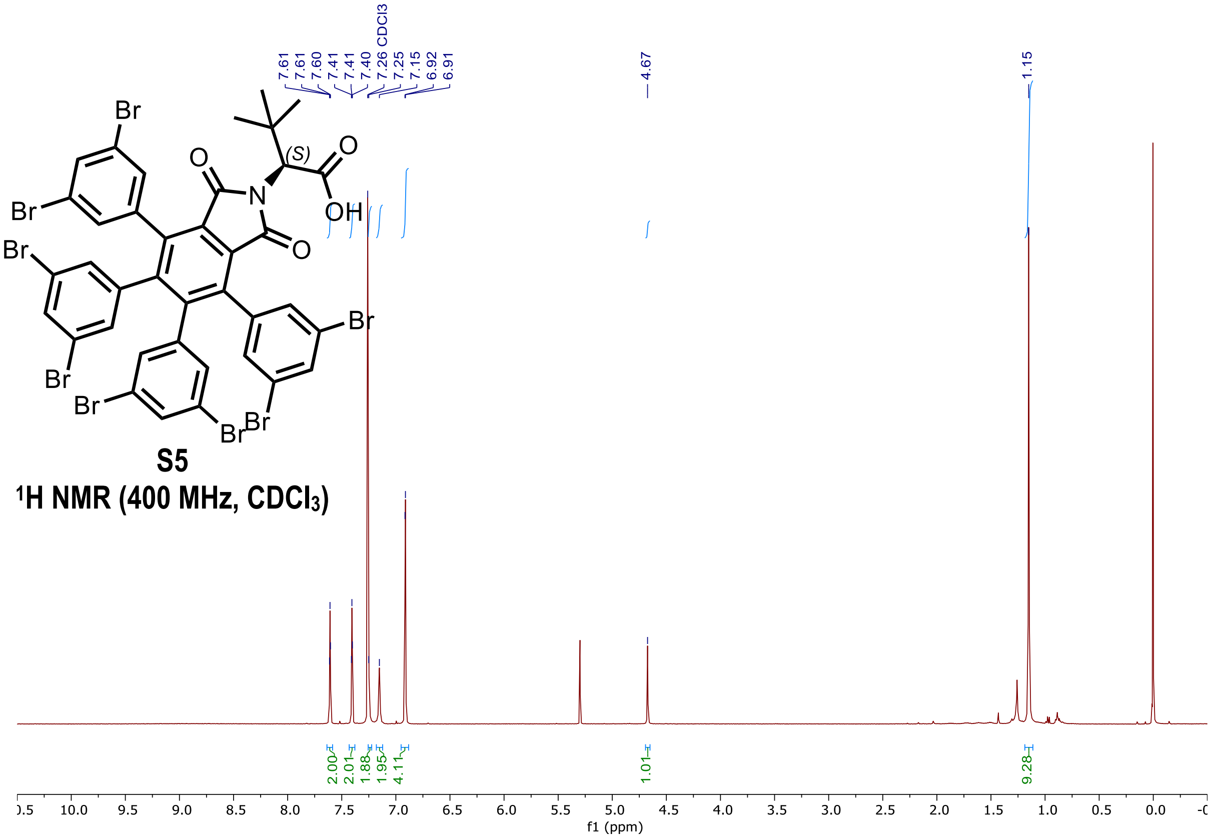

S5
1H NMR (400 MHz, CDCl3)
f1 (ppm)

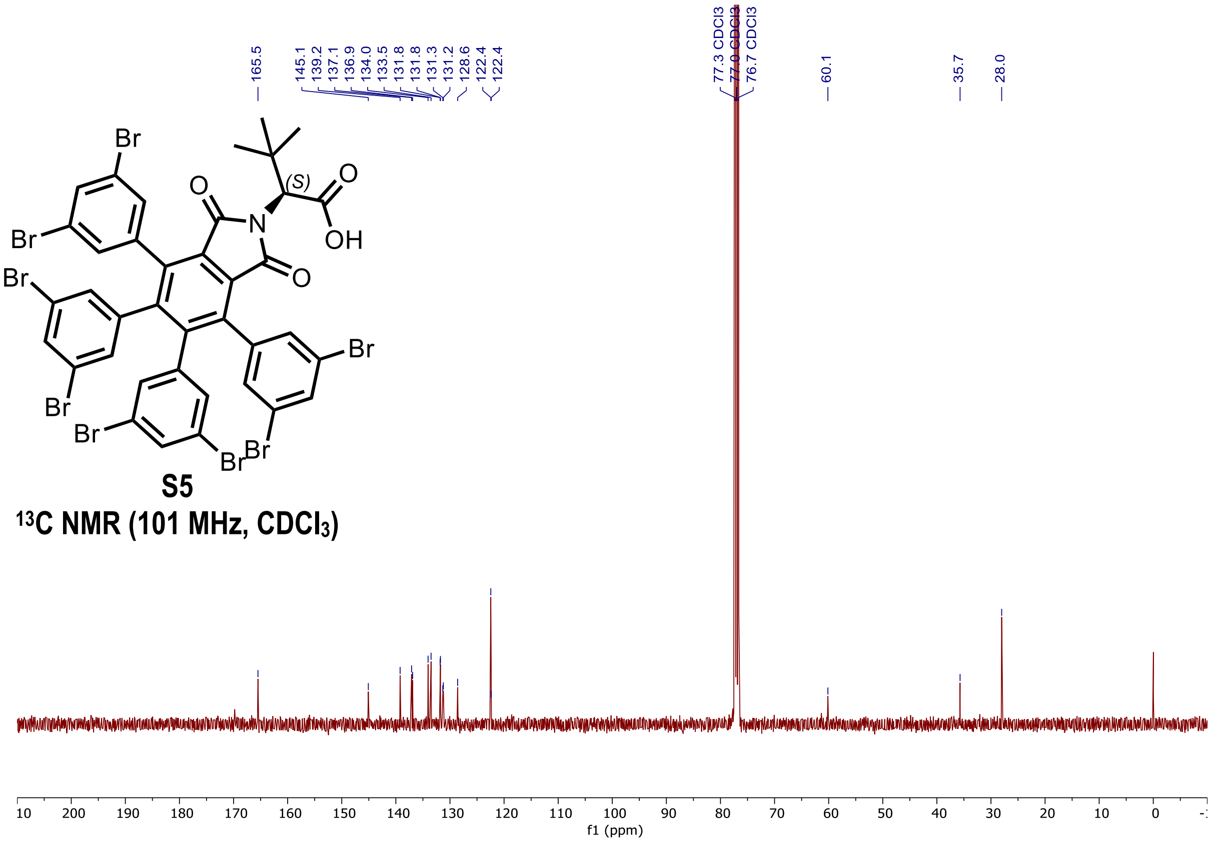

S5
13C NMR (101 MHz, CDCl3)
f1 (ppm)

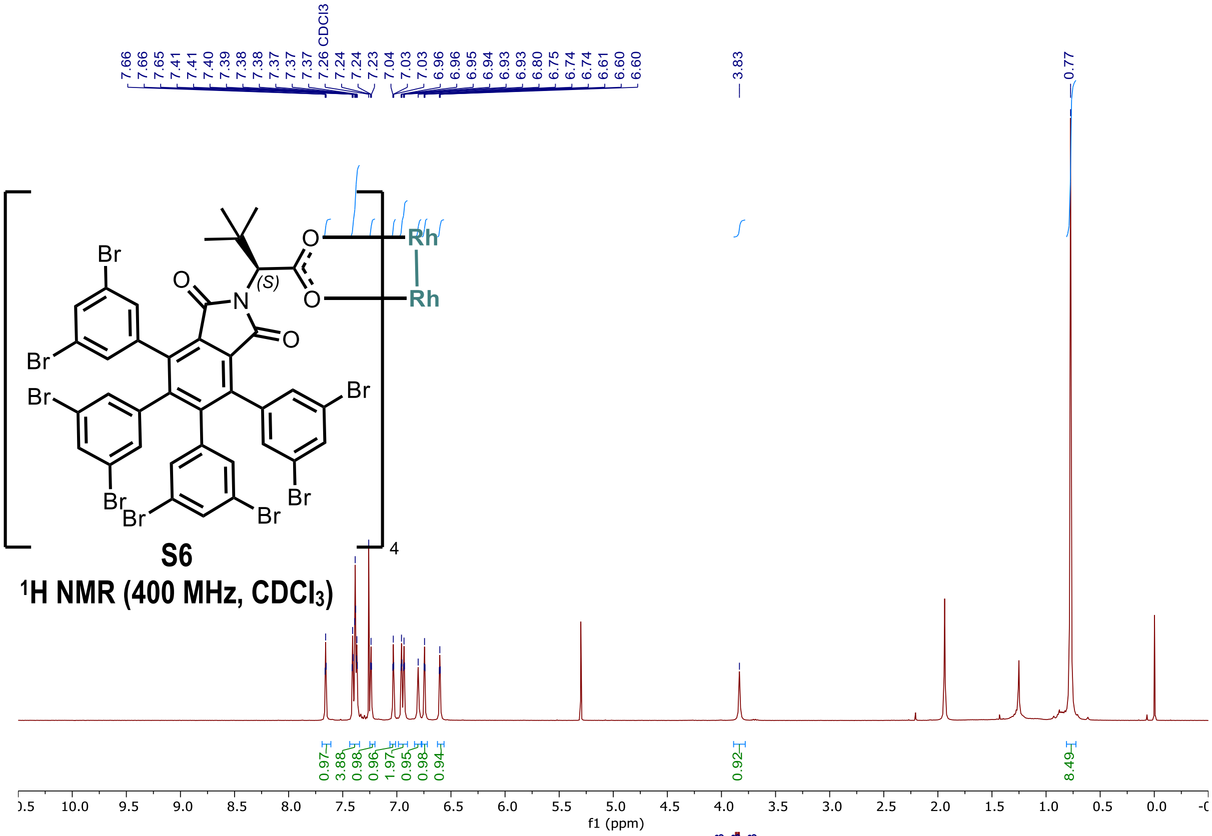
7.66
7.66
7.65
7.41
7.41
7.40
7.39
7.38
7.38
7.37
7.37
7.37
7.26 CDCl3
7.24
7.24
7.23
7.04
7.03
7.03
6.96
6.96
6.95
6.94
6.93
6.93
6.80
6.75
6.74
6.74
6.61
6.60
6.60
3.83
0.77
Br
O
(S)
N
O
Rh
Rh
S6
^{1}H NMR (400 MHz, CDCl3)
0.97
3.88
0.98
0.96
1.97
0.95
0.98
0.94
0.92
8.49
f1 (ppm)

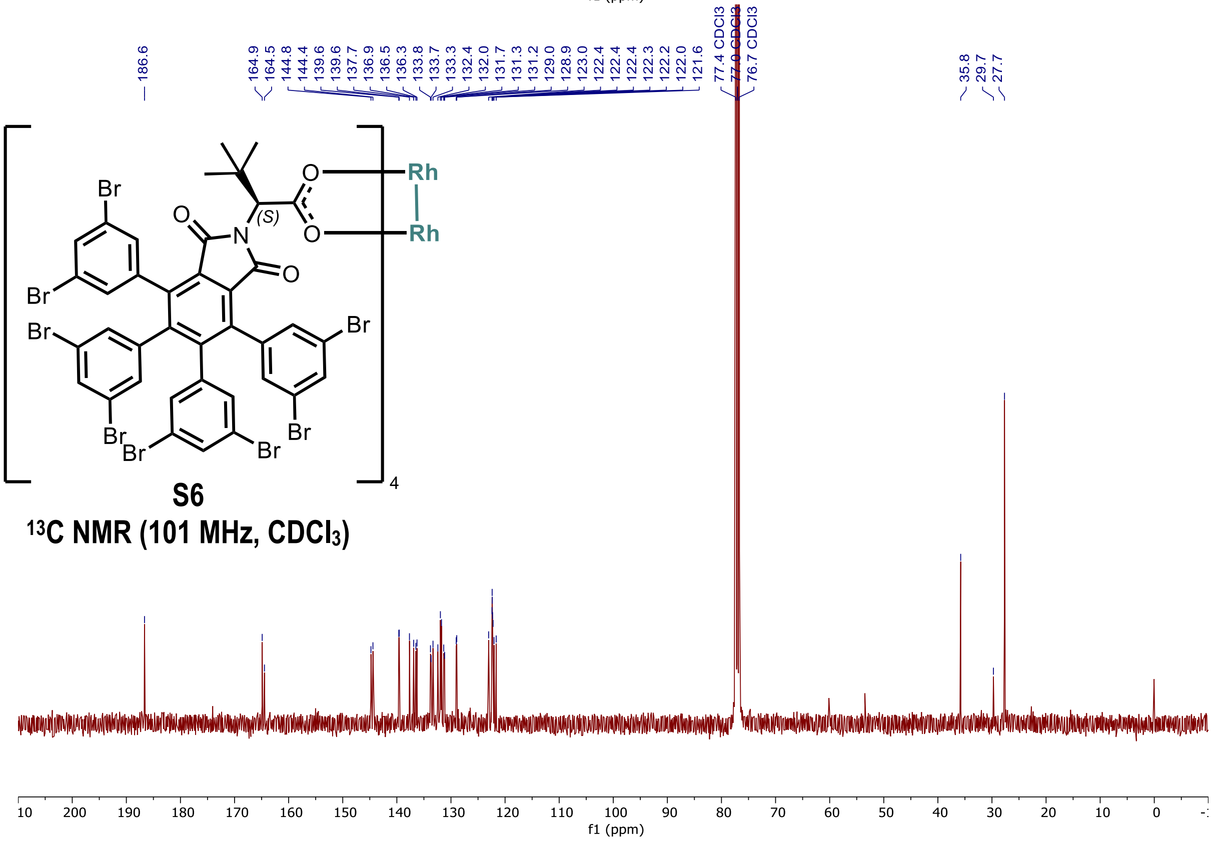
186.6
164.9
164.5
144.8
144.4
139.6
137.7
136.9
136.5
136.3
133.8
133.7
133.3
132.4
132.0
131.7
131.3
131.2
129.0
128.9
123.0
122.4
122.4
122.3
122.2
122.0
121.6
77.4 CDCl3
77.0 CDCl3
76.7 CDCl3
35.8
29.7
27.7
Br
O
(S)
N
O
Rh
Rh
S6
^{13}C NMR (101 MHz, CDCl3)
f1 (ppm)

11. NMR Spectra – *Exo*- and *Endo*-Products

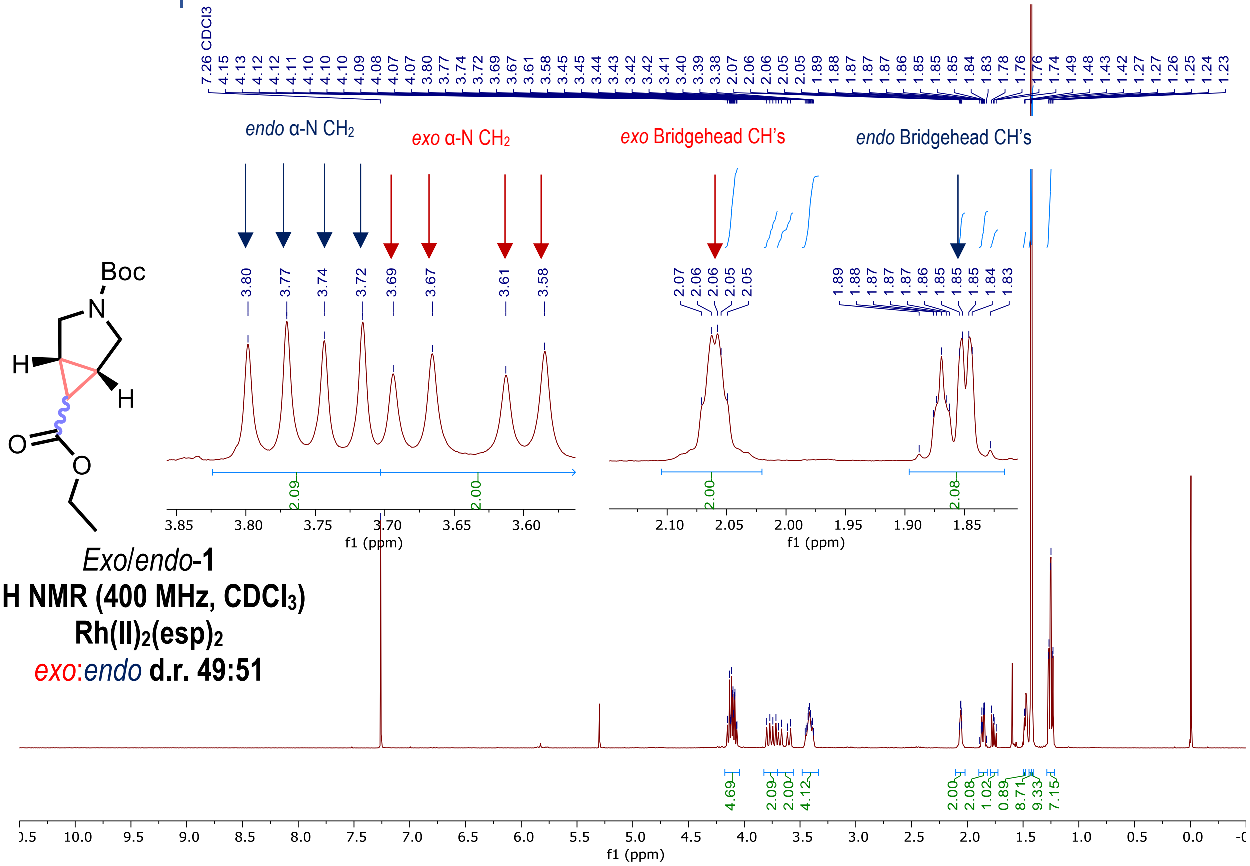

Exo/*endo*-**1**
^{1}H NMR (400 MHz, $CDCl_3$)
$Rh(II)_2(esp)_2$
exo:*endo* **d.r. 49:51**

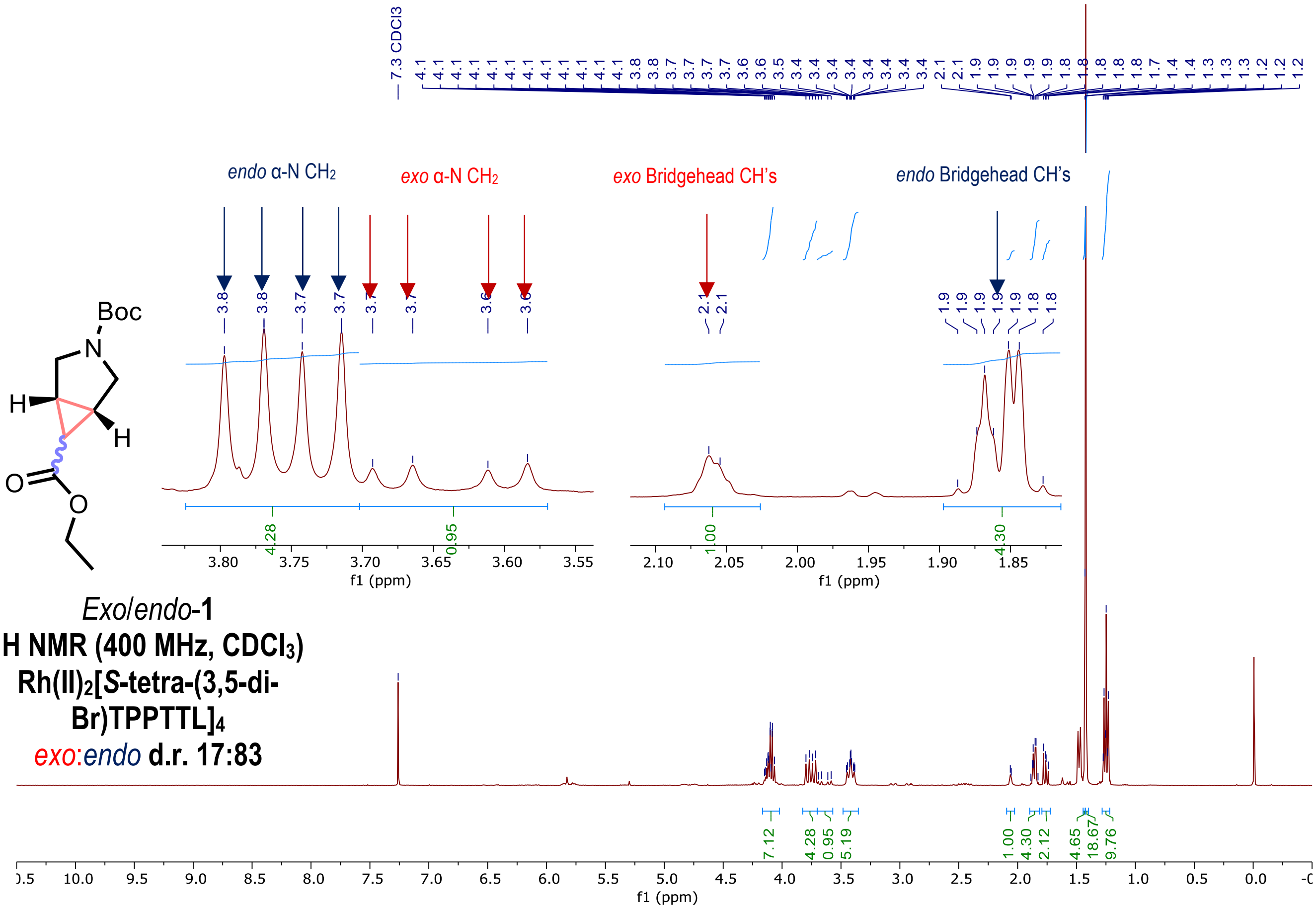

Exo/*endo*-**1**
^{1}H NMR (400 MHz, $CDCl_3$)
$Rh(II)_2$[*S*-tetra-(3,5-di-Br)TPPTTL]$_4$
exo:*endo* **d.r. 17:83**

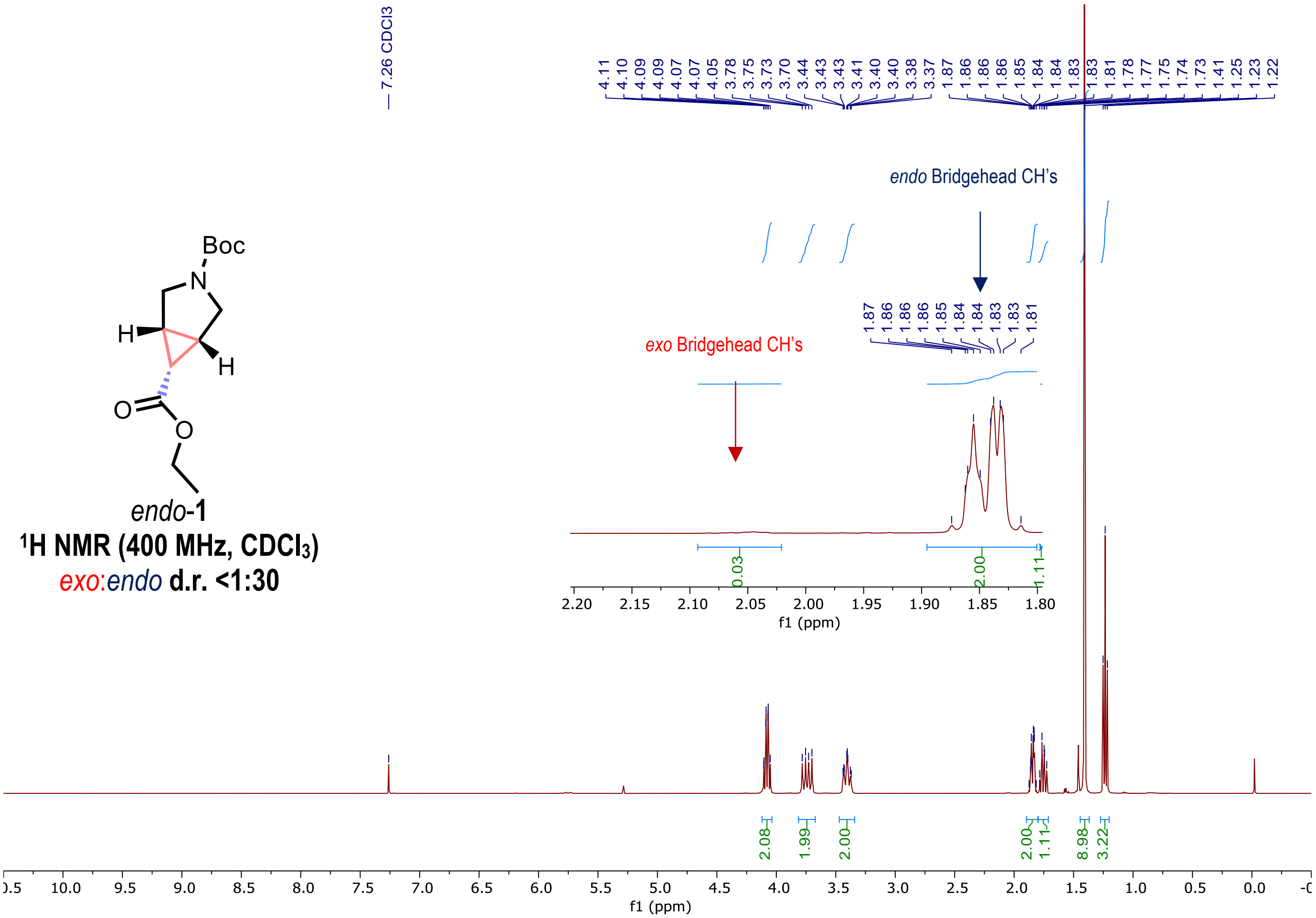
7.26 CDCl3
4.11 4.10 4.09 4.09 4.07 4.07 4.05 3.78 3.75 3.73 3.70 3.44 3.43 3.43 3.41 3.40 3.40 3.38 3.37 1.87 1.86 1.86 1.86 1.85 1.84 1.84 1.83 1.83 1.81 1.78 1.77 1.75 1.74 1.73 1.41 1.25 1.23 1.22
Boc
N
H
H
O
O
endo-1
1H NMR (400 MHz, CDCl3)
exo:endo d.r. <1:30
endo Bridgehead CH's
exo Bridgehead CH's
1.87 1.86 1.86 1.86 1.85 1.84 1.84 1.83 1.83 1.81
0.03
2.00
1.11
2.20 2.15 2.10 2.05 2.00 1.95 1.90 1.85 1.80
f1 (ppm)
2.08
1.99
2.00
2.00
1.11
8.98
3.22
10.0 9.5 9.0 8.5 8.0 7.5 7.0 6.5 6.0 5.5 5.0 4.5 4.0 3.5 3.0 2.5 2.0 1.5 1.0 0.5 0.0
f1 (ppm)

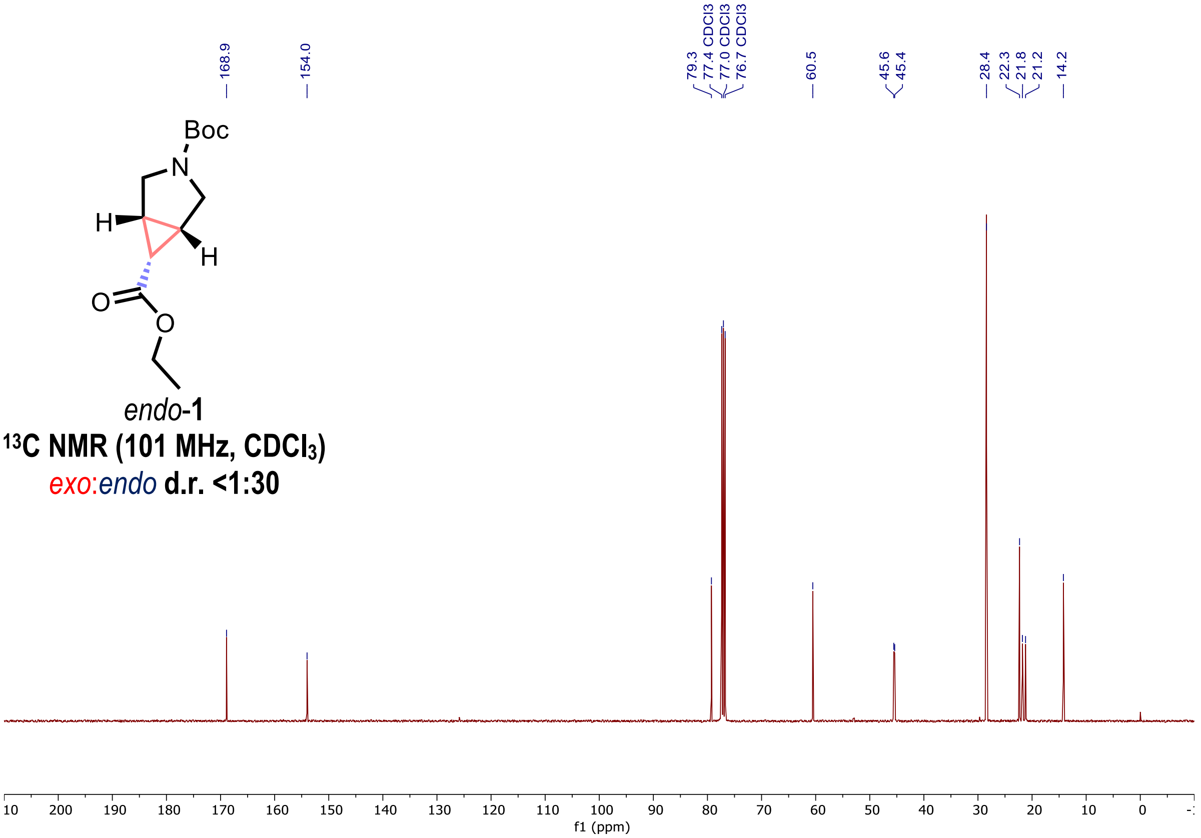
168.9
154.0
79.3 77.4 CDCl3 77.0 CDCl3 76.7 CDCl3
60.5
45.6 45.4
28.4 22.3 21.8 21.2 14.2
Boc
N
H
H
O
O
endo-1
13C NMR (101 MHz, CDCl3)
exo:endo d.r. <1:30
200 190 180 170 160 150 140 130 120 110 100 90 80 70 60 50 40 30 20 10 0
f1 (ppm)

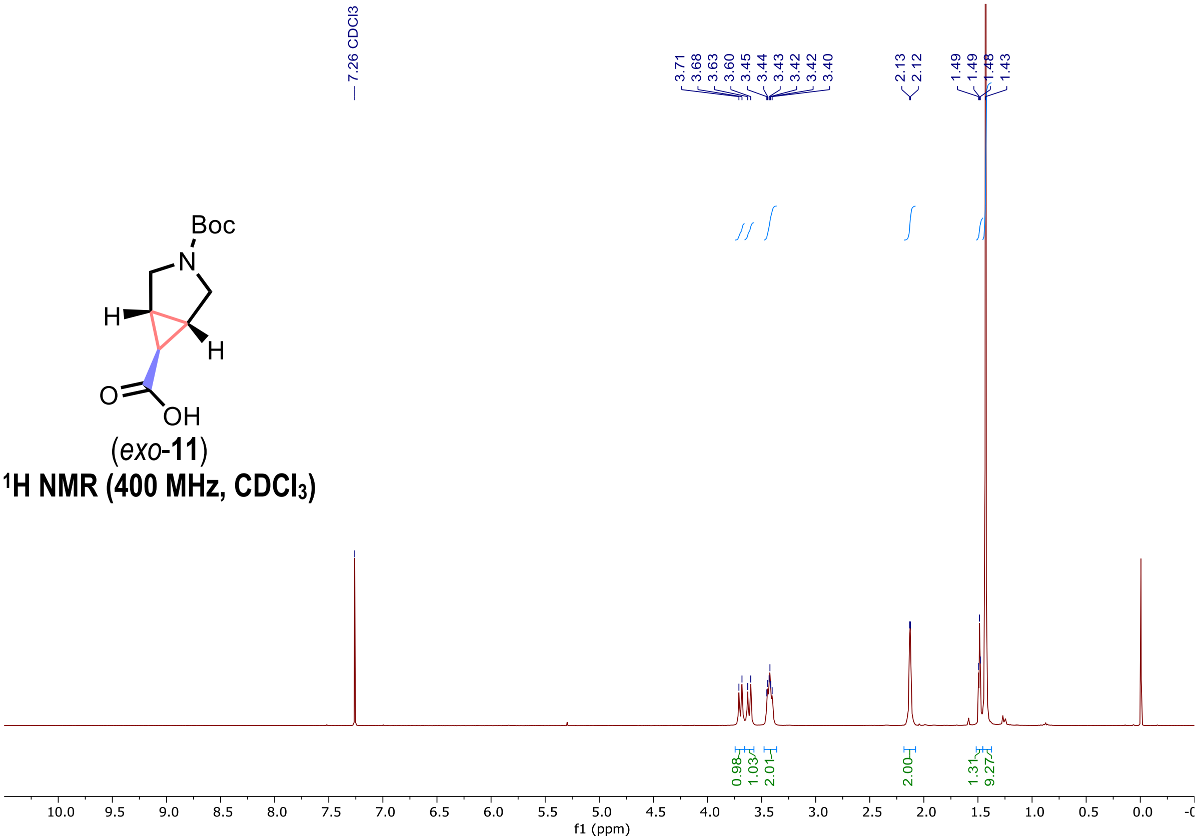

(*exo*-**11**)

^{1}H NMR (400 MHz, CDCl$_3$)

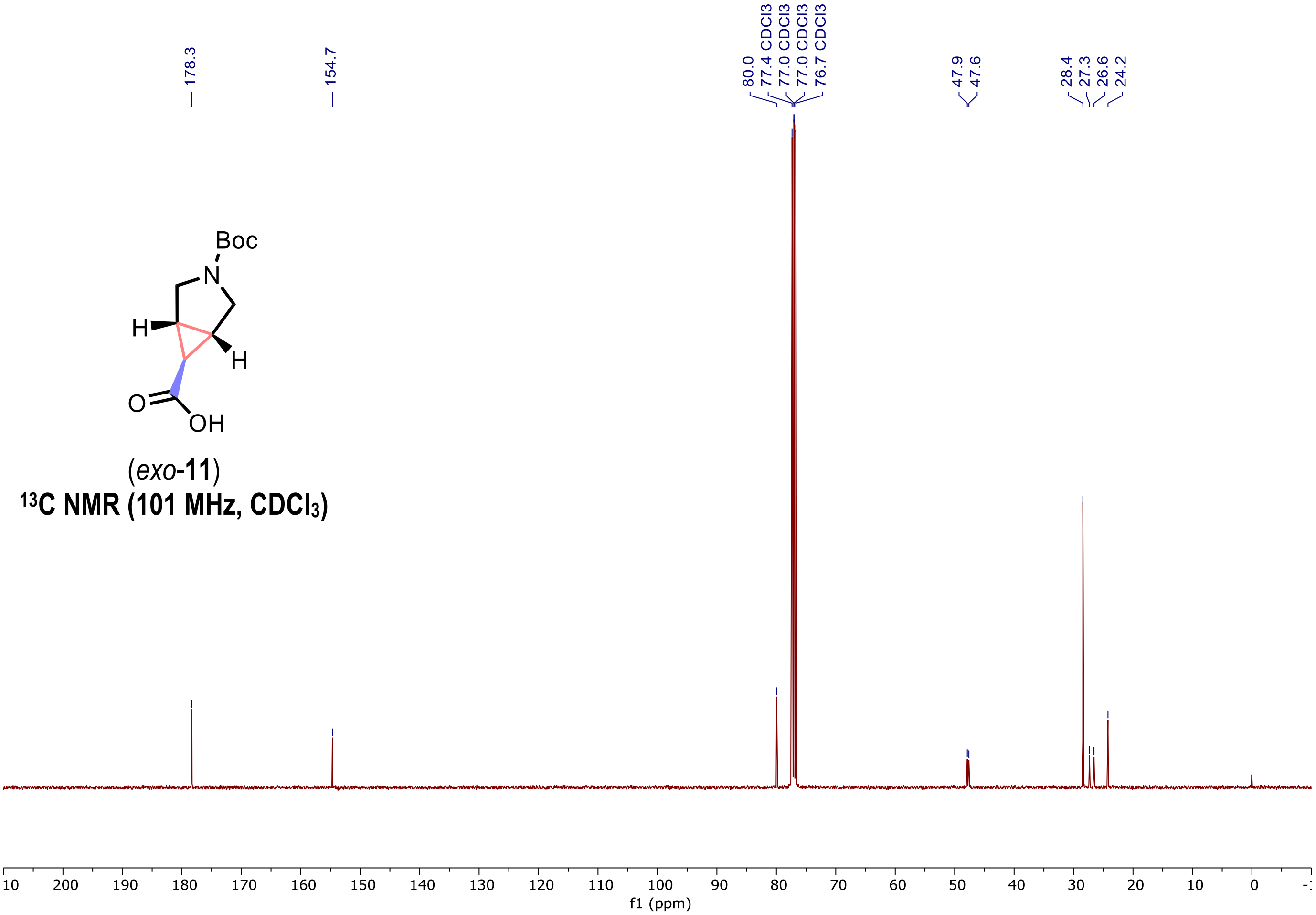

(*exo*-**11**)

^{13}C NMR (101 MHz, CDCl$_3$)

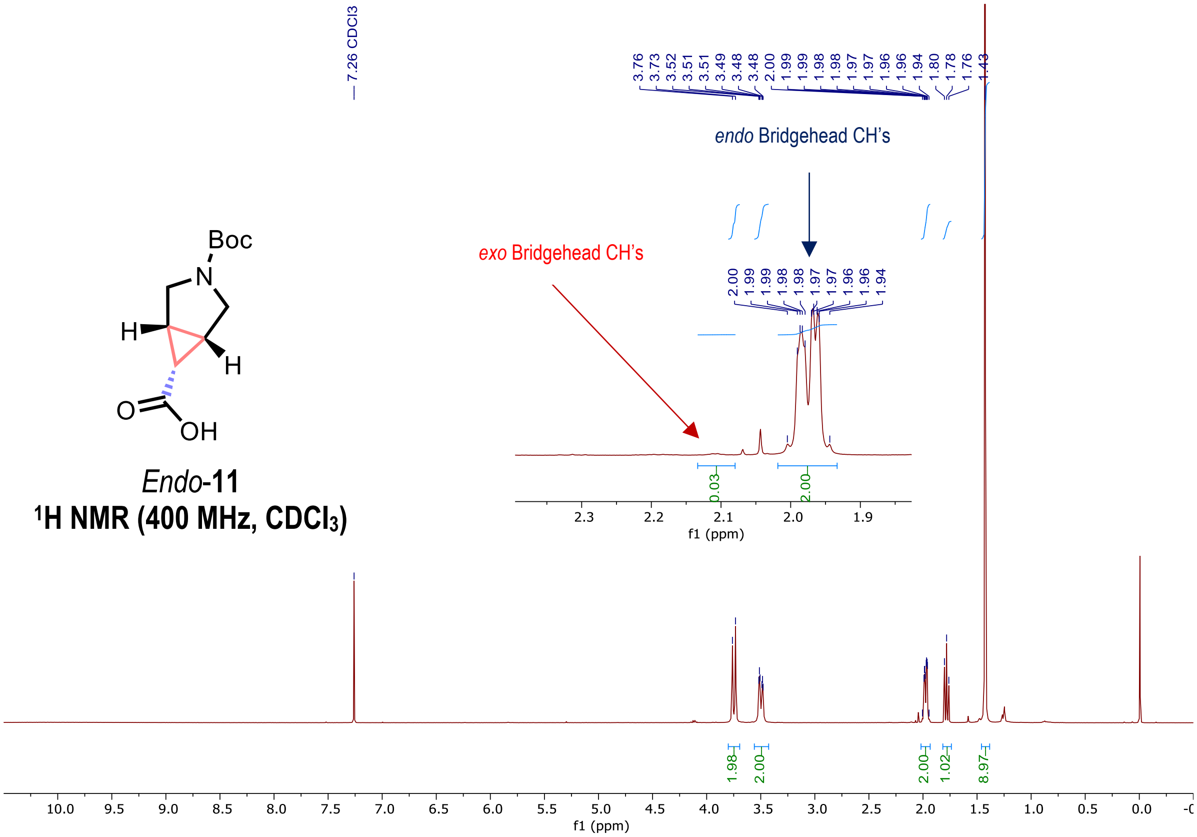

Endo-**11**
^{1}H NMR (400 MHz, $CDCl_3$)

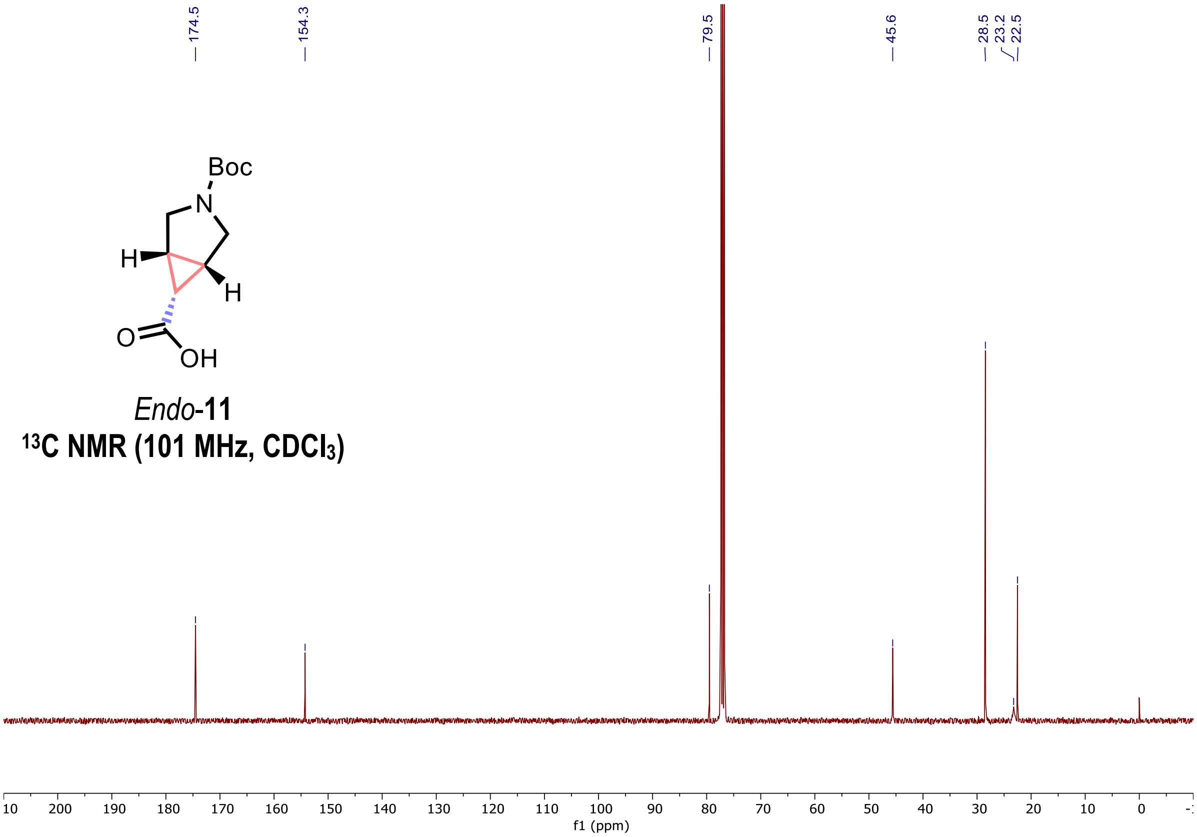

Endo-**11**
^{13}C NMR (101 MHz, $CDCl_3$)

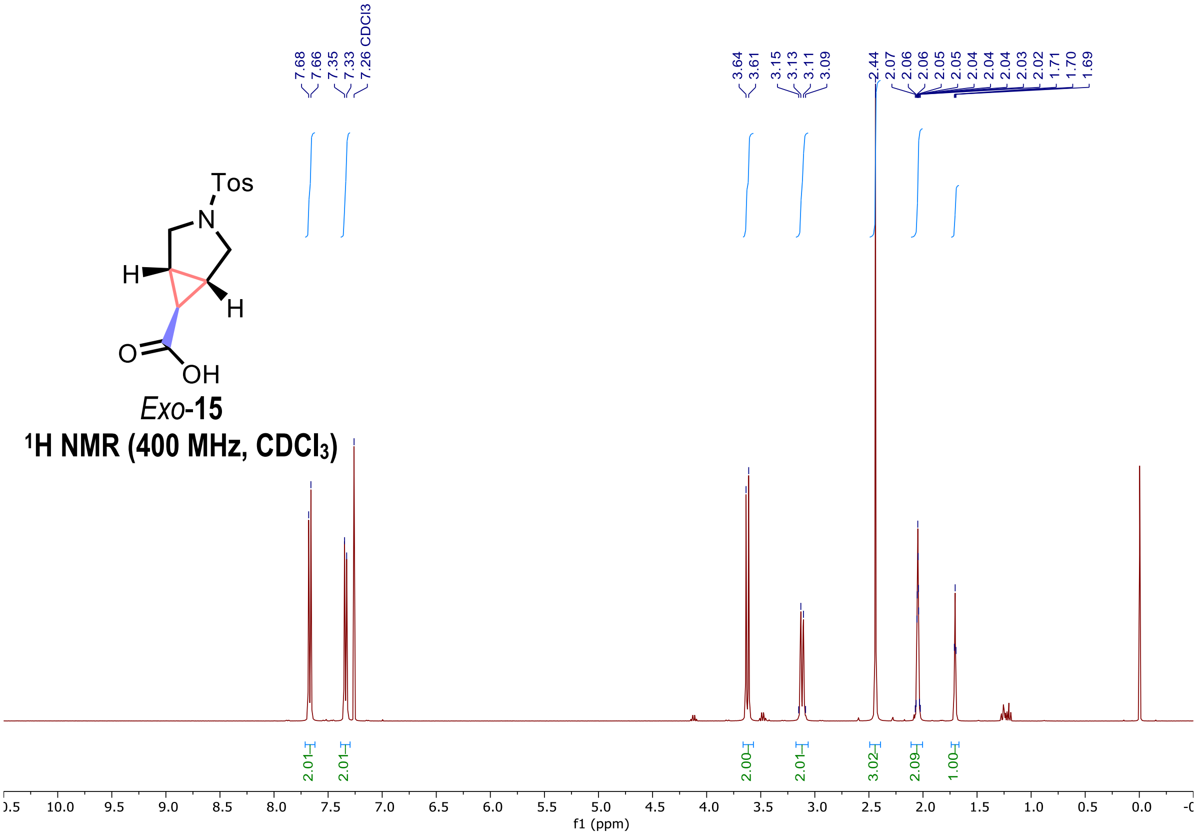

Tos
N
H
H
O
OH
Exo-15
1H NMR (400 MHz, CDCl3)
7.68
7.66
7.35
7.33
7.26 CDCl3
3.64
3.61
3.15
3.13
3.11
3.09
2.44
2.07
2.06
2.06
2.05
2.05
2.04
2.04
2.04
2.03
2.02
1.71
1.70
1.69
2.01
2.01
2.00
2.01
3.02
2.09
1.00
f1 (ppm)

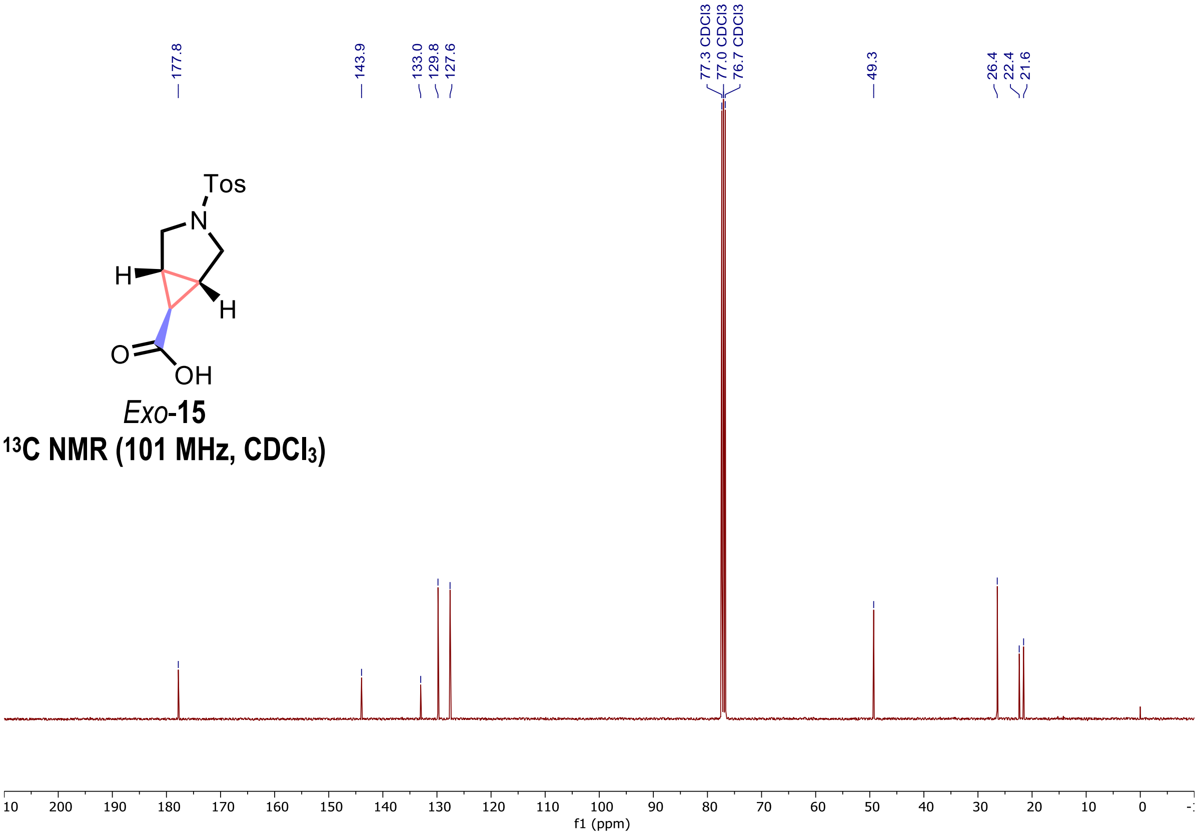

Tos
N
H
H
O
OH
Exo-15
13C NMR (101 MHz, CDCl3)
177.8
143.9
133.0
129.8
127.6
77.3 CDCl3
77.0 CDCl3
76.7 CDCl3
49.3
26.4
22.4
21.6
f1 (ppm)

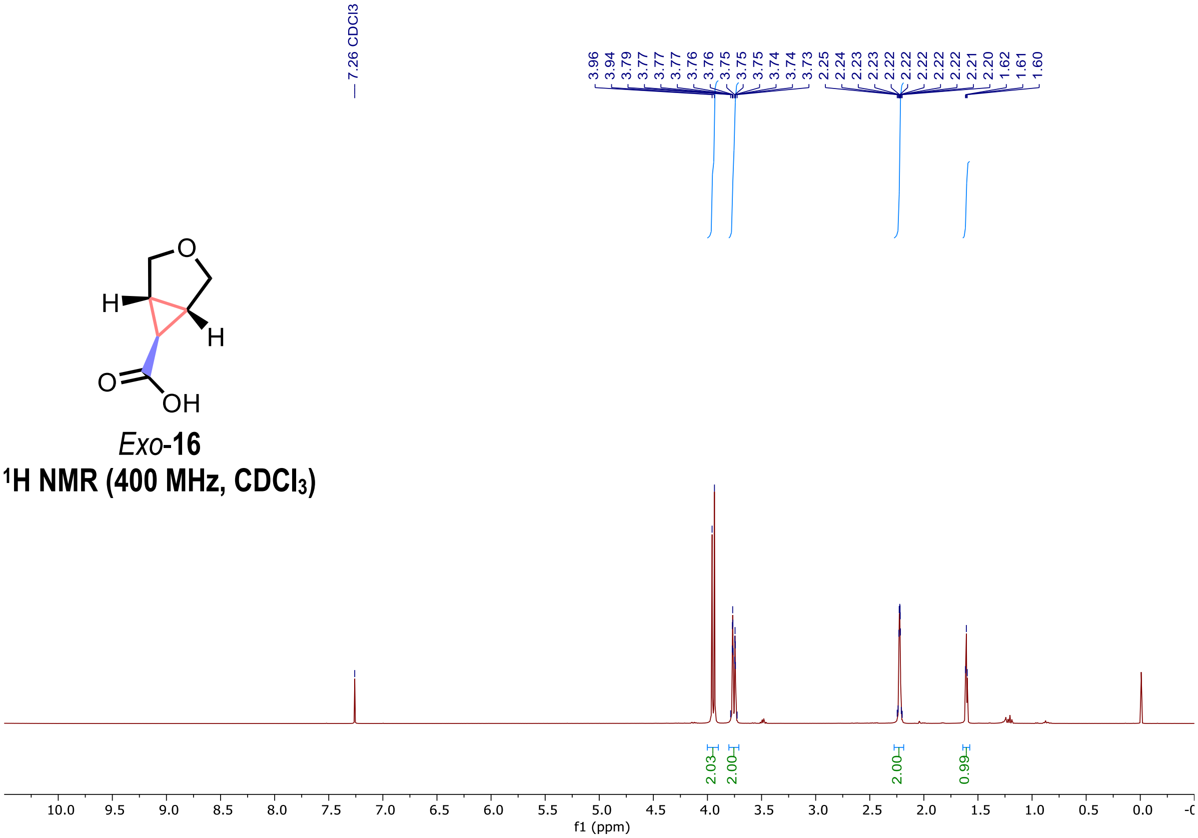

Exo-**16**

^{1}H NMR (400 MHz, CDCl$_3$)

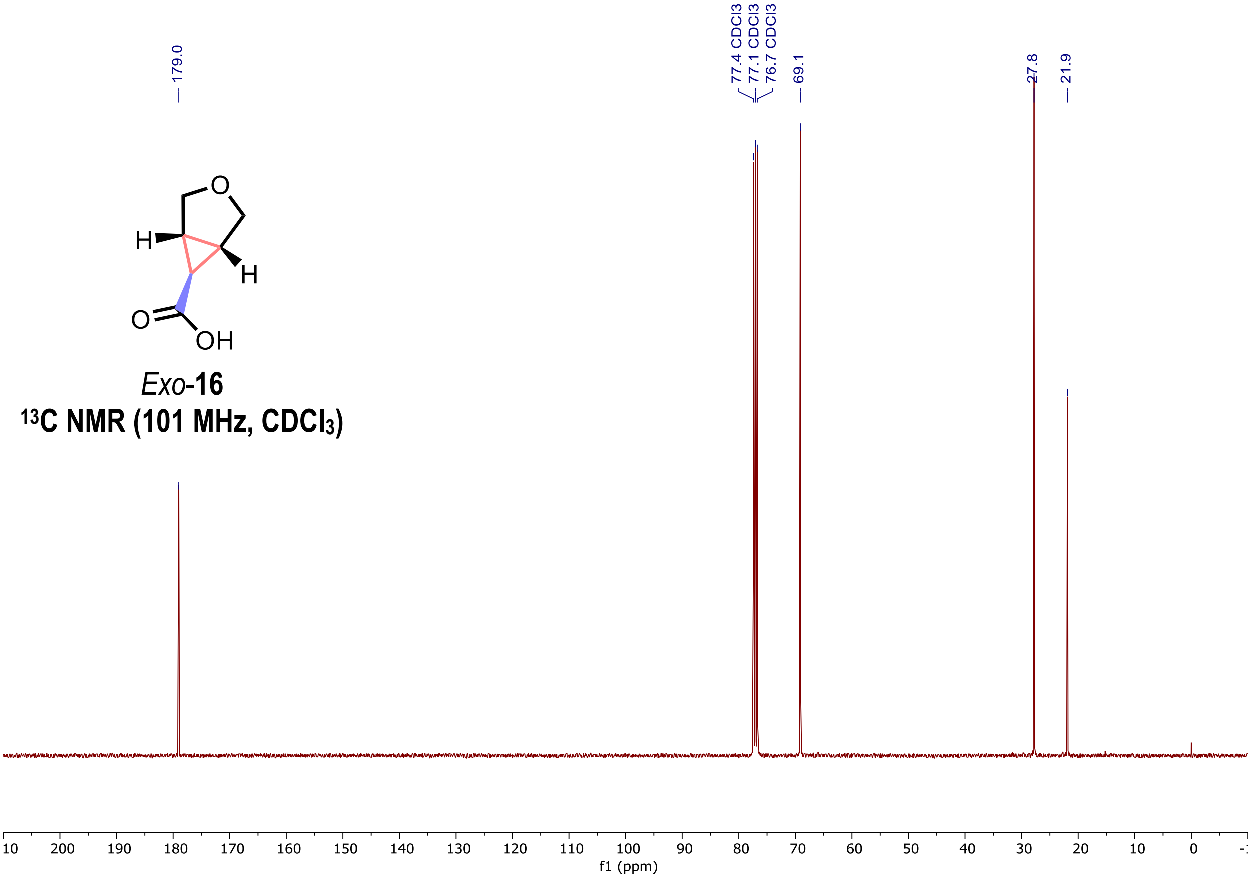

Exo-**16**

^{13}C NMR (101 MHz, CDCl$_3$)

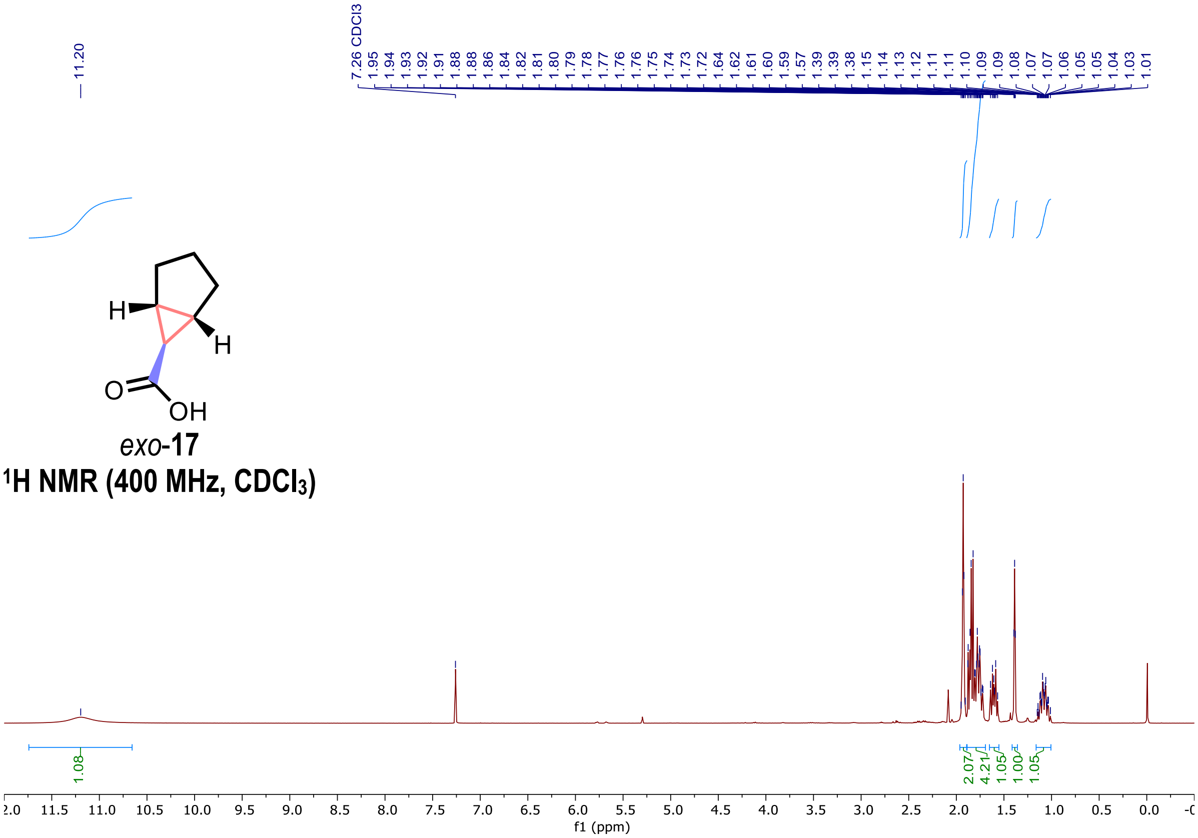
11.20
7.26 CDCl3
1.95 1.94 1.93 1.92 1.91 1.88 1.88 1.86 1.84 1.82 1.81 1.80 1.79 1.78 1.77 1.76 1.76 1.75 1.74 1.73 1.72 1.64 1.62 1.61 1.60 1.59 1.57 1.39 1.39 1.38 1.15 1.14 1.13 1.12 1.11 1.11 1.10 1.09 1.09 1.08 1.07 1.07 1.06 1.05 1.05 1.04 1.03 1.01
H
H
O
OH
exo-17
1H NMR (400 MHz, CDCl3)
1.08
2.07
4.21
1.05
1.00
1.05
f1 (ppm)

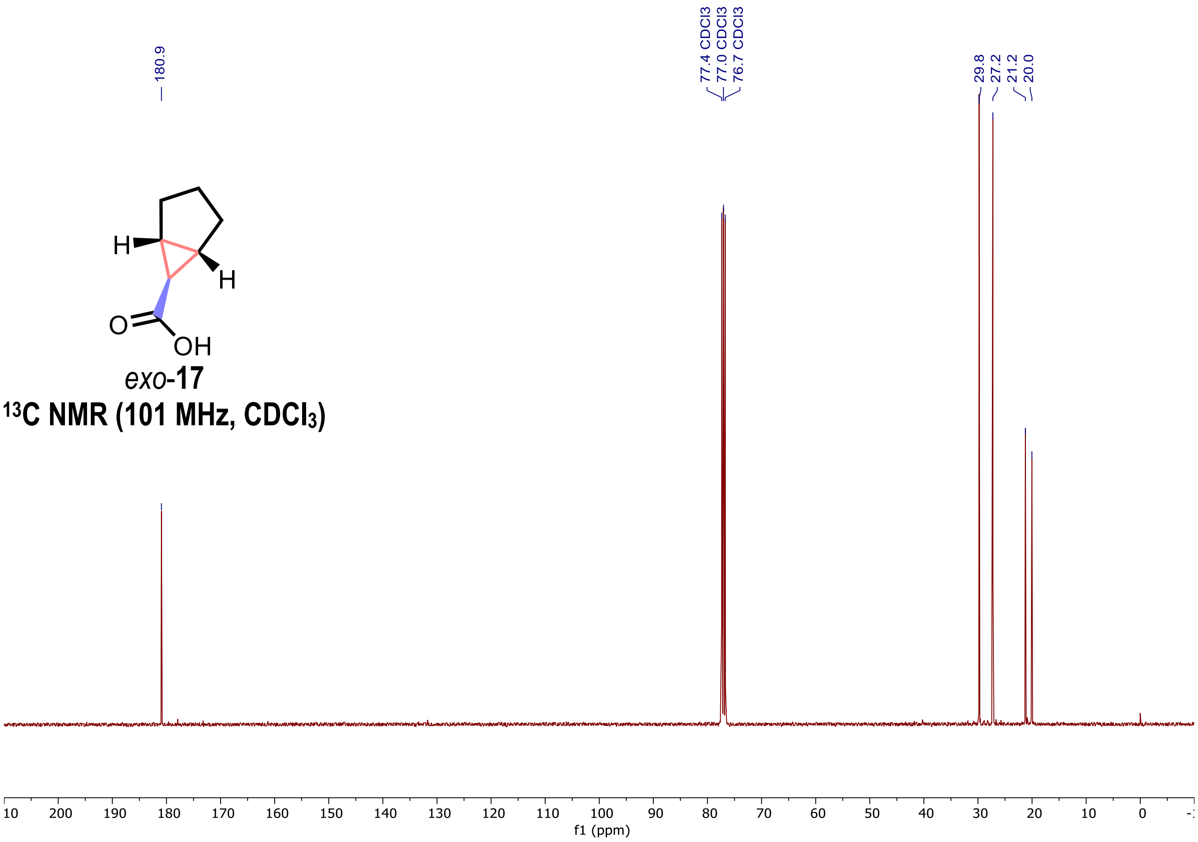
180.9
77.4 CDCl3
77.0 CDCl3
76.7 CDCl3
29.8
27.2
21.2
20.0
H
H
O
OH
exo-17
13C NMR (101 MHz, CDCl3)
f1 (ppm)

12. Crystal Structure Data and Experimental

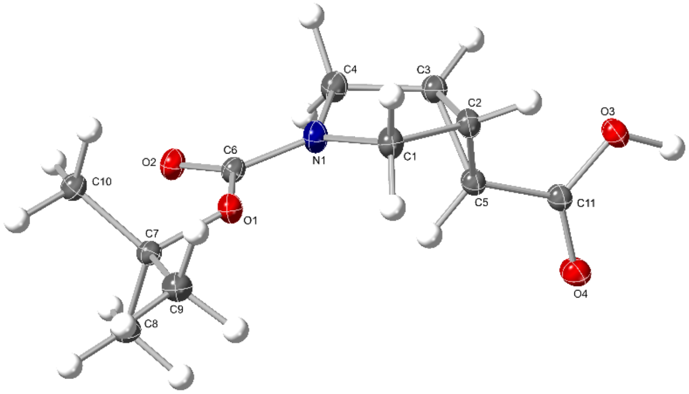

Experimental. Single colorless plate-shaped crystals of (1R,5S,6r)-3-(*tert*-butoxycarbonyl)-3-azabicyclo[3.1.0]hexane-6-carboxylic acid (*exo*-**11**) were chosen from a sample that was recrystallized by slow evaporation in benzene (top layer) and deuterated chloroform (bottom layer) bilayer solvent system over several days. A suitable crystal with dimensions 0.29 × 0.24 × 0.11 mm^3 was selected and mounted on a loop with paratone on a XtaLAB Synergy, Dualflex, HyPix diffractometer. The crystal was kept at a steady T = 100 K during data collection. The structure was solved with the ShelXT 2018/2 (Sheldrick, 2018) solution program using dual methods and by using Olex2 1.5-alpha (Dolomanov et al., 2009) as the graphical interface. The model was refined with olex2.refine 1.5-alpha (Bourhis et al., 2015) using full matrix least squares minimisation on $\boldsymbol{F}^2$.

Crystal Data. $C_{11}H_{17}NO_4$, M_r = 227.262, monoclinic, $P2_1/c$ (No. 14), a = 17.7129(3) Å, b = 5.8559(1) Å, c = 11.0838(2) Å, b = 95.454(2)°, a = g = 90°, V = 1144.46(4) Å^3, T = 100 K, Z = 4, Z' = 1, m(Cu K$_a$) = 0.835, 16241 reflections measured, 2338 unique (R_{int} = 0.0536) which were used in all calculations. The final wR_2 was 0.0942 (all data) and R_1 was 0.0389 (I≥2 s(I)).

**Compound**	(1R,5S,6r)-3-(*tert*-butoxycarbonyl)-3-azabicyclo[3.1.0]hexane-6-carboxylic acid (*exo*-**11**)
Formula	$C_{11}H_{17}NO_4$
$D_{calc.}$/ g cm^{-3}	1.319
m/mm^{-1}	0.835
Formula Weight	227.262
Color	colorless
Shape	plate-shaped
Size/mm^3	0.29×0.24×0.11
T/K	100
Crystal System	monoclinic
Space Group	$P2_1/c$
a/Å	17.7129(3)
b/Å	5.8559(1)
c/Å	11.0838(2)
a/°	90
b/°	95.454(2)
g/°	90
V/Å^3	1144.46(4)
Z	4
Z'	1
Wavelength/Å	1.54184
Radiation type	Cu K$_a$
Q_{min}/°	7.97
Q_{max}/°	77.22
Measured Refl's.	16241
Indep't Refl's	2338
Refl's I≥2 s(I)	2199
R_{int}	0.0536
Parameters	299
Restraints	291
Largest Peak	0.2642
Deepest Hole	-0.2326
GooF	1.0983
wR_2 (all data)	0.0942
wR_2	0.0924
R_1 (all data)	0.0419
R_1	0.0389

Structure Quality Indicators

**Reflections:**	d min (CuKα) 2Θ=154.4° **0.79**	I/σ(I) **34.0**	Rint m=7.21 **5.36%**	Full 135.4° 96% to 154.4° **96.8**
**Refinement:**	Shift **-0.001**	Max Peak **0.3**	Min Peak **-0.2**	GooF **1.098**

A colourless plate-shaped-shaped crystal with dimensions 0.29 × 0.24 × 0.11 mm^3 was mounted on a loop with paratone. Data were collected using a XtaLAB Synergy, Dualflex, HyPix diffractometer equipped with an Oxford Cryosystems low-temperature device operating at T = 100 K.

Data were measured using *w* scans with Cu K_a radiation. The diffraction pattern was indexed and the total number of runs and images was based on the strategy calculation from the program CrysAlisPro 1.171.42.89a (Rigaku OD, 2023). The maximum resolution that was achieved was Q = 77.22° (0.79 Å).

The unit cell was refined using CrysAlisPro 1.171.42.89a (Rigaku OD, 2023) on 9259 reflections, 57% of the observed reflections.

Data reduction, scaling and absorption corrections were performed using CrysAlisPro 1.171.42.89a (Rigaku OD, 2023). The final completeness is 96.80 % out to 77.22° in Q. A numerical absorption correction based on gaussian integration over a multifaceted crystal model was performed using CrysAlisPro 1.171.42.74a (Rigaku Oxford Diffraction, 2022). An empirical absorption correction using spherical harmonics, implemented in SCALE3 ABSPACK scaling algorithm was also applied. The absorption coefficient m of this material is 0.835 mm^{-1} at this wavelength (l = 1.54184 Å) and the minimum and maximum transmissions are 0.618 and 1.000.

The structure was solved and the space group $P2_1/c$ (# 14) determined by the ShelXT 2018/2 (Sheldrick, 2018) structure solution program using using dual methods and refined by full matrix least squares minimisation on $\boldsymbol{F^2}$ using version of olex2.refine 1.5-alpha (Bourhis et al., 2015). All atoms, even hydrogen atoms, were refined anisotropically. Hydrogen atom positions were located from the electron densities and freely refined using Hirshfeld scattering factors. Refinement was by using NoSpherA2, an implementation of non-spherical atom-form-factors (F. Kleemiss, H. Puschmann, O. Dolomanov, S.Grabowsky - https://doi.org/10.1039/D0SC05526C – 2020). NoSpherA2 implementation of HAR makes use of tailor-made aspherical atomic form factors calculated from a Hirshfeld-partitioned electron density (ED) not from spherical-atom form factors. The ED was calculated from a Gaussian basis set single determinant SCF wavefunction from DFT using selected functionals for a fragment of this crystal. This fragment was embedded in an electrostatic crystal field by employing cluster charges. The following options were used: SOFTWARE: X:_B948*T*TN18-067B_02.wfn PARTITIONING: NoSpherA2 INT ACCURACY: Normal METHOD: PBE BASIS SET: def2-TZVP CHARGE: 0 MULTIPLICITY: 1 DATE: 2023-09-06_12-28-06

There is a single formula unit in the asymmetric unit, which is represented by the reported sum formula. In other words: Z is 4 and Z' is 1. The moiety formula is C11 H17 N O4.

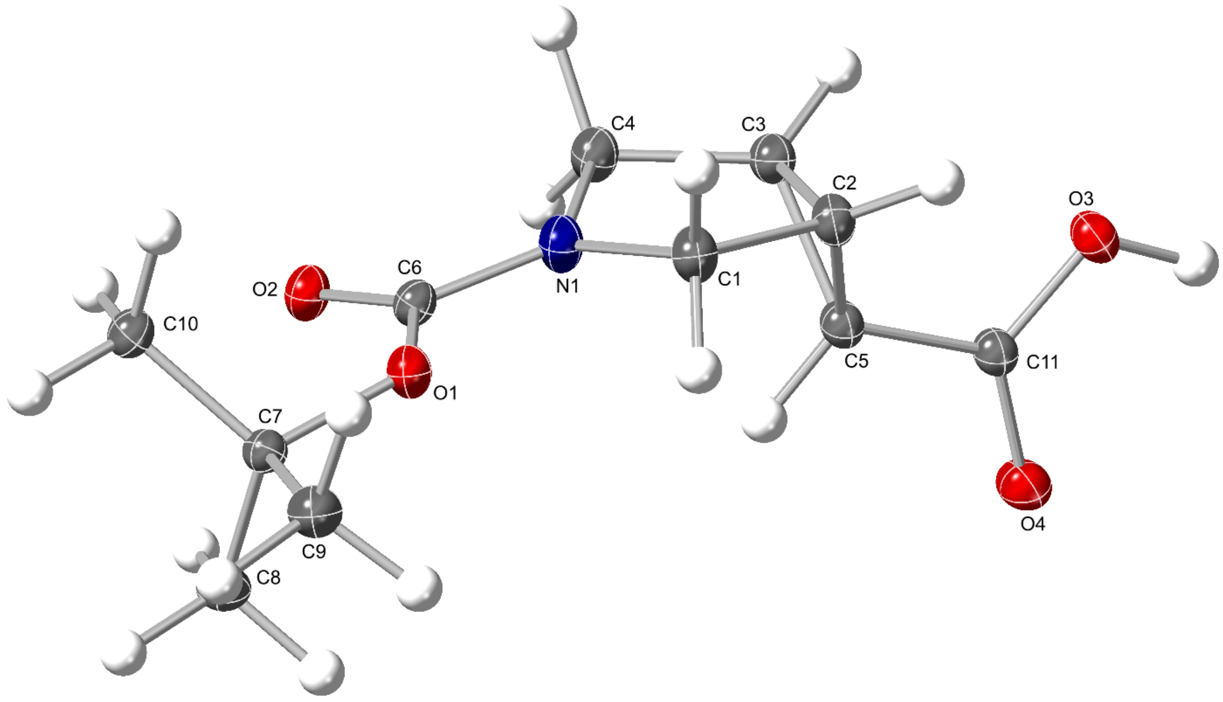

Figure S8. The molecular structure of (1R,5S,6r)-3-(*tert*-butoxycarbonyl)-3-azabicyclo[3.1.0]hexane-6-carboxylic acid (*exo*-**11**) depicted using thermal ellipsoids (50% probability level) for the non-hydrogen atoms.

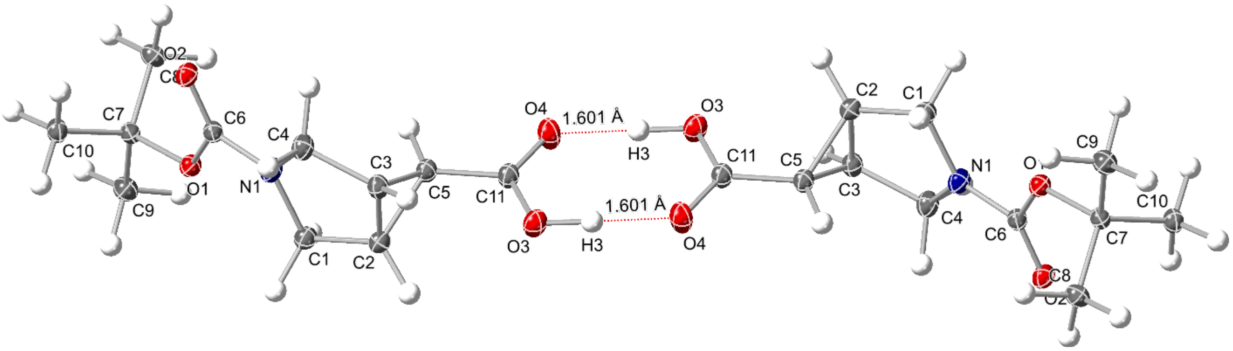

Figure S9. Pairwise hydrogen bonding between the carboxylate groups.

Data Plots: Diffraction Data

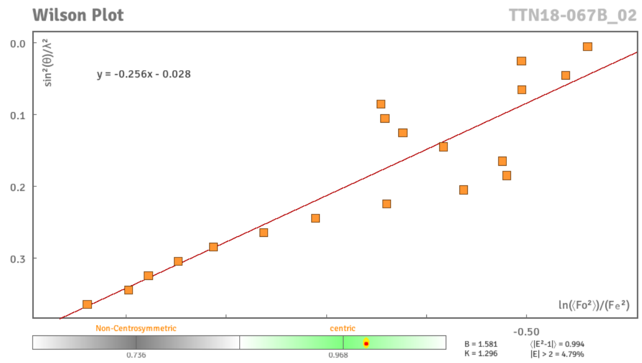

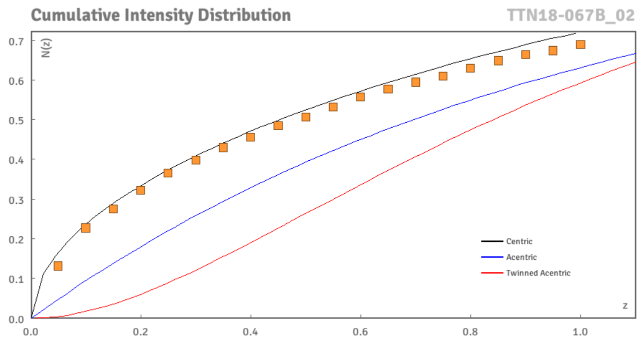

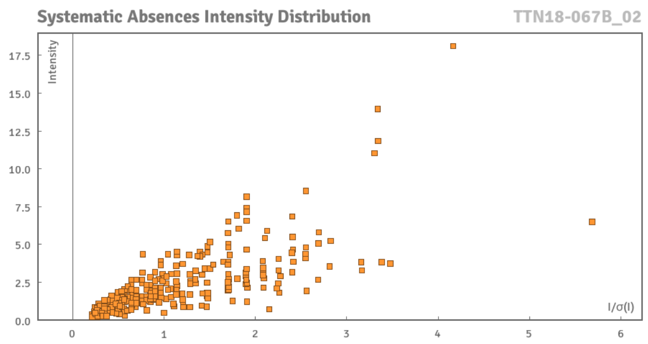

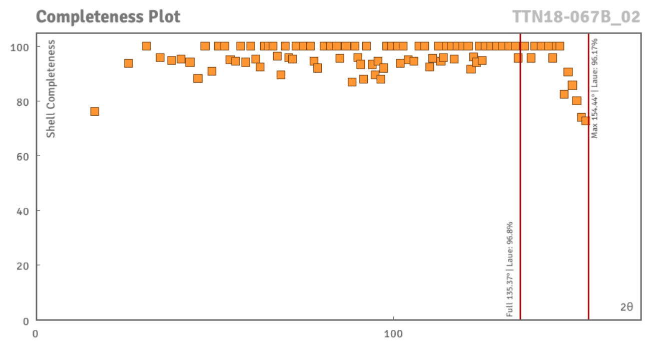

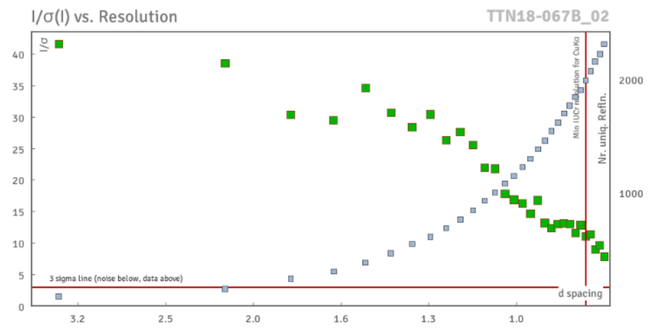

Data Plots: Refinement and Data

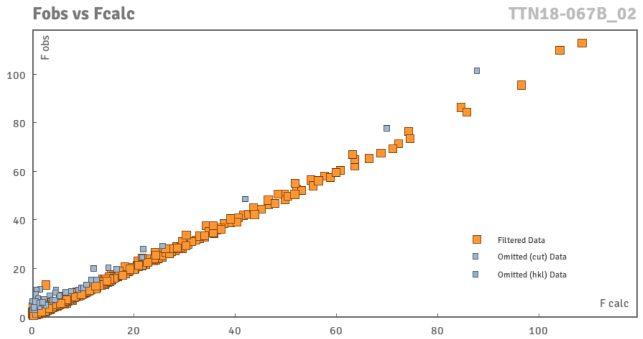

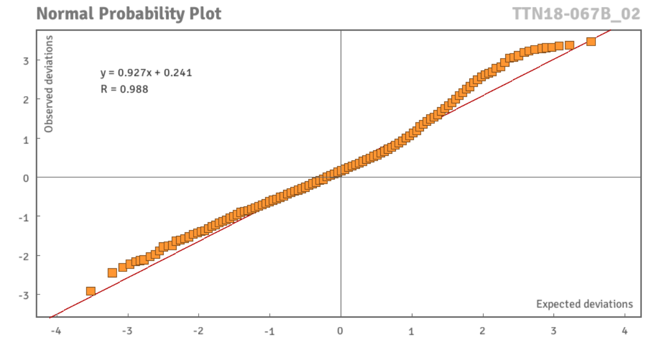

Reflection Statistics

Total reflections (after filtering)	16857	Unique reflections	2338
Completeness	0.962	Mean I/s	20.39
hkl_{max} collected	(22, 7, 13)	hkl_{min} collected	(-22, -7, -14)
hkl_{max} used	(22, 7, 14)	hkl_{min} used	(-22, 0, 0)
Lim d_{max} collected	100.0	Lim d_{min} collected	0.77
d_{max} used	11.03	d_{min} used	0.79
Friedel pairs	2247	Friedel pairs merged	1
Inconsistent equivalents	2	R_{int}	0.0529
R_{sigma}	0.0294	Intensity transformed	0
Omitted reflections	0	Omitted by user (OMIT hkl)	360
Multiplicity	(2268, 1854, 1060, 581, 427, 201, 116, 74, 45, 15, 7)	Maximum multiplicity	22
Removed systematic absences	616	Filtered off (Shel/OMIT)	0

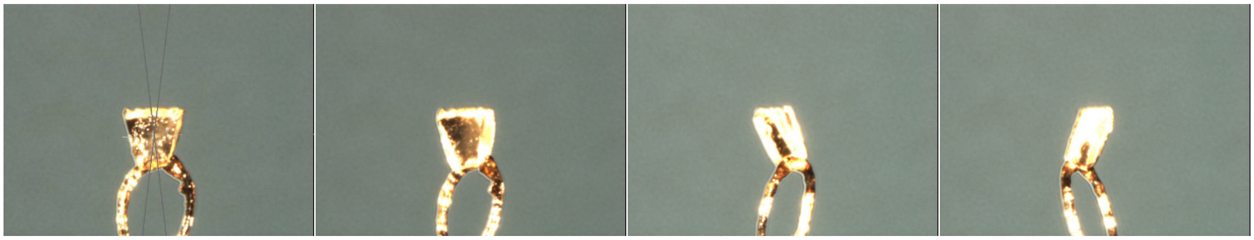

Table S6: Fractional Atomic Coordinates (×10[4]) and Equivalent Isotropic Displacement Parameters (Å[2]×10[3]) for (1R,5S,6r)-3-(*tert*-butoxycarbonyl)-3-azabicyclo[3.1.0]hexane-6-carboxylic acid (*exo*-**11**). U_{eq} is defined as 1/3 of the trace of the orthogonalised U_{ij}.

Atom	x	y	z	U_{eq}
O2	1375.0(5)	2945.0(15)	5469.4(8)	19.2(2)
O1	1939.7(5)	5677.6(15)	4389.9(7)	17.3(2)
O3	4882.5(5)	3393.9(17)	8629.4(9)	24.8(2)
O4	4173.0(5)	6049.7(19)	9427.6(9)	29.1(3)
N1	2651.3(6)	3399.9(18)	5634.9(10)	19.3(2)
C6	1932.1(7)	3938(2)	5179.3(10)	15.2(2)
C7	1224.9(6)	6643(2)	3813.5(11)	15.3(3)
C3	3592.2(7)	1950(2)	7030.6(11)	18.2(3)
C11	4278.4(7)	4627(2)	8616.6(11)	19.9(3)
C2	3920.9(7)	3842(2)	6343.5(11)	18.5(3)
C8	753.3(7)	7627(2)	4768.0(11)	20.0(3)
C10	790.9(7)	4856(2)	3032.3(11)	19.2(3)
C9	1513.2(7)	8543(2)	3035.7(12)	20.1(3)
C4	2798.8(7)	1470(2)	6454.0(12)	20.0(3)
C1	3337.3(7)	4553(2)	5330.3(12)	19.5(3)
C5	3687.1(7)	4309(2)	7607.9(11)	18.5(3)

Table S7: Anisotropic Displacement Parameters (×10[4]) for (1R,5S,6r)-3-(*tert*-butoxycarbonyl)-3-azabicyclo[3.1.0]hexane-6-carboxylic acid (*exo*-**11**). The anisotropic displacement factor exponent takes the form: $-2p^2[h^2a^{*2} \times U_{11} + ... + 2hka^* \times b^* \times U_{12}]$

Atom	U_{11}	U_{22}	U_{33}	U_{23}	U_{13}	U_{12}
O2	17.4(4)	19.7(5)	20.2(4)	-4.3(3)	0.0(3)	3.4(3)
O1	15.8(4)	19.5(4)	16.5(4)	-1.1(3)	0.3(3)	4.2(3)
O3	17.7(4)	28.8(5)	27.0(5)	6.7(4)	-2.3(3)	-5.8(4)
O4	25.7(5)	35.1(6)	25.1(5)	9.7(4)	-4.7(4)	-13.4(4)
N1	16.9(5)	20.6(5)	19.8(5)	-2.5(4)	-1.9(3)	5.8(4)
C6	16.2(5)	15.6(6)	13.6(5)	-1.8(4)	-0.0(4)	0.6(4)
C7	16.4(5)	16.0(6)	13.2(5)	0.8(4)	0.6(4)	-0.5(4)
C3	18.0(6)	19.8(6)	16.4(6)	1.2(4)	-1.4(4)	0.4(4)
C11	16.6(6)	23.2(6)	19.3(6)	3.3(4)	-1.4(4)	-4.7(4)
C2	14.7(6)	22.9(6)	17.9(6)	0.0(4)	0.7(4)	0.0(5)
C8	22.5(6)	20.2(6)	17.5(6)	1.8(5)	4.2(4)	-2.6(5)
C10	19.1(6)	20.9(6)	17.1(6)	-0.7(5)	-0.8(4)	-3.2(5)
C9	23.7(6)	19.4(6)	17.4(6)	0.5(5)	2.2(5)	3.6(5)
C4	20.1(6)	19.7(6)	19.4(6)	-1.5(5)	-2.6(4)	4.2(5)
C1	16.8(6)	23.4(7)	18.2(6)	-1.6(5)	1.0(4)	4.1(5)
C5	15.5(6)	22.0(6)	17.5(6)	2.4(4)	-0.9(4)	-2.2(5)
H2	17(2)	35(9)	38(7)	-0.8(14)	5.0(13)	0(4)
H9a	31(6)	25(5)	20(5)	-5(2)	2(2)	3(2)
H1a	33(7)	36(7)	21(3)	1(3)	6.7(19)	2.8(19)
H4a	33(5)	29(7)	29(5)	-3(3)	8(2)	4(3)
H1b	30(7)	24(2)	38(7)	-0.0(12)	1(3)	4.9(12)
H9b	30(6)	39(7)	25(5)	4(3)	6(2)	-3(3)
H10a	25(4)	28(6)	23(6)	3(2)	-5(2)	-4(3)

Atom	U_{11}	U_{22}	U_{33}	U_{23}	U_{13}	U_{12}
H4b	34(7)	22(3)	26(6)	-1(2)	-3(3)	1.3(18)
H5	22(3)	36(7)	36(7)	10(2)	0(2)	-7(3)
H8a	30(4)	38(7)	31(6)	9(2)	2(2)	2(3)
H10b	31(7)	25(4)	27(6)	-3(2)	3(3)	1(2)
H3a	25(6)	24(4)	33(8)	4(2)	-5(3)	5(2)
H8b	30(7)	28(5)	25(5)	1(2)	7(3)	2(2)
H8c	34(6)	34(5)	35(6)	-5(2)	4(3)	-13(2)
H9c	28(4)	29(6)	26(6)	3(2)	1(2)	8(3)
H10c	31(6)	37(7)	24(5)	3(3)	5(2)	-8(2)
H3	69(10)	160(20)	78(10)	74(9)	-52(5)	-79(8)

Table S8: Bond Lengths in Å for (1R,5S,6r)-3-(*tert*-butoxycarbonyl)-3-azabicyclo[3.1.0]hexane-6-carboxylic acid (*exo*-**11**).

Atom	Atom	Length/Å
O2	C6	1.2146(15)
O1	C6	1.3439(14)
O1	C7	1.4755(13)
O3	C11	1.2898(15)
O3	H3	1.02(2)
O4	C11	1.2526(16)
N1	C6	1.3619(15)
N1	C4	1.4578(16)
N1	C1	1.4575(16)
C7	C8	1.5221(17)
C7	C10	1.5195(17)
C7	C9	1.5249(17)
C3	C2	1.4935(17)
C3	C4	1.5140(16)
C3	C5	1.5250(18)
C3	H3a	1.089(16)
C11	C5	1.4693(16)
C2	C1	1.5109(16)
C2	C5	1.5234(17)
C2	H2	1.054(15)
C8	H8a	1.091(16)
C8	H8b	1.091(17)
C8	H8c	1.100(17)
C10	H10a	1.084(15)
C10	H10b	1.075(16)
C10	H10c	1.079(16)
C9	H9a	1.102(16)
C9	H9b	1.092(16)
C9	H9c	1.112(16)
C4	H4a	1.081(16)
C4	H4b	1.129(17)
C1	H1a	1.094(16)
C1	H1b	1.082(17)
C5	H5	1.047(15)

Table S9: Bond Angles in ˚ for (1R,5S,6r)-3-(*tert*-butoxycarbonyl)-3-azabicyclo[3.1.0]hexane-6-carboxylic acid (*exo*-**11**).

Atom	Atom	Atom	Angle/˚
C7	O1	C6	120.74(9)
H3	O3	C11	113.2(15)
C4	N1	C6	121.07(10)
C1	N1	C6	125.31(10)
C1	N1	C4	113.56(10)
O1	C6	O2	126.44(11)
N1	C6	O2	123.09(11)
N1	C6	O1	110.47(10)
C8	C7	O1	110.44(9)
C10	C7	O1	110.47(10)
C10	C7	C8	112.04(10)
C9	C7	O1	101.81(9)
C9	C7	C8	110.63(10)
C9	C7	C10	111.01(10)
C4	C3	C2	108.30(10)
C5	C3	C2	60.61(8)
C5	C3	C4	113.98(10)
H3a	C3	C2	120.3(9)
H3a	C3	C4	121.5(9)
H3a	C3	C5	117.2(9)
O4	C11	O3	122.99(11)
C5	C11	O3	117.91(11)
C5	C11	O4	119.09(11)
C1	C2	C3	108.13(10)
C5	C2	C3	60.72(8)
C5	C2	C1	114.05(10)
H2	C2	C3	122.3(9)
H2	C2	C1	120.5(9)
H2	C2	C5	116.9(9)
H8a	C8	C7	109.7(9)
H8b	C8	C7	112.8(9)
H8b	C8	H8a	109.2(12)
H8c	C8	C7	111.3(9)
H8c	C8	H8a	106.8(14)
H8c	C8	H8b	106.6(13)
H10a	C10	C7	109.1(8)

Atom	Atom	Atom	Angle/°
H10b	C10	C7	111.6(9)
H10b	C10	H10a	106.7(12)
H10c	C10	C7	114.1(9)
H10c	C10	H10a	108.8(12)
H10c	C10	H10b	106.4(13)
H9a	C9	C7	110.0(8)
H9b	C9	C7	110.6(10)
H9b	C9	H9a	107.1(12)
H9c	C9	C7	111.0(8)
H9c	C9	H9a	108.1(13)
H9c	C9	H9b	109.8(12)
C3	C4	N1	102.88(10)
H4a	C4	N1	112.6(9)
H4a	C4	C3	111.5(9)
H4b	C4	N1	112.8(8)

Atom	Atom	Atom	Angle/°
H4b	C4	C3	109.3(8)
H4b	C4	H4a	107.7(12)
C2	C1	N1	103.10(10)
H1a	C1	N1	116.4(9)
H1a	C1	C2	108.6(9)
H1b	C1	N1	110.2(9)
H1b	C1	C2	113.5(9)
H1b	C1	H1a	105.3(13)
C11	C5	C3	118.43(11)
C2	C5	C3	58.67(8)
C2	C5	C11	119.09(10)
H5	C5	C3	117.5(9)
H5	C5	C11	114.2(9)
H5	C5	C2	118.1(9)

Table S10: Torsion Angles in ° for (1R,5S,6r)-3-(*tert*-butoxycarbonyl)-3-azabicyclo[3.1.0]hexane-6-carboxylic acid (*exo*-**11**).

Atom	Atom	Atom	Atom	Angle/°
O2	C6	O1	C7	2.66(16)
O2	C6	N1	C4	4.03(15)
O2	C6	N1	C1	-179.15(12)
O1	C6	N1	C4	-176.06(9)
O1	C6	N1	C1	0.77(13)
O3	C11	C5	C3	30.85(14)
O3	C11	C5	C2	-37.10(14)
O4	C11	C5	C3	-148.36(12)
O4	C11	C5	C2	143.69(12)
N1	C4	C3	C2	-11.42(11)
N1	C4	C3	C5	53.83(11)
N1	C1	C2	C3	11.72(11)
N1	C1	C2	C5	-53.60(11)
C3	C2	C5	C11	107.41(9)
C3	C5	C2	C1	98.06(9)
C11	C5	C2	C1	-154.53(12)

Table S11: Hydrogen Fractional Atomic Coordinates (×10⁴) and Equivalent Isotropic Displacement Parameters (Å^2×10^3) for (1R,5S,6r)-3-(*tert*-butoxycarbonyl)-3-azabicyclo[3.1.0]hexane-6-carboxylic acid (*exo*-**11**). U_{eq} is defined as 1/3 of the trace of the orthogonalised U_{ij}.

Atom	x	y	z	U_{eq}
H2	4504(9)	3940(30)	6233(15)	30(3)
H9a	1869(9)	9740(30)	3608(14)	26(3)
H1a	3546(9)	4090(30)	4466(15)	30(3)
H4a	2396(9)	1360(30)	7126(15)	30(3)
H1b	3245(9)	6380(30)	5280(16)	31(3)
H9b	1867(9)	7840(30)	2370(15)	31(3)
H10a	295(9)	5650(30)	2557(14)	26(3)
H4b	2794(9)	-220(30)	5964(15)	28(3)
H5	3195(9)	5270(30)	7683(15)	31(4)
H8a	253(9)	8480(30)	4327(16)	33(3)
H10b	589(9)	3500(30)	3573(15)	28(3)
H3a	3955(9)	590(30)	7426(15)	28(3)
H8b	575(9)	6330(30)	5390(15)	27(3)

Atom	x	y	z	U_{eq}
H8c	1073(10)	8920(30)	5326(16)	34(3)
H9c	1034(9)	9520(30)	2566(15)	27(3)
H10c	1118(9)	4050(30)	2378(15)	30(3)
H3	5251(16)	3640(60)	9380(30)	105(12)

Table S12: Hydrogen Bond information for (1R,5S,6r)-3-(*tert*-butoxycarbonyl)-3-azabicyclo[3.1.0]hexane-6-carboxylic acid (*exo*-**11**).

D	H	A	d(D-H)/Å	d(H-A)/Å	d(D-A)/Å	D-H-A/deg
O3	H3	O4[1]	1.02(2)	1.60(3)	2.6188(13)	178(4)

[1]1-x,1-y,2-

Citations

CrysAlisPro Software System, Rigaku Oxford Diffraction, (2023).

L.J. Bourhis and O.V. Dolomanov and R.J. Gildea and J.A.K. Howard and H. Puschmann, The Anatomy of a Comprehensive Constrained, Restrained, Refinement Program for the Modern Computing Environment - Olex2 Disected, *Acta Cryst. A*, (2015), **A71**, 59-71.

O.V. Dolomanov and L.J. Bourhis and R.J. Gildea and J.A.K. Howard and H. Puschmann, Olex2: A complete structure solution, refinement and analysis program, *J. Appl. Cryst.*, (2009), **42**, 339-341.

Sheldrick, G.M., ShelXT-Integrated space-group and crystal-structure determination, *Acta Cryst.*, (2015), **A71**, 3-8.

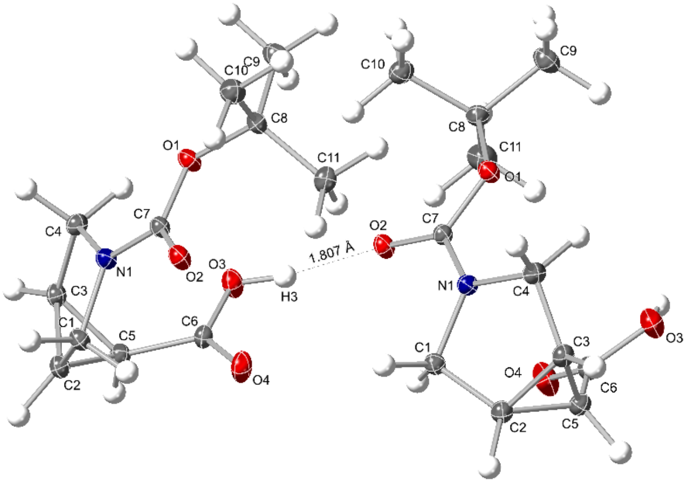

Experimental. Single colorless prism-shaped crystals of (1R,5S,6s)-3-(*tert*-butoxycarbonyl)-3-azabicyclo[3.1.0]hexane-6-carboxylic acid (*endo*-**11**) were chosen from a sample that was recrystallized by slow evaporation in benzene (top layer) and deuterated chloroform (bottom layer) bilayer solvent system over several days. A suitable crystal with dimensions 0.62 × 0.53 × 0.40 mm³ was selected and mounted on a loop with paratone on a XtaLAB Synergy, Dualflex, HyPix diffractometer. The crystal was kept at a steady T = 100 K during data collection. The structure was solved with the ShelXT 2018/2 (Sheldrick, 2018) solution program using dual methods and by using Olex2 1.5-alpha (Dolomanov et al., 2009) as the graphical interface. The model was refined with olex2.refine 1.5-alpha (Bourhis et al., 2015) using full matrix least squares minimisation on F^2.

Crystal Data. $C_{11}H_{17}NO_4$, M_r = 227.262, monoclinic, $P2_1/c$ (No. 14), a = 9.1076(1) Å, b = 11.8282(2) Å, c = 11.4349(2) Å, b = 112.240(2)°, a = g = 90°, V = 1140.20(4) Å³, T = 100 K, Z = 4, Z' = 1, m(Cu K_a) = 0.838, 28984 reflections measured, 2323 unique (R_{int} = 0.0699) which were used in all calculations. The final wR_2 was 0.0537 (all data) and R_1 was 0.0243 (I≥2 s(I)).

**Compound**	(1R,5S,6s)-3-(*tert*-butoxycarbonyl)-3-azabicyclo[3.1.0]hexane-6-carboxylic acid (*endo*-**11**)
Formula	$C_{11}H_{17}NO_4$
$D_{calc.}$/ g cm^{-3}	1.324
m/mm^{-1}	0.838
Formula Weight	227.262
Color	colorless
Shape	prism-shaped
Size/mm³	0.62×0.53×0.40
T/K	100
Crystal System	monoclinic
Space Group	$P2_1/c$
a/Å	9.1076(1)
b/Å	11.8282(2)
c/Å	11.4349(2)
a/°	90
b/°	112.240(2)
g/°	90
V/Å³	1140.20(4)
Z	4
Z'	1
Wavelength/Å	1.54184
Radiation type	Cu K_a
Q_{min}/°	5.25
Q_{max}/°	76.98
Measured Refl's.	28984
Indep't Refl's	2323
Refl's I≥2 s(I)	2083
R_{int}	0.0699
Parameters	299
Restraints	189
Largest Peak	0.2267
Deepest Hole	-0.2528
GooF	1.1913
wR_2 (all data)	0.0537
wR_2	0.0456
R_1 (all data)	0.0358
R_1	0.0243

Structure Quality Indicators

**Reflections:**	d min (CuKα) 2Θ=154.0° 0.79	I/σ(I) 34.7	Rint m=12.96 6.99%	Full 135.4° 96% to 154.0° 98.4
**Refinement:**	Shift -0.001	Max Peak 0.2	Min Peak -0.3	GooF 1.191

A colourless prism-shaped-shaped crystal with dimensions 0.62 × 0.53 × 0.40 mm^3 was mounted on a loop with paratone. Data were collected using a XtaLAB Synergy, Dualflex, HyPix diffractometer equipped with an Oxford Cryosystems low-temperature device operating at *T* = 100 K.

Data were measured using *w* scans with Cu K_a radiation. The diffraction pattern was indexed and the total number of runs and images was based on the strategy calculation from the program CrysAlisPro 1.171.42.89a (Rigaku OD, 2023). The maximum resolution that was achieved was *Q* = 76.98° (0.79 Å).

The unit cell was refined using CrysAlisPro 1.171.42.89a (Rigaku OD, 2023) on 13920 reflections, 48% of the observed reflections.

Data reduction, scaling and absorption corrections were performed using CrysAlisPro 1.171.42.89a (Rigaku OD, 2023). The final completeness is 98.44 % out to 76.98° in Q. A numerical absorption correction based on gaussian integration over a multifaceted crystal model was performed using CrysAlisPro 1.171.42.74a (Rigaku Oxford Diffraction, 2022). An empirical absorption correction using spherical harmonics, implemented in SCALE3 ABSPACK scaling algorithm was also applied. The absorption coefficient *m* of this material is 0.838 mm^{-1} at this wavelength (*l* = 1.54184 Å) and the minimum and maximum transmissions are 0.324 and 1.000.

The structure was solved and the space group $P2_1/c$ (# 14) determined by the ShelXT 2018/2 (Sheldrick, 2018) structure solution program using using dual methods and refined by full matrix least squares minimisation on $\boldsymbol{F^2}$ using version of olex2.refine 1.5-alpha (Bourhis et al., 2015). All atoms, even hydrogen atoms, were refined anisotropically. Hydrogen atom positions were located from the electron densities and freely refined using Hirshfeld scattering factors. Refinement was by using NoSpherA2, an implementation of non-spherical atom-form-factors (F. Kleemiss, H. Puschmann, O. Dolomanov, S.Grabowsky - https://doi.org/10.1039/D0SC05526C – 2020). NoSpherA2 implementation of HAR makes use of tailor-made aspherical atomic form factors calculated from a Hirshfeld-partitioned electron density (ED) not from spherical-atom form factors. The ED was calculated from a Gaussian basis set single determinant SCF wavefunction from DFT using selected functionals for a fragment of this crystal. This fragment was embedded in an electrostatic crystal field by employing cluster charges. The following options were used: SOFTWARE: ORCA PARTITIONING: NoSpherA2 INT ACCURACY: Normal METHOD: PBE BASIS SET: def2-TZVP CHARGE: 0 MULTIPLICITY: 1 SOLVATION: CH2CL2 DATE: 2023-09-28_13-02-29

There is a single formula unit in the asymmetric unit, which is represented by the reported sum formula. In other words: Z is 4 and Z' is 1. The moiety formula is C11 H17 N O4.

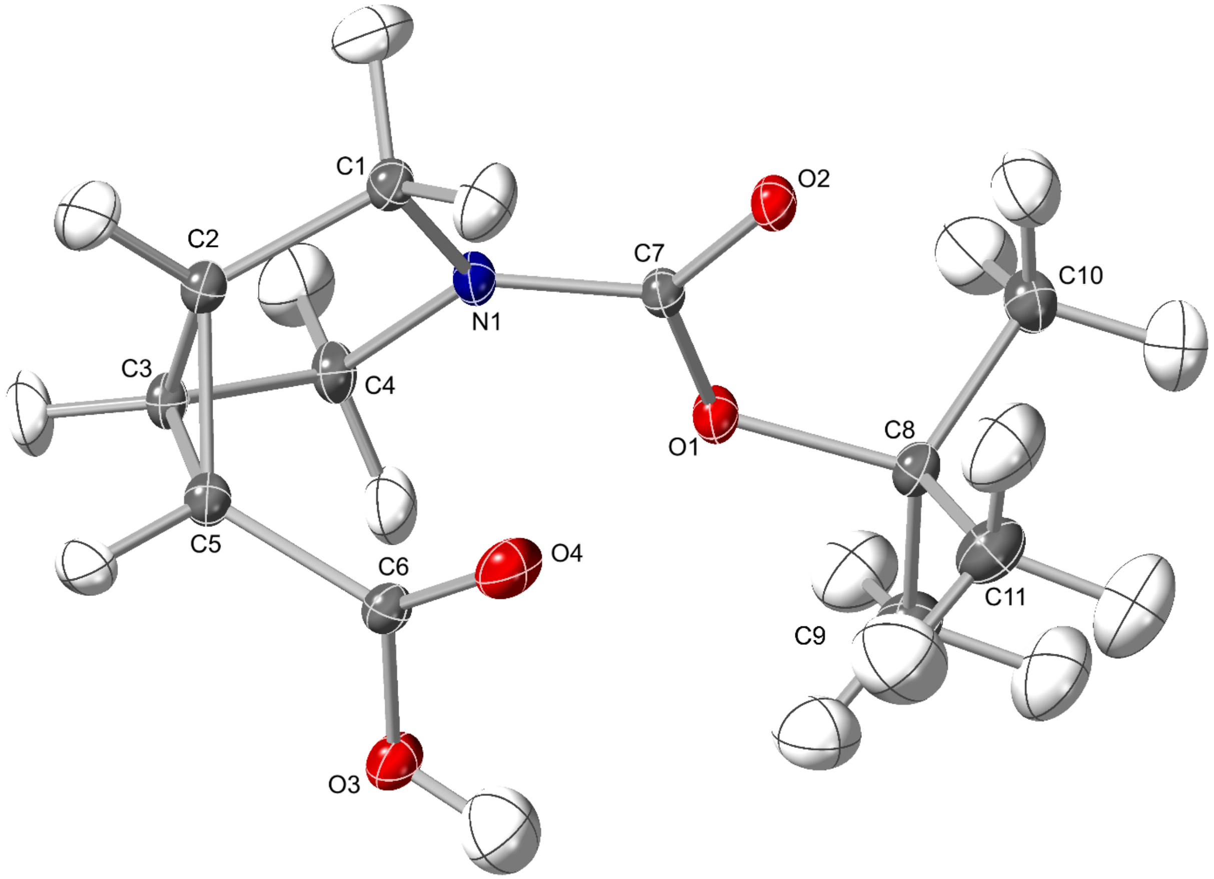

Figure S10. (1R,5S,6s)-3-(*tert*-butoxycarbonyl)-3-azabicyclo[3.1.0]hexane-6-carboxylic acid (*endo*-**11**) depicted using thermal ellipsoids (50% probability level for all atoms). The non-linear geometry of the molecule, with its t-butyl group curved inwards toward the carboxylate group is interesting (a non-planar (*tert*-butoxycarbonyl) group).

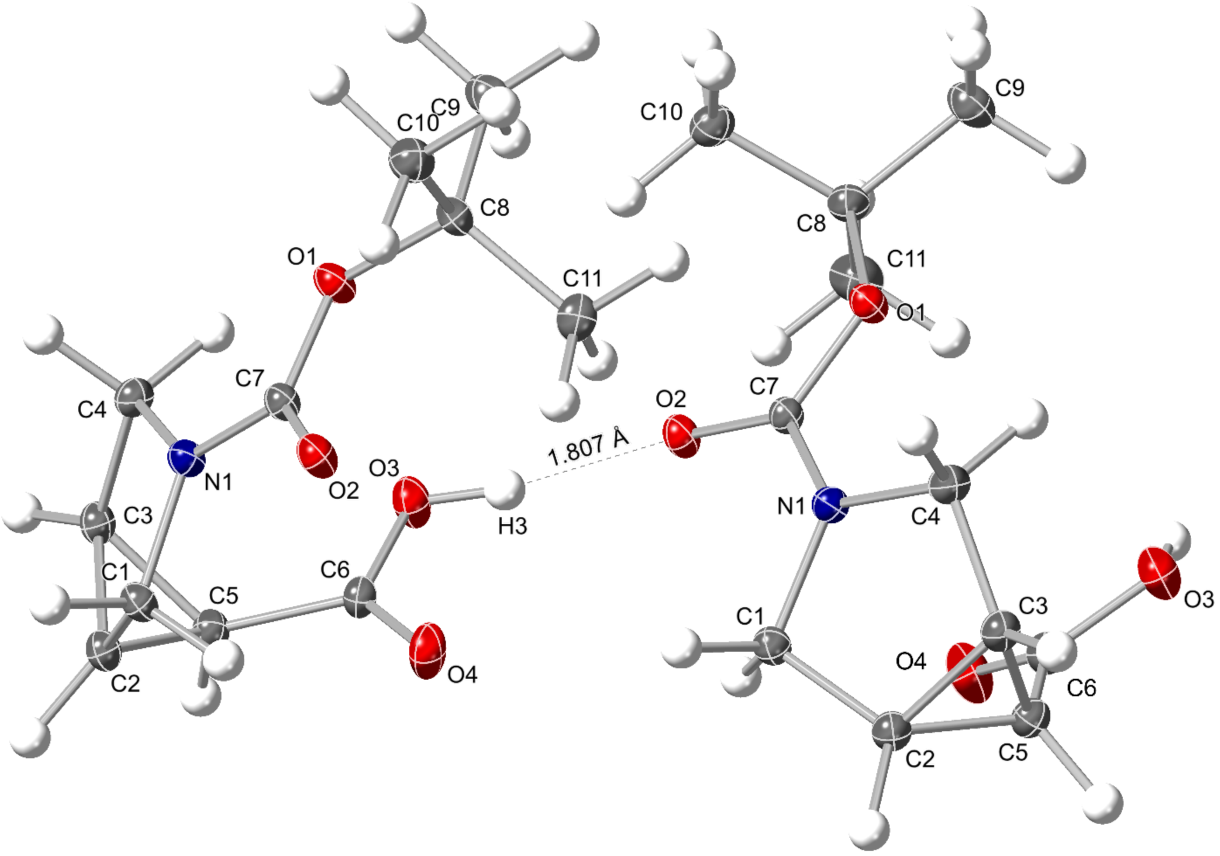

Figure S11. The carboxylate group donates a hydrogen bond to the atom O2 (linking the molecules to form an infinite chain in the crystal).

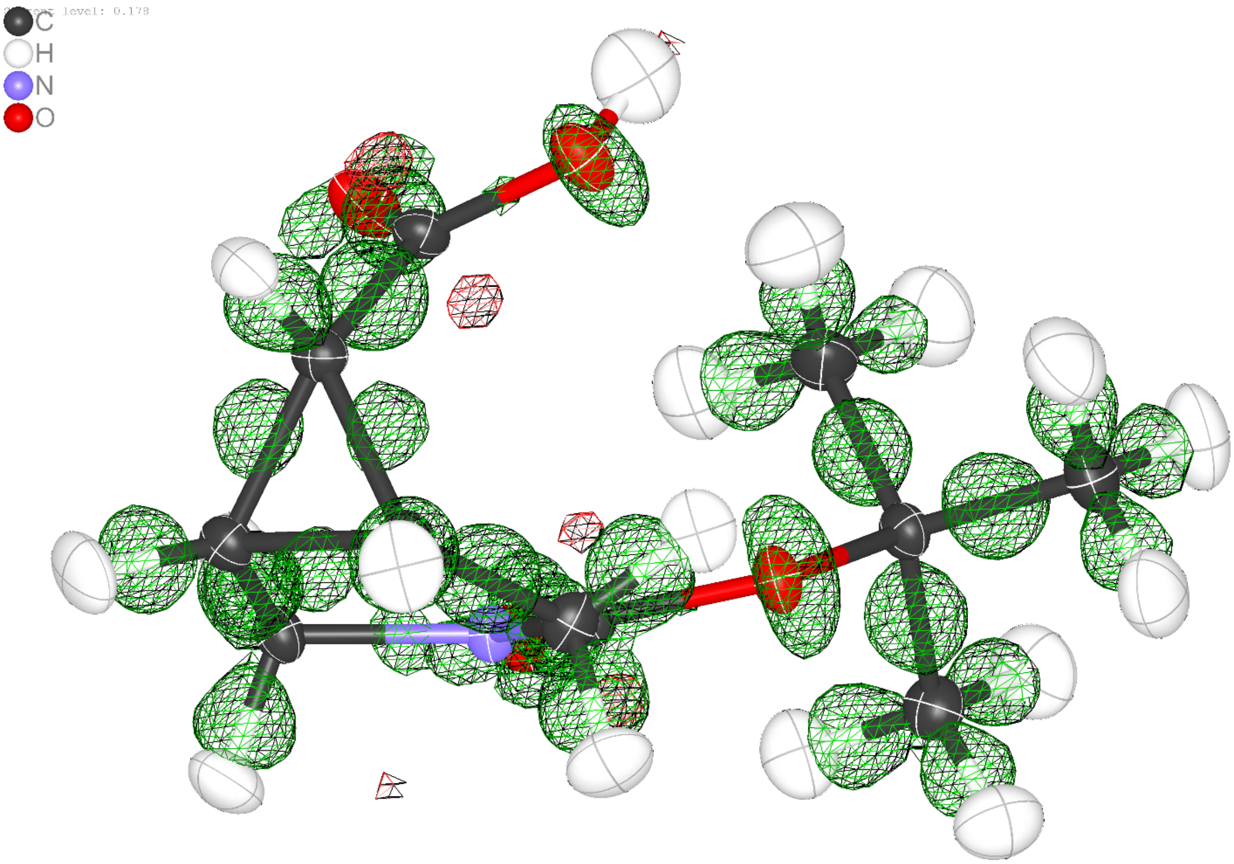

Figure S12. The deformation (bonding) density. The banana bonds of the 3-ring and the lone pair electrons are clearly visible.

Data Plots: Diffraction Data

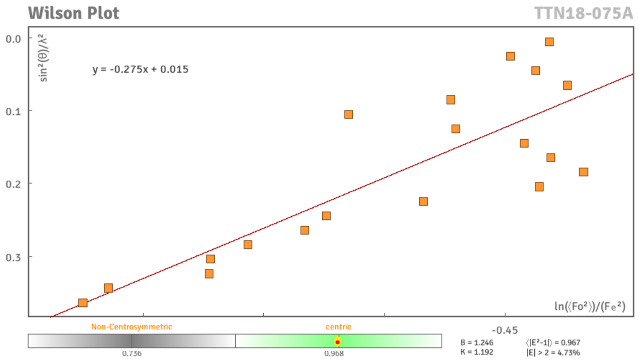

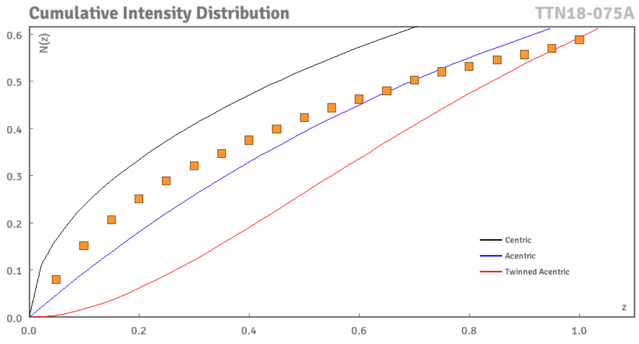

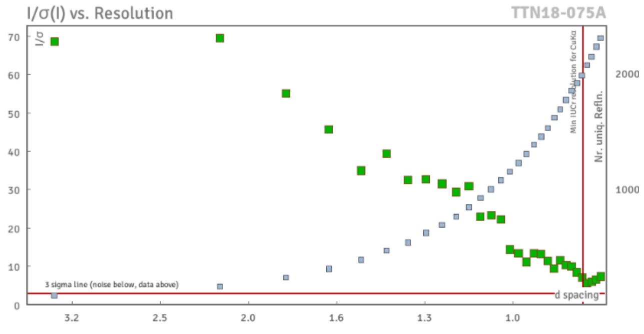

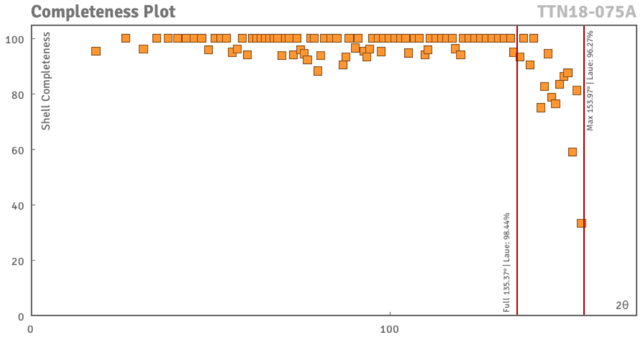

Data Plots: Refinement and Data

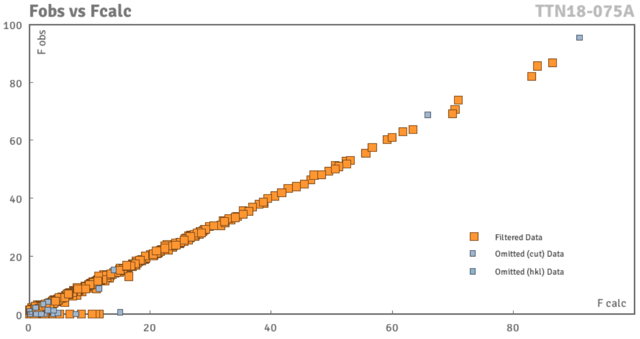

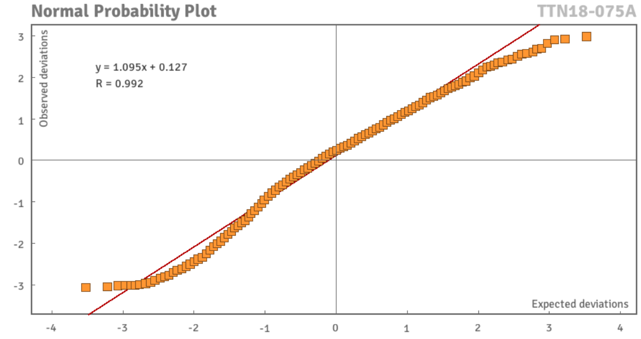

Reflection Statistics

Total reflections (after filtering)	30111	Unique reflections	2323
Completeness	0.963	Mean I/s	23.99
hkl_{max} collected	(11, 13, 14)	hkl_{min} collected	(-11, -14, -13)
hkl_{max} used	(10, 14, 14)	hkl_{min} used	(-11, 0, 0)
Lim d_{max} collected	100.0	Lim d_{min} collected	0.77
d_{max} used	11.83	d_{min} used	0.79
Friedel pairs	3331	Friedel pairs merged	1
Inconsistent equivalents	180	R_{int}	0.069
R_{sigma}	0.0288	Intensity transformed	0
Omitted reflections	0	Omitted by user (OMIT hkl)	580
Multiplicity	(1787, 1227, 1086, 1005, 803, 636, 445, 324, 204, 129, 72, 42, 23, 12, 11)	Maximum multiplicity	36
Removed systematic absences	1127	Filtered off (Shel/OMIT)	0

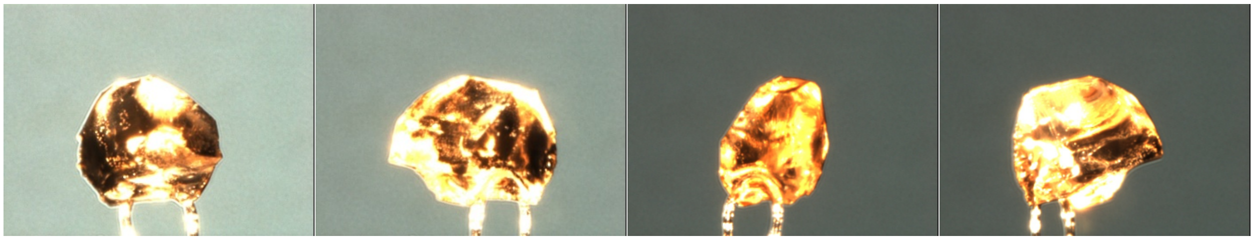

Table S13: Fractional Atomic Coordinates (×10^4) and Equivalent Isotropic Displacement Parameters (Å^2×10^3) for (1R,5S,6s)-3-(*tert*-butoxycarbonyl)-3-azabicyclo[3.1.0]hexane-6-carboxylic acid (*endo*-**11**). U_{eq} is defined as 1/3 of the trace of the orthogonalised U_{ij}.

Atom	x	y	z	U_{eq}
O1	4502.4(6)	2915.4(5)	4075.4(6)	16.21(16)
O2	2733.1(7)	1772.0(5)	4470.7(5)	16.61(16)
O3	1719.2(8)	4357.0(6)	1046.7(7)	20.45(18)
O4	277.5(8)	2969.5(6)	1406.2(6)	23.41(18)
N1	2323.5(8)	3658.5(6)	4215.7(6)	13.61(17)
C1	679.8(10)	3583.9(8)	4134.1(9)	14.9(2)
C2	-83.6(10)	4630.4(8)	3392.7(8)	15.0(2)
C3	1233.6(10)	5406.6(9)	3360.6(8)	14.9(2)
C4	2796.6(10)	4813.1(8)	4048.6(10)	15.2(2)
C5	129.0(10)	4843.5(8)	2170.7(8)	15.0(2)
C6	700.7(10)	3943.6(8)	1522.5(8)	14.6(2)
C7	3167.3(9)	2715.2(7)	4270.7(8)	13.09(18)
C8	5470.8(10)	1967.8(8)	3922.3(8)	15.4(2)
C9	6733.5(11)	2562.5(10)	3578.7(10)	23.4(2)
C10	6226.2(11)	1340.5(9)	5167.3(9)	19.7(2)
C11	4465.3(12)	1203.4(10)	2842.5(9)	23.6(2)

Table S14: Anisotropic Displacement Parameters (×10⁴) for (1R,5S,6s)-3-(*tert*-butoxycarbonyl)-3-azabicyclo[3.1.0]hexane-6-carboxylic acid (*endo*-**11**). The anisotropic displacement factor exponent takes the form: $-2p^2[h^2a^{*2} \times U_{11} + ... + 2hka^* \times b^* \times U_{12}]$

Atom	U_{11}	U_{22}	U_{33}	U_{23}	U_{13}	U_{12}
O1	16.1(3)	11.6(3)	24.4(3)	1.2(2)	11.5(2)	1.9(2)
O2	19.5(3)	10.6(3)	24.1(3)	1.2(2)	13.2(3)	2.0(2)
O3	27.4(4)	14.2(4)	26.5(4)	-2.2(3)	17.8(3)	-2.7(3)
O4	34.6(4)	15.2(4)	27.5(4)	-8.4(3)	19.9(3)	-8.0(3)
N1	13.9(3)	10.2(4)	17.4(4)	1.0(2)	6.6(3)	0.7(3)
C1	15.4(4)	13.7(4)	17.8(4)	2.0(3)	8.6(3)	0.7(3)
C2	16.4(4)	13.1(4)	16.5(4)	2.0(3)	7.5(3)	-0.5(3)
C3	18.2(4)	9.5(5)	17.7(4)	1.9(3)	7.5(3)	-0.7(4)
C4	15.7(4)	9.5(5)	19.0(5)	-0.2(4)	4.9(4)	-1.4(4)
C5	16.5(4)	13.2(5)	15.1(4)	2.3(4)	5.9(3)	0.1(4)
C6	17.7(4)	13.0(5)	14.0(4)	-0.5(4)	6.8(3)	-2.0(4)
C7	14.2(3)	10.7(4)	15.7(4)	1.1(2)	7.2(2)	1.1(2)
C8	14.7(4)	15.3(4)	17.4(4)	2.7(2)	7.5(2)	-0.6(2)
C9	20.1(5)	26.5(6)	28.2(5)	-0.9(4)	14.4(4)	-0.0(4)
C10	19.1(5)	17.7(6)	21.6(4)	3.4(4)	6.7(3)	2.0(3)
C11	23.5(5)	26.5(6)	21.3(5)	-1.8(4)	9.0(4)	-7.8(4)
H1a	25(5)	21(5)	36(5)	-4(3)	15(4)	-9(3)
H1b	33(6)	37(7)	25(4)	-2(5)	18(2)	-4(2)
H2	27(4)	43(8)	35(6)	17(3)	19(2)	8(4)
H3	45(9)	32(10)	49(10)	-1(7)	20(8)	1(8)
H3a	40(7)	7(7)	42(7)	5(5)	16(5)	4(5)
H4a	33(6)	28(7)	28(7)	-4(5)	5(5)	-8(6)
H4b	32(6)	15(6)	35(7)	3(5)	17(6)	-1(5)
H5	25(5)	29(6)	20(4)	11(3)	8(3)	7(3)
H9a	33(5)	39(5)	43(5)	-10(3)	20(2)	-6(2)
H9b	32(5)	42(5)	64(7)	2(2)	29(3)	-6(3)
H9c	40(5)	44(6)	40(4)	2(3)	22(2)	7(2)
H10a	43(5)	33(5)	47(6)	15(2)	20(3)	5(3)
H10b	35(4)	29(6)	40(5)	1(2)	18(2)	5(3)
H10c	38(5)	33(5)	30(4)	-4(2)	8(2)	-2(2)
H11a	49(6)	48(6)	38(4)	-2(3)	10(2)	1(2)
H11b	43(5)	32(6)	51(6)	-9(2)	26(3)	-15(3)
H11c	46(5)	42(5)	62(7)	4(2)	27(3)	-15(3)

Table S15: Bond Lengths in Å for (1R,5S,6s)-3-(*tert*-butoxycarbonyl)-3-azabicyclo[3.1.0]hexane-6-carboxylic acid (*endo*-**11**).

Atom	Atom	Length/Å
O1	C7	1.3379(10)
O1	C8	1.4770(10)
O2	C7	1.2337(10)
O3	C6	1.3333(11)
O3	H3	0.918(14)
O4	C6	1.2063(11)
N1	C1	1.4669(10)
N1	C4	1.4660(11)
N1	C7	1.3427(11)
C1	C2	1.5127(12)
C1	H1a	1.084(10)
C1	H1b	1.089(9)
C2	C3	1.5221(12)
C2	C5	1.5027(12)
C2	H2	1.104(9)
C3	C4	1.5134(12)
C3	C5	1.5069(12)
C3	H3a	1.058(10)
C4	H4a	1.076(10)
C4	H4b	1.096(10)
C5	C6	1.4986(12)
C5	H5	1.082(10)
C8	C9	1.5212(12)
C8	C10	1.5196(12)
C8	C11	1.5247(13)
C9	H9a	1.090(11)
C9	H9b	1.089(11)
C9	H9c	1.060(11)
C10	H10a	1.087(11)
C10	H10b	1.075(10)
C10	H10c	1.088(11)
C11	H11a	1.064(12)
C11	H11b	1.067(11)
C11	H11c	1.096(11)

Table S16: Bond Angles in ° for (1R,5S,6s)-3-(*tert*-butoxycarbonyl)-3-azabicyclo[3.1.0]hexane-6-carboxylic acid (*endo*-**11**).

Atom	Atom	Atom	Angle/°
C8	O1	C7	120.44(7)
H3	O3	C6	109.2(9)
C4	N1	C1	113.01(7)
C7	N1	C1	120.34(7)
C7	N1	C4	126.08(7)
C2	C1	N1	103.17(7)
H1a	C1	N1	108.2(5)
H1a	C1	C2	113.4(5)
H1b	C1	N1	110.4(5)
H1b	C1	C2	112.3(6)
H1b	C1	H1a	109.1(7)
C3	C2	C1	107.93(7)
C5	C2	C1	117.69(7)
C5	C2	C3	59.75(6)
H2	C2	C1	118.9(6)
H2	C2	C3	121.1(6)
H2	C2	C5	117.5(5)
C4	C3	C2	107.61(8)
C5	C3	C2	59.49(6)
C5	C3	C4	116.07(8)
H3a	C3	C2	122.0(5)
H3a	C3	C4	119.2(5)
H3a	C3	C5	118.4(6)
C3	C4	N1	103.58(7)
H4a	C4	N1	111.4(6)
H4a	C4	C3	110.5(5)
H4b	C4	N1	110.3(5)
H4b	C4	C3	112.5(5)
H4b	C4	H4a	108.5(7)
C3	C5	C2	60.76(6)
C6	C5	C2	122.14(8)
C6	C5	C3	121.49(7)
H5	C5	C2	115.9(5)
H5	C5	C3	115.1(5)
H5	C5	C6	112.4(5)
O4	C6	O3	123.23(8)
C5	C6	O3	111.53(8)
C5	C6	O4	125.22(8)
O2	C7	O1	124.50(8)
N1	C7	O1	112.66(7)
N1	C7	O2	122.84(7)
C9	C8	O1	102.91(7)
C10	C8	O1	109.78(7)
C10	C8	C9	110.45(7)
C11	C8	O1	110.09(7)
C11	C8	C9	110.25(8)
C11	C8	C10	112.91(8)
H9a	C9	C8	112.6(5)
H9b	C9	C8	108.5(6)
H9b	C9	H9a	107.0(8)
H9c	C9	C8	109.1(6)
H9c	C9	H9a	107.1(9)
H9c	C9	H9b	112.6(8)
H10a	C10	C8	108.8(6)
H10b	C10	C8	110.4(5)
H10b	C10	H10a	109.8(8)
H10c	C10	C8	109.7(6)
H10c	C10	H10a	107.6(8)
H10c	C10	H10b	110.5(8)
H11a	C11	C8	109.5(6)
H11b	C11	C8	113.0(6)
H11b	C11	H11a	108.9(9)
H11c	C11	C8	108.3(6)
H11c	C11	H11a	110.9(9)
H11c	C11	H11b	106.1(9)

Table S17: Torsion Angles in ° for (1R,5S,6s)-3-(*tert*-butoxycarbonyl)-3-azabicyclo[3.1.0]hexane-6-carboxylic acid (*endo*-**11**).

Atom	Atom	Atom	Atom	Angle/°
O1	C7	N1	C1	166.84(6)
O1	C7	N1	C4	-3.94(9)
O2	C7	N1	C1	-12.62(10)
O2	C7	N1	C4	176.60(8)
O3	C6	C5	C2	-139.63(7)
O3	C6	C5	C3	-66.59(8)
O4	C6	C5	C2	42.17(10)
O4	C6	C5	C3	115.22(9)
N1	C1	C2	C3	-13.81(7)
N1	C1	C2	C5	50.84(8)
N1	C4	C3	C2	11.08(8)
N1	C4	C3	C5	-52.96(8)
C1	C2	C3	C4	1.76(8)
C1	C2	C3	C5	112.13(10)
C1	C2	C5	C3	-95.53(10)
C1	C2	C5	C6	15.28(9)
C2	C3	C5	C6	-111.84(6)
C2	C5	C3	C4	95.89(7)

Table S18: Hydrogen Fractional Atomic Coordinates (×10[4]) and Equivalent Isotropic Displacement Parameters (Å[2]×10[3]) for (1R,5S,6s)-3-(*tert*-butoxycarbonyl)-3-azabicyclo[3.1.0]hexane-6-carboxylic acid (*endo*-**11**). U_{eq} is defined as 1/3 of the trace of the orthogonalised U_{ij}.

Atom	x	y	z	U_{eq}
H1a	174(11)	2802(9)	3656(9)	26(3)
H1b	644(11)	3577(9)	5075(9)	29(3)
H2	-1159(11)	4974(9)	3488(10)	33(3)
H3	1983(16)	3799(12)	604(13)	41(4)
H3a	1171(12)	6295(9)	3436(10)	29(3)
H4a	3431(12)	5214(9)	4944(10)	32(3)
H4b	3560(11)	4804(8)	3503(9)	26(3)
H5	-735(11)	5398(9)	1509(9)	24(3)
H9a	7471(13)	3128(10)	4322(10)	37(3)
H9b	7517(13)	1925(10)	3443(11)	43(3)
H9c	6173(13)	3063(10)	2761(11)	39(3)
H10a	6984(13)	675(10)	5051(10)	40(3)
H10b	5328(12)	979(9)	5449(10)	33(3)
H10c	6974(12)	1916(9)	5890(10)	35(3)
H11a	3892(14)	1698(11)	2018(11)	47(3)
H11b	3589(13)	742(10)	3054(10)	40(3)
H11c	5245(13)	570(11)	2678(11)	48(3)

Table S19: Hydrogen Bond information for (1R,5S,6s)-3-(*tert*-butoxycarbonyl)-3-azabicyclo[3.1.0]hexane-6-carboxylic acid (*endo*-**11**).

D	H	A	d(D-H)/Å	d(H-A)/Å	d(D-A)/Å	D-H-A/deg
O3	H3	O2[1]	0.918(14)	1.808(14)	2.6726(9)	155.9(13)

[1]+x,1/2-y,-1/2+z

Citations

CrysAlisPro Software System, Rigaku Oxford Diffraction, (2023).

L.J. Bourhis and O.V. Dolomanov and R.J. Gildea and J.A.K. Howard and H. Puschmann, The Anatomy of a Comprehensive Constrained, Restrained, Refinement Program for the Modern Computing Environment - Olex2 Disected, *Acta Cryst. A*, (2015), **A71**, 59-71.

O.V. Dolomanov and L.J. Bourhis and R.J. Gildea and J.A.K. Howard and H. Puschmann, Olex2: A complete structure solution, refinement and analysis program, *J. Appl. Cryst.*, (2009), **42**, 339-341.

Sheldrick, G.M., ShelXT-Integrated space-group and crystal-structure determination, *Acta Cryst.*, (2015), **A71**, 3-8.

13. Cost Analysis of Rhodium Catalyst Loading vs. *Exo*-11

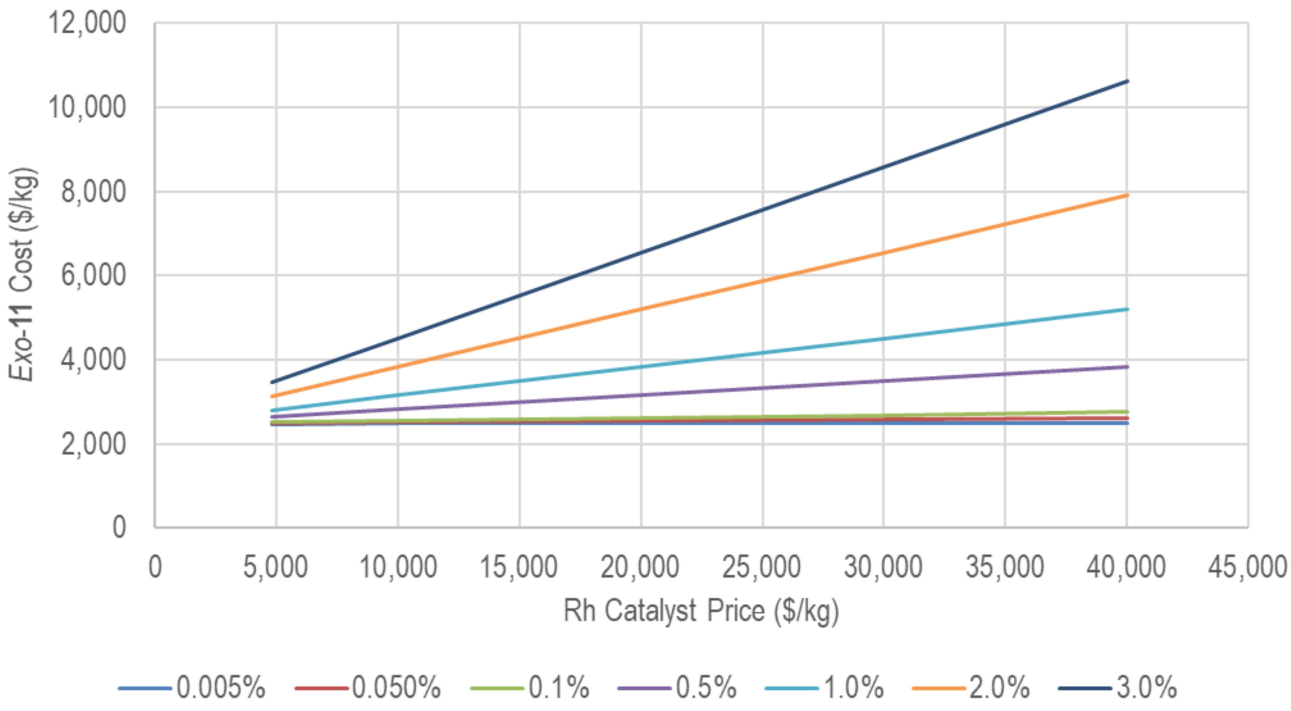

Figure S13. Cost Analysis of Rhodium Catalyst Loading vs. *exo*-**11**. At 0.005 mol % catalyst loading, the price of *exo*-**11** remains stable as the price of rhodium catalyst, $Rh_2(esp)_2$, increases.

14. References

(1) Green, S. P.; Wheelhouse, K. M.; Payne, A. D.; Hallett, J. P.; Miller, P. W.; Bull, J. A. Thermal Stability and Explosive Hazard Assessment of Diazo Compounds and Diazo Transfer Reagents. *Org. Process Res. Dev.* **2020**, *24* (1), 67-84, Article. DOI: 10.1021/acs.oprd.9b00422.

(2) Stürmer, R.; Schäfer, B.; Wolfart, V.; Stahr, H.; Kazmaier, U.; Helmchen, G. A Short and Efficient Synthesis of (S)-1-Boc-2,5-dihydro-1H-pyrrole-2-carboxylic Acid. *Synthesis* **2001**, *2001* (01), 0046-0048. DOI: 10.1055/s-2001-9755.
(3) Davies, H. M. L.; Bruzinski, P. R.; Lake, D. H.; Kong, N.; Fall, M. J. Asymmetric Cyclopropanations by Rhodium(II) N-(Arylsulfonyl)prolinate Catalyzed Decomposition of Vinyldiazomethanes in the Presence of Alkenes. Practical Enantioselective Synthesis of the Four Stereoisomers of 2-Phenylcyclopropan-1-amino Acid. *Journal of the American Chemical Society* **1996**, *118* (29), 6897-6907. DOI: 10.1021/ja9604931.
(4) Fu, J.; Ren, Z.; Bacsa, J.; Musaev, D. G.; Davies, H. M. L. Desymmetrization of cyclohexanes by site- and stereoselective C–H functionalization. *Nature* **2018**, *564* (7736), 395-399. DOI: 10.1038/s41586-018-0799-2.
(5) Reddy, R. P.; Davies, H. M. L. Dirhodium Tetracarboxylates Derived from Adamantylglycine as Chiral Catalysts for Enantioselective C−H Aminations. *Organic Letters* **2006**, *8* (22), 5013-5016. DOI: 10.1021/ol061742l.
(6) Garlets, Z. J.; Boni, Y. T.; Sharland, J. C.; Kirby, R. P.; Fu, J.; Bacsa, J.; Davies, H. M. L. Design, Synthesis, and Evaluation of Extended C4–Symmetric Dirhodium Tetracarboxylate Catalysts. *ACS Catalysis* **2022**, *12* (17), 10841-10848. DOI: 10.1021/acscatal.2c03041.
(7) Müller, P.; Allenbach, Y.; Robert, E. Rhodium(II)-catalyzed olefin cyclopropanation with the phenyliodonium ylide derived from Meldrum's acid. *Tetrahedron: Asymmetry* **2003**, *14* (7), 779-785. DOI: https://doi.org/10.1016/S0957-4166(03)00029-6.
(8) Qin, C.; Davies, H. M. L. Role of Sterically Demanding Chiral Dirhodium Catalysts in Site-Selective C–H Functionalization of Activated Primary C–H Bonds. *Journal of the American Chemical Society* **2014**, *136* (27), 9792-9796. DOI: 10.1021/ja504797x.
(9) Li, T.; Fan, L.; Gong, H.; Xia, Z.; Zhu, Y.; Jiang, N.; Jiang, L.; Liu, G.; Li, Y.; Wang, J. Janusarene: A Homoditopic Molecular Host. *Angewandte Chemie International Edition* **2017**, *56* (32), 9473-9477. DOI: https://doi.org/10.1002/anie.201705451.
(10) Dhayalan, V.; Gadekar, S. C.; Alassad, Z.; Milo, A. Unravelling mechanistic features of organocatalysis with in situ modifications at the secondary sphere. *Nature Chemistry* **2019**, *11* (6), 543-551. DOI: 10.1038/s41557-019-0258-1.
(11) Kim, J. H.; Nam, G. Synthesis and evaluation of 6-pyrazoylamido-3N-substituted azabicyclo[3,1,0]hexane derivatives as T-type calcium channel inhibitors for treatment of neuropathic pain. *Bioorganic & Medicinal Chemistry* **2016**, *24* (21), 5028-5035. DOI: https://doi.org/10.1016/j.bmc.2016.06.006.
(12) Wei, B.; Sharland, J. C.; Lin, P.; Wilkerson-Hill, S. M.; Fullilove, F. A.; McKinnon, S.; Blackmond, D. G.; Davies, H. M. L. In Situ Kinetic Studies of Rh(II)-Catalyzed Asymmetric Cyclopropanation with Low Catalyst Loadings. *ACS Catalysis* **2020**, *10* (2), 1161-1170. DOI: 10.1021/acscatal.9b04595.
